# Automated Identification of Postoperative Infections to Allow Prediction and Surveillance Based on Electronic Health Record Data: Scoping Review

**DOI:** 10.2196/57195

**Published:** 2024-09-10

**Authors:** Siri Lise van der Meijden, Anna M van Boekel, Harry van Goor, Rob GHH Nelissen, Jan W Schoones, Ewout W Steyerberg, Bart F Geerts, Mark GJ de Boer, M Sesmu Arbous

**Affiliations:** 1 Intensive Care Unit Leiden University Medical Center Leiden Netherlands; 2 Healthplus.ai BV Amsterdam Netherlands; 3 General Surgery Department Radboud University Medical Center Nijmegen Netherlands; 4 Department of Orthopedics Leiden University Medical Center Leiden Netherlands; 5 Directorate of Research Policy Leiden University Medical Center Leiden Netherlands; 6 Department of Biomedical Data Sciences Leiden University Medical Center Leiden Netherlands; 7 Department of Infectious Diseases Leiden University Medical Center Leiden Netherlands

**Keywords:** postoperative infections, surveillance, prediction, surgery, artificial intelligence, chart review, electronic health record, scoping review, postoperative, surgical, infection, infections, predictions, predict, predictive, bacterial, machine learning, record, records, EHR, EHRs, synthesis, review methods, review methodology, search, searches, searching, scoping

## Abstract

**Background:**

Postoperative infections remain a crucial challenge in health care, resulting in high morbidity, mortality, and costs. Accurate identification and labeling of patients with postoperative bacterial infections is crucial for developing prediction models, validating biomarkers, and implementing surveillance systems in clinical practice.

**Objective:**

This scoping review aimed to explore methods for identifying patients with postoperative infections using electronic health record (EHR) data to go beyond the reference standard of manual chart review.

**Methods:**

We performed a systematic search strategy across PubMed, Embase, Web of Science (Core Collection), the Cochrane Library, and Emcare (Ovid), targeting studies addressing the prediction and fully automated surveillance (ie, without manual check) of diverse bacterial infections in the postoperative setting. For prediction modeling studies, we assessed the labeling methods used, categorizing them as either manual or automated. We evaluated the different types of EHR data needed for the surveillance and labeling of postoperative infections, as well as the performance of fully automated surveillance systems compared with manual chart review.

**Results:**

We identified 75 different methods and definitions used to identify patients with postoperative infections in studies published between 2003 and 2023. Manual labeling was the predominant method in prediction modeling research, 65% (49/75) of the identified methods use structured data, and 45% (34/75) use free text and clinical notes as one of their data sources. Fully automated surveillance systems should be used with caution because the reported positive predictive values are between 0.31 and 0.76.

**Conclusions:**

There is currently no evidence to support fully automated labeling and identification of patients with infections based solely on structured EHR data. Future research should focus on defining uniform definitions, as well as prioritizing the development of more scalable, automated methods for infection detection using structured EHR data.

## Introduction

Postoperative bacterial infections, including deep or superficial surgical site infections (SSIs), urinary tract infections (UTIs), and pneumonia, are the most frequent complications after surgery. Postoperative infections can be categorized into subtypes, usually based on location or severity according to the Clavien-Dindo classification [1]. The overall incidence of postoperative infections within 30 days of surgery varies between 6.5% and 25% [2-4]. Considering the 313 million patients undergoing surgery globally each year, these postoperative infections have an enormous impact on population health and overall health care costs [5]. Effective postoperative infection prevention and management require early detection of high-risk patients through prediction and data-driven surveillance. It is imperative for developing and validating prediction and surveillance systems to be able to accurately identify patients who have postoperative infections. Machine learning modeling practices use the term “labeling” for the identification of patients with the outcome of interest. Labeling and surveillance are both challenges due to underreporting in (hospital) complication registries, ranging from 38% to 77% when compared with a manual chart review [6,7]. Consequently, the current reference standard for identifying patients with postoperative infections relies on labor-intensive manual chart review, with an estimated 1.5 full-time equivalents per 10,000 admissions [8,9]. Furthermore, manual surveillance and labeling are prone to interobserver variability [10,11] and human errors [12], highlighting the need for more robust methods to address this devastating postoperative problem.

To achieve a more objective, cost-effective, and resource-efficient identification of patients with postoperative infections, it is imperative to leverage the electronic health records (EHRs) to automatically detect patients with infections without human checking on high-risk patients based on readily available EHR data. Different types of data are present within the EHR, including structured, tabular, and free-text records in which diagnoses and clinical symptoms are reported. A previously performed systematic review identified semiautomated and fully automated surveillance methods for hospital-acquired infections (HAIs) [13]. As more than 90% of the included systems required manual checking of infectious cases, it was concluded that fully automated surveillance of HAIs cannot be routinely used yet in health care settings.

To go beyond manual labeling and manual surveillance and to explore the current methods and criteria used in prediction modeling studies, the aim of this study was to perform a scoping review on available labeling methods for postoperative infections and fully automated surveillance systems (ie, not requiring manual checking). We aimed to (1) evaluate the current methods and criteria used to label patients with postoperative infections in prediction modeling and biomarker validation studies, (2) explore available automated surveillance methods and their performance (sensitivity, specificity, positive predictive value [PPV], and negative predictive value [NPV]) in comparison with reference standard manual chart review, and (3) determine the necessary data types and sources needed to perform automated detection of postoperative infections.

## Methods

### Overview

This scoping review combined 2 literature searches to evaluate current methods used by prediction modeling and biomarker validation studies to label patients with postoperative infections and the use of automated surveillance systems to identify patients with postoperative infections based on EHR data. The PRISMA-ScR (Preferred Reporting Items for Systematic Reviews and Meta-Analyses Extension for Scoping Reviews) checklist was used. The protocol was registered on Open Science Framework [14].

### Search Strategy

**Figure 1:**
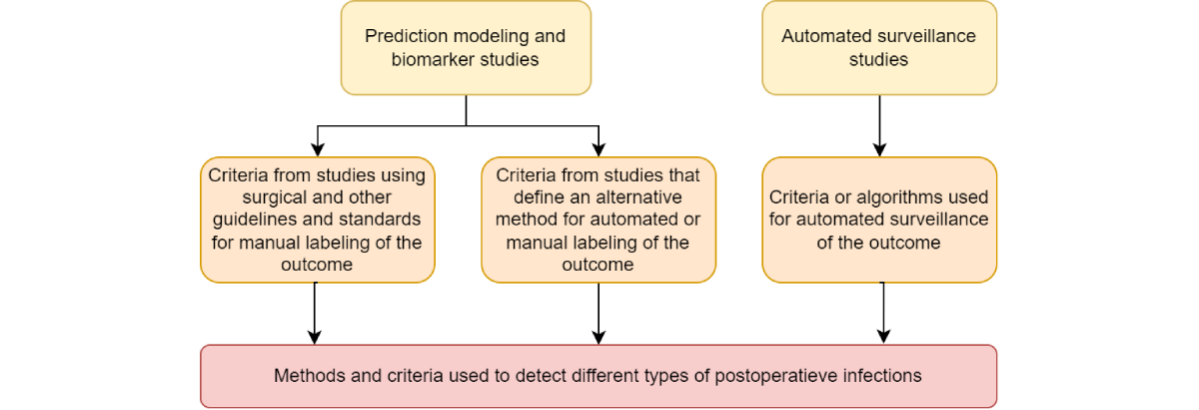
Data sources from the literature for identifying infections in prediction modeling or biomarker studies and automated surveillance studies.

First, prediction modeling validation studies using machine learning methods, statistical models, and biomarkers to predict postoperative infections were identified. Second, a separate search was performed to identify studies on automated surveillance for postoperative and other hospital-acquired infections (Figure 1). Surveillance studies focusing on surgical populations often only investigate SSIs. As we aimed to study all bacterial infections that may occur after surgery, surveillance studies in a hospital-wide setting were also included. Both searches were performed in PubMed, Embase (OVID), Web of Science (Core Collection), the Cochrane Library, and Emcare (OVID). Studies were included from inception (ie, 1966) to August 1, 2023. The search queries were generated with help from an information specialist (JWS) from the Leiden University Medical Center. The details of the search queries are provided in Appendix A of Multimedia Appendix 1.

### Selection Criteria

The selection of studies was performed in Covidence, a program used to manage systematic literature searches. The inclusion and exclusion criteria used are presented in Table S1 in Multimedia Appendix 1. All titles and abstracts were screened by 2 independent reviewers (AMVB and BFG for prediction models; SLVDM and BFG for surveillance studies). The full texts of all potentially relevant studies were retrieved and assessed by 2 reviewers (SLVDM and BFG) for eligibility. Any disagreement on the inclusion or exclusion of studies was resolved through reassessment and discussion with a third reviewer (MSA). The data from the different reports were collected by 1 researcher (AMVB or SLVDM), and inconsistencies were checked for by a second researcher (BFG).

### Data Extraction and Definitions

The following data were extracted for the prediction modeling studies: name of the prediction tool, type of prediction tool (machine learning, biomarker, and statistical model), surgical subpopulations, type of postoperative infection predicted, and criteria and guidelines used to manually or automatically label patients with infections. Manual labeling involves individuals conducting EHR chart reviews and applying specific criteria, often derived from surgical guidelines, to determine the presence or absence of infections in patient records. The criteria for diagnosing patients with an infection, for example, from a reference guideline from the literature, were identified and extracted.

For automated surveillance studies, the population, study design, years of data collection, type of infection surveyed, type of algorithm used, definition used to automatically detect infections, reference standard used to compare the automated method with, type of validation performed, and performance metrics reported compared with the reference standard were collected. The main metrics used to assess performance were the method’s sensitivity, specificity, PPV, and NPV. Other metrics extracted are presented in Table S17 in Multimedia Appendix 1, including the area under the receiver operating characteristic curve, accuracy, *F*_1_-score, κ score, Pearson correlation coefficient, and agreement percentage. Only performance metrics were assessed for surveillance studies, as for prediction modeling studies, and no accuracy of the labeling method compared with a reference standard was determined.

### Data Synthesis

For each method to identify and label patients with infections, the data type categories needed from the EHR were assessed to identify infections based on the definition used. These could be structured EHR data (type A), including tabular information stored, such as complication registries, medication information, and vital signs; free-text clinical notes (type B), including all clinical information stored in free-text, such as discharge letters and daily reports; microbiology results (type C), which is seen as a separate category, as it differs per hospital how well-structured this information is stored [15]; and an additional interpretation layer (whether the results are positive) is needed to use this information; or imaging results (type D), or a combination of these categories. The definitions were further differentiated based on the data types and criteria needed to adhere to the definitions in Appendix E in Multimedia Appendix 1.

Some prediction models and surveillance systems are focused on predicting or detecting all severity types of bacterial infections, while others focus only on infections requiring pharmacological or surgical treatment. For example, some definitions include the prescription of antibiotics as one of the criteria, while others base their criteria on clinical symptoms only. As the severity of the infections surveyed or predicted influences the intended use case and number of infections identified, we classified the definitions according to the Clavien-Dindo scale [1]. Finally, the performance of the automated infection surveillance systems compared with that of the reference standard manual review was visualized per subtype of infection. The results were grouped according to the type of infection, such as HAI (type not further specified), SSI, pneumonia, anastomotic leakage and abdominal infections, bloodstream infections (including central venous catheter-related infections and sepsis), and UTIs. Infections that did not belong to one of these groups were categorized as “other.”

## Results

### Overview

**Figure 2:**
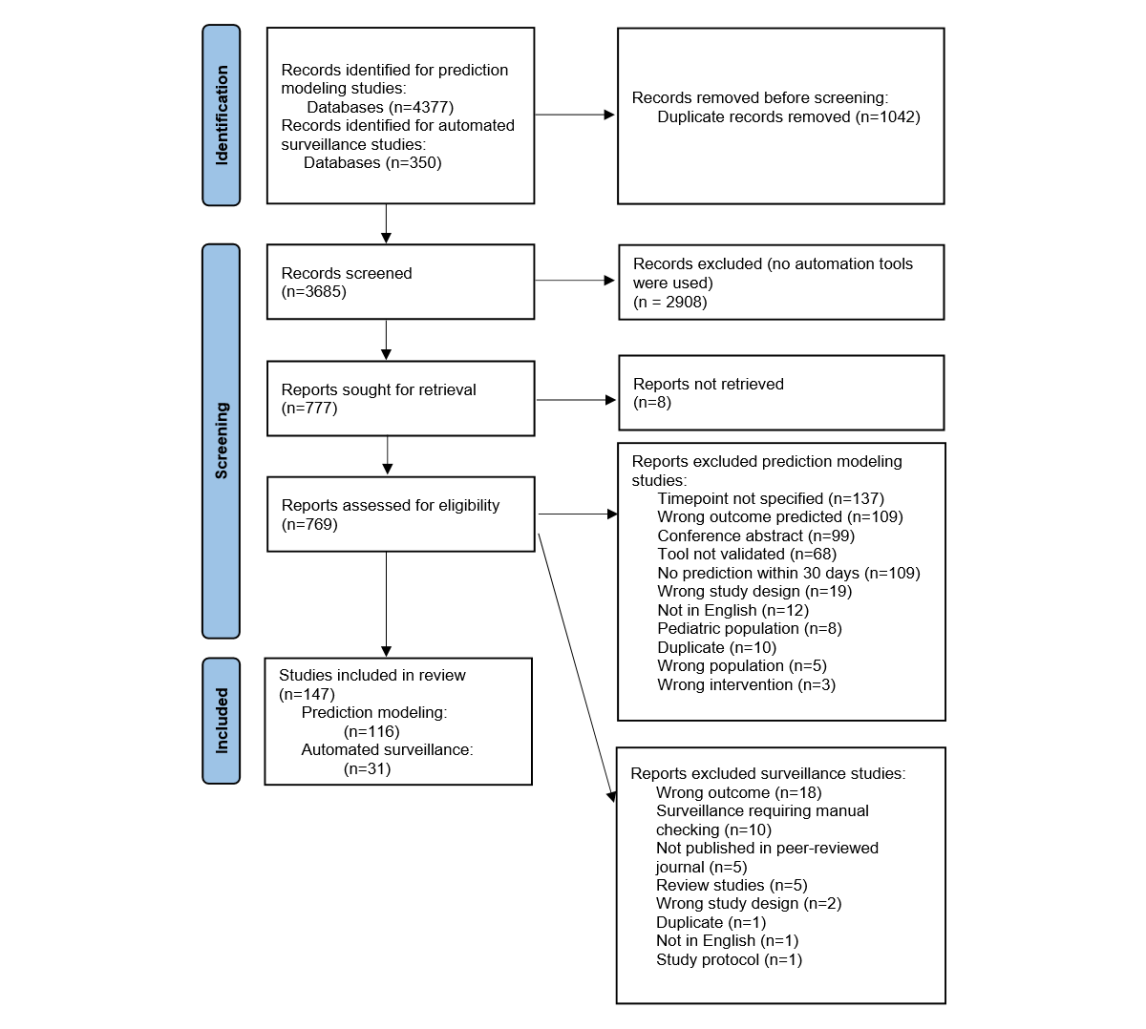
PRISMA (Preferred Reporting Items for Systematic Reviews and Meta-Analyses) diagram.

We included a total of 147 studies published between 2003 and 2023 (Figure 2). Of these, 116 studies focused on the prediction of postoperative infections; either the development and validation of prediction models or a predictive biomarker were performed, or validation was performed of preexisting risk scores. These included the American College of Surgeons National Surgical Quality Improvement Program surgical risk calculator (ACS NSQIP; 33/116 studies), the National Nosocomial Infection Surveillance System (NNIS; 4/116 studies), and the Surgical Risk Preoperative Assessment System (SURPAS; 5/116 studies).

Out of 116 studies, 4 did not report the methodology used to determine which patients had the outcome of interest (ie, postoperative infection). In 83% (97/116) of prediction modeling studies, manual labeling based on diagnostic guidelines was performed, or a publicly available, manually labeled database was used, such as the participant use data file from the ACS NSQIP program (Table 1). A total of 13% (15/116) of studies used an alternative, non–guideline-based method to label patients with infections, 11 of whom used manual labeling, 3 of whom did not explicitly mention manual or automatic labeling, and 1 of whom used automatic labeling. In total, 93% (108/116) of the prediction modeling studies used manual labeling to determine the outcome of interest or a manually labeled, publicly available data set to perform their research.

**Table 1:** Definitions of patients with bacterial infections.

Type of infection and reference		Origin of definition	Type A (structured)^a^	Type B (free-text)^b^	Type C (microbiology results)	Type D (imaging results)	Minimum Clavien-Dindo
**HAI^c^**							
	WHO^d^ [16]	Diagnostic guidelines	✓	✓	✓	✓	1
	ECDC^e^ [17]	Diagnostic guidelines	✓	✓			1
	Ehrentraut et al [18]	Automated surveillance		✓			1
	Sakji et al [19]	Automated surveillance		✓			1
	Tvardik et al [20]	Automated surveillance		✓			1
**Pneumonia**							
	ECDC/ASC NSQIP^f^ [21]	Diagnostic guidelines	✓	✓	✓	✓	1
	Kinlin et al [22]	Prediction modeling	✓	✓	✓	✓	1
	Blacky et al [23]	Automated surveillance	✓		✓		1
	Bouzbid et al [24]	Automated surveillance	✓		✓		2
	Cato et al [25]	Automated surveillance	✓		✓		1
	FitzHenry et al [26]	Automated surveillance		✓			1
	Tvardik et al [20]	Automated surveillance		✓			1
	Colborn et al [27]	Automated surveillance	✓				1
	Stern et al [28]	Automated surveillance	✓			✓	1
**SSI^g^**							
	CDC^h^/ASC NSQIP [29]	Diagnostic guidelines	✓	✓	✓	✓	1-3a
	WHO [30]	Diagnostic guidelines	✓	✓	✓	✓	1-3a
	Daneman et al [31]	Prediction modeling	✓				1
	Weller et al [32]	Prediction modeling	✓				2
	Crispin et al [33]	Prediction modeling	✓				3a
	Martin et al [34]	Prediction modeling	✓	✓			2
	Campillo-Gimenez et al [35]	Automated surveillance	✓	✓			1
	Cato et al [25]	Automated surveillance	✓		✓		1
	FitzHenry et al [26]	Automated surveillance		✓			1
	Leclère et al [36]	Automated surveillance	✓		✓		1
	Leth et al [37]	Automated surveillance	✓		✓		2
	Suzuki et al [38]	Automated surveillance	✓	✓	✓		2
	Tvardik et al [20]	Automated surveillance		✓			1
	Thirukumaran et al [39]	Automated surveillance		✓			1
	Colborn et al [27]	Automated surveillance	✓				1
**Abdominal and AL^i^**							
	Rahbari et al [40]	Diagnostic guidelines	✓	✓		✓	1
	Stidham et al [41]	Prediction modeling	✓				3a
	Miyakita et al [42]	Prediction modeling	✓				3b
	Mckenna et al [43]	Prediction modeling	✓				2
	Nudel et al [44]	Prediction modeling	✓	✓			3a
	Kawai et al [45]	Prediction modeling		✓		✓	2
	Lin et al [46]	Prediction modeling		✓		✓	2
	Shi et al [47]	Prediction modeling				✓	1
	van Kooten et al [48]	Prediction modeling		✓		✓	1
**UTI^j^**							
	ECDC/ASC NSQIP [49]	Diagnostic guidelines	✓	✓	✓		2
	Cheng et al [50]	Prediction modeling		✓	✓		1
	Bouam et al [51]	Automated surveillance			✓		1
	Bouzbid et al [24]	Automated surveillance	✓		✓		2
	Branch-Elliman et al [52]	Automated surveillance	✓	✓			1
	Cato et al [25]	Automated surveillance	✓		✓		1
	Choudhuri et al [53]	Automated surveillance	✓		✓		1
	FitzHenry et al [26]	Automated surveillance		✓			1
	Leth et al [37]	Automated surveillance	✓		✓		2
	Redder et al [54]	Automated surveillance	✓		✓		2
	Tvardik et al [20]	Automated surveillance		✓			1
	van der Werff et al [55]	Automated surveillance	✓	✓	✓		2
	Venable and Dissanaike [56]	Automated surveillance	✓	✓			—^k^
	Wald et al [57]	Automated surveillance	✓		✓		1
	Colborn et al [27]	Automated surveillance	✓				1
**Bloodstream infections**							
	Moore et al [58]	Diagnostic guidelines	✓		✓		1
	Singer et al [59] (sepsis-3 criteria)	Diagnostic guidelines	✓				2
	Blacky et al [23]	Automated surveillance	✓		✓		1
	Bouam et al [51]	Automated surveillance			✓		1
	Bouzbid et al [24]	Automated surveillance	✓		✓		2
	Cato et al [25]	Automated surveillance	✓		✓		1
	FitzHenry et al [26]	Automated surveillance		✓			1
	Leal et al [60]	Automated surveillance			✓		1
	Leal et al [61]	Automated surveillance			✓		1
	Lin et al [62]	Automated surveillance			✓		1
	Redder et al [54]	Automated surveillance	✓		✓		2
	Tvardik et al [20]	Automated surveillance		✓			1
	Valik et al [63]	Automated surveillance	✓		✓		2
	Venable and Dissanaike [56]	Automated surveillance	✓	✓			—^k^
	Woeltje et al [64]	Automated surveillance	✓		✓		1
	Colborn et al [27]	Automated surveillance	✓				1
***Clostirdrium difficile***							
	Dubberke et al [65]	Automated surveillance			✓		1
***Clostridium difficile***							
	van der Werff et al [66]	Automated surveillance			✓		1
**External ventricular and lumbar drain-related meningitis**							
	van Mourik et al [67]	Automated surveillance	✓		✓		1
**MRSA^l^**							
	Peterson et al [68]	Automated surveillance			✓		1
**PJI^m^**							
	Fu et al [69]	Automated surveillance		✓			1
**Neurological**							
	Cheng et al [70]	Prediction modeling	✓	✓	✓		1

^a^Type A (structured): structured electronic health record data, including tabular information stored such as complication registries, medication information, and vital signs.

^b^Type B (free-text): free-text clinical notes, including all clinical information stored in free-text such as discharge letters and daily reports.

^c^HAI: hospital-acquired infections.

^d^WHO: World Health Organization.

^e^ECDC: European Centre for Disease Prevention and Control.

^f^ACS NSQIP: American College of Surgeons National Surgical Quality Improvement Program.

^g^SSI: surgical site infection.

^h^CDC: Centers for Disease Control and Prevention.

^i^AL: anastomotic leakage.

^j^UTI: urinary tract infection.

^k^Not applicable.

^l^MRSA: Methicillin-resistant *Staphylococcus aureus*.

^m^PJI: prosthetic joint infection.

### Automated Surveillance

**Figure 3:**
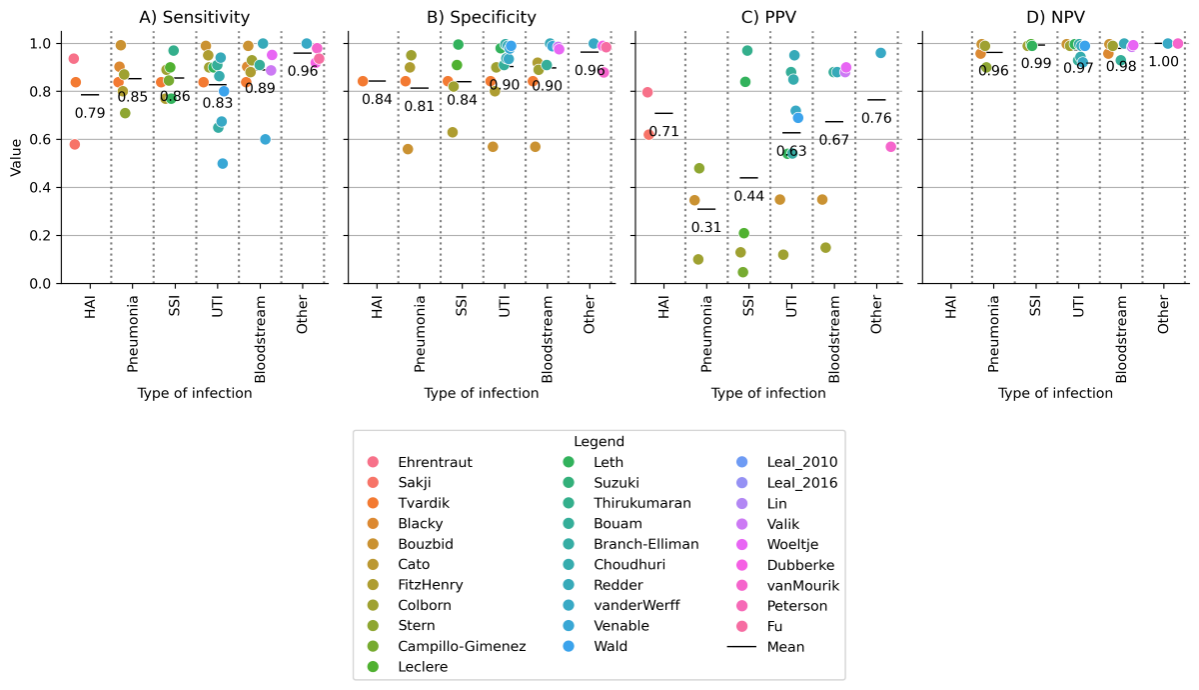
Performance of automated surveillance of postoperative infections compared with manual reference standard chart review. Panel A is the sensitivity, B is the specificity, C is the PPV, and D is the NPV. HAI: hospital-acquired infection; NPV: negative predictive value; PPV: positive predictive value; SSI: surgical site infection; UTI: urinary tract infection.

**Figure 4:**
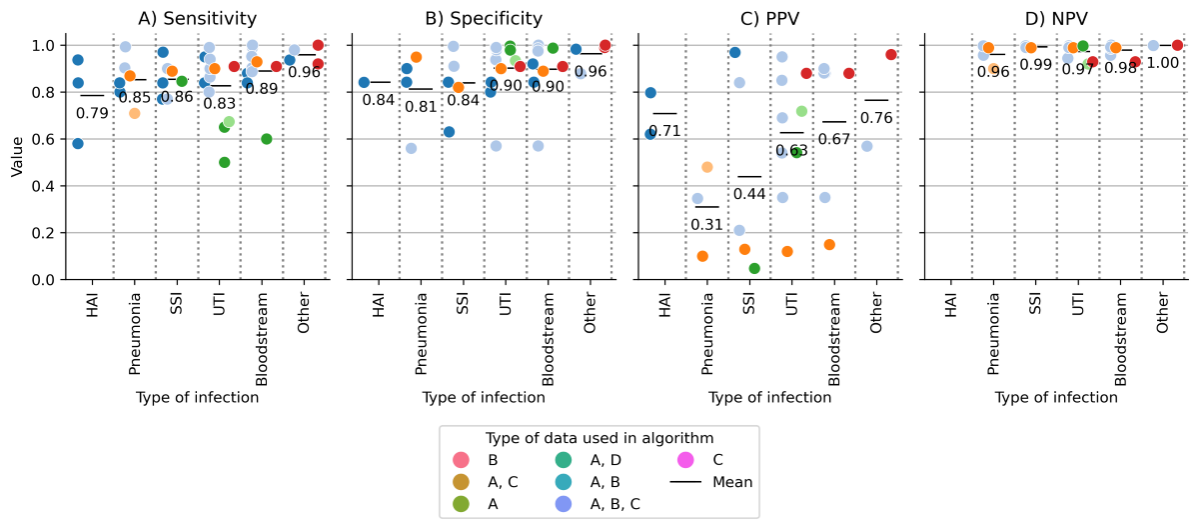
Performance per data type category used in automated surveillance algorithms. A=Structured electronic health record data only (eg, registrations and medication), B=Free-text clinical notes, C=microbiology results. Panel A is the sensitivity, B is the specificity, C is the PPV and D is the NPV. HAI: hospital-acquired infection; NPV: negative predictive value; PPV: positive predictive value; SSI: surgical site infection; UTI: urinary tract infection.

We included 31 automated surveillance studies for bacterial infections. Surveillance was performed and reported per patient, admission, procedure, patient days, or culture. Different types of surveillance systems were studied, and some studies have reported on more than 1 method. Most often (21/31, 68%), a set of criteria or rules was defined to automatically detect infections based on EHR data, followed by natural language processing (NLP) algorithms for free-text from the EHR (7/31, 23%) and other classification algorithms such as logistic regression (3/31, 10%). Except for one study [25], all the studies validated their automated surveillance algorithms against a reference standard (manual chart review, often according to one of the established diagnostic guidelines). Comparing the automated surveillance algorithm to manual chart review according to the established guidelines resulted in a range of sensitivity (0.79-0.96), specificity (0.81-0.96), PPV (0.31-0.76), and NPV (0.96-1.00) estimates for the different types of infection (Figure 3). The performance of all the combinations of postoperative infection data needed to run the automated surveillance algorithm varied (Figure 4). Reported performance per surveillance algorithm is provided in Table S17 in Multimedia Appendix 1.

### Electronic Health Record Data for Automated Identification and Surveillance

**Figure 5:**
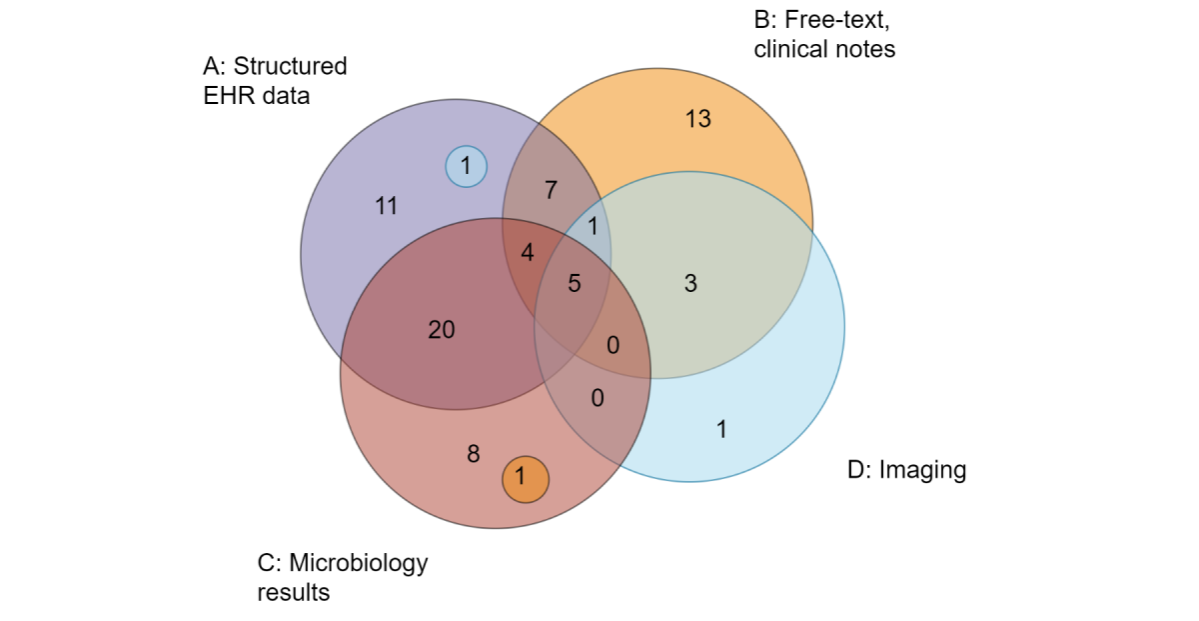
Venn diagram of the types of data used to identify bacterial infections in the included studies and guidelines. The data were divided into structured electronic health record data, free-text and clinical notes, microbiology results, and imaging. In total, 75 unique definitions were identified for different types of bacterial infections. EHR: electronic health record.

In the 147 included studies, 75 different methods and definitions were used to identify different types of bacterial infections. A total of 56% (42/75) used 2 or more datatypes to label, diagnose, or surveil infections, and 45% (34/75) required free-text and clinical notes as at least one of their data sources. In Table 1, the different types of data from the EHR needed to automatically detect patients with an infection are specified for each diagnostic guideline or infection definition used in the different prediction modeling studies or automated surveillance methods. Figure 5 shows the total number of methods used to identify patients with bacterial infections and the different data categories used. Most frequently (20/75, 27%), a combination of microbiology results and structured EHR data was used, followed by free-text (13/75, 17%) and structured EHR data (11/75, 15%). In total, 45% (34/75) of the identified methods used free text and clinical notes as one of their data sources.

For hospital-acquired infections (no specification of subtype), free-text information was needed for all definitions and methods, limiting the ability to detect patients with an infection based on structured EHR data. For pneumonia, some automated surveillance studies have identified patients without the need for free-text information [24,25], but they did include culture results in their definition. For SSIs and UTIs, a wide range of criteria were used compared with other types of infections. Abdominal surgery-related anastomotic leakage and abdominal infections were identified based on antibiotic treatment or surgical reinterventions supplemented with free-text data or imaging results. Bacterial culture data, in combination with structured EHR parameters, are used in most methods for detecting bloodstream infections. For *Clostridium difficile* infections, cerebral extraventricular and lumbar drain-related meningitis, methicillin-resistant *Staphylococcus aureus,* and prosthetic joint infection, the authors used a maximum of 2 criteria from different categories to define infection. Prediction modeling studies that did not use manual chart review for labeling patients in the data set relied on the registration of infections or the performance of surgical interventions, sometimes in combination with antibiotic administration [32].

When assessing infection severity according to the different Clavien-Dindo definitions, most (64%, 48/75) were based on identifying infections according to a Clavien-Dindo score of 1 or more. This indicates that, based on the registration of infection or clinical criteria only, infections were surveyed and predicted. In 23% of definitions (17/75), the prescription of antibiotic therapy or surgical intervention was included as the criterion, resulting in a Clavien-Dindo score of 2 or higher.

## Discussion

This scoping review assessed the methods and criteria used for identifying postoperative bacterial infections in prediction modeling and fully automated surveillance studies. We identified a total of 75 different methods and definitions from 147 included studies to identify patients with different types of bacterial infections. We found that 45% (34/75) used unstructured free-text and clinical notes as at least one of their data sources. Furthermore, out of 116 postoperative infection prediction studies, 108 (93%) used manual labeling based on self-defined criteria or diagnostic guidelines or used publicly available manually labeled databases. In addition, among the 31 automated surveillance studies, various methods, such as NLP, classification algorithms, and predefined criteria or rules on structured data, were used to automatically detect infections. Compared with manual chart review, automated surveillance systems have reported sensitivities for different types of infections ranging from 0.79 to 0.96, specificities from 0.81 to 0.96, PPVs from 0.31 to 0.76, and NPVs from 0.96 to 1.00. Finally, we found that different criteria were used among both prediction and surveillance studies to identify patients with infections, indicating that there is no uniform definition being used. Given the current use of different types of criteria and data used in prediction and surveillance studies, we were not able to identify or formulate a uniform and reliable method to automatically label patients with infections based on structured EHR data.

Prediction and surveillance of postoperative infections are crucial for early detection and assessment of the impact of preventative interventions but are currently hindered because the labeling of these cases is performed by resource-intensive manual chart review. In contrast to a previous study on semiautomated surveillance where high-risk patients were manually checked [13], we included only fully automated surveillance systems that were built to avoid requiring any human intervention. However, human intervention might still be required to incorporate the systems as well as to clean and preprocess the EHR data. Furthermore, we broadened the scope by assessing current labeling methods for prediction modeling studies, which, with some exceptions, were based on manual labeling according to established guidelines. In line with our findings, the predominant use of manual labeling was reported in a meta-analysis on the predictive performance of machine learning algorithms for SSI prediction [71]. Although manual labeling based on chart review is still the predominant method and is considered the reference standard, it must be noted that manual labeling may be flawed due to human errors and interobserver variability [9,10,12]. Furthermore, validating models only on national registries and databases limits the generalizability of developed prediction models and surveillance systems to other settings [72].

We extensively researched different definitions and methods from prediction modeling studies, guidelines, and surveillance studies to identify patients with bacterial infections that may occur after surgery and summarized different types of data needed to adhere to the different definitions. This study has several limitations. First, heterogeneity between studies (eg, differences in study design) prevented a meta-analysis, making it difficult to draw generalizable conclusions on optimal labeling methods. However, combining different types of studies allowed us to generate insight into the current methods of labeling and identifying patients with infections. Second, the distinction between structured and unstructured data may differ according to hospital data set and region (eg, microbiology results can be registered as free-text or tabular data). Despite these limitations, we could identify a lack of uniform definitions for labeling of postoperative infections exists, and that manual labeling is currently the predominant method. Third, pre-existing infections could have impacted the performance of surveillance algorithms and prediction models as well as label reliability [73]. This could explain the relatively lower PPVs and warrants further research before reliable implementation of automated surveillance systems.

Different types of data were used among the definitions and methods, including structured tabular data, microbiological data, free-text data, and imaging results. The importance of reliable, high-quality outcome data is essential for the reliable use of artificial intelligence and surveillance systems [74]. Using structured EHR data is preferable, as free text is often subject to misinterpretation and contains personal patient-specific data that conflict with privacy legislation and thus have restrictions on data use [75]. By extracting free-text information, NLP shows promise in uncovering postoperative infections from free-text data. However, challenges remain with respect to generalizability [76], transparency, reliability, and potential biases, including concerns about accuracy or unintended errors [77,78]. Furthermore, NLP methods can be computationally expensive, depend on the quality of the input data, and are influenced by nuances in language, dialects, and medical jargon. Considering that NLP methods can vary significantly in complexity, ranging from simple string searches to advanced neural networks, future research should investigate whether increased complexity leads to improved surveillance accuracy. The use of microbiology results in definitions is prevalent, despite their occasional unreliability due to the possibility of false negatives or positives, causing under- or overreporting of infections [13], and heterogeneous storage practices. This reliance on microbiology results could lead to errors or inconsistencies in infection identification.

Accurately identifying patients with infections based on an automated analysis of EHR data remains a challenge, and validation is difficult owing to the limitations of manual chart review, which until now has remained the reference standard for postoperative infections and other relevant patient outcomes. Manual labeling based on manual EHR chart review is unfeasible when scaling artificial intelligence–based or statistical prediction models to more than one hospital, with 100,000 patient records each. In some of the included studies, alternative approaches were identified that relied on treatments and other structured data sources [27,31-33,41-43]. For future prediction model development and surveillance, alternative approaches to identifying patients with infection should be explored, such as focusing on pharmacological and interventional treatments performed by clinicians, as these approaches are often stored in a structured format in the EHR system [27]. Emphasis should be placed on the consensus on the definition and whether it is worse to miss infections that do not require treatment compared with those that do. Compared with sensitivity, specificity, and NPV, automated surveillance systems have a lower PPV where heterogeneity is observed between the different types of infections. The PPV to detect pneumonia and SSIs is lower compared with other types of infections. This could be due to variations in clinical presentation, differences in diagnostic criteria, or the inherent complexity and variability of these particular infections. A lower PPV in general could be due to the use of low classification cutoffs to not miss any cases, but it could also indicate that the reference standard manual labeling may have resulted in erroneous labels and that the systems found infections where the human annotator did not [79]. In addition to detecting individual patients with infections, automated surveillance systems hold promise for assessing hospital incidence rates, predicting rates of complications, and evaluating the effectiveness of quality improvement initiatives, where the emphasis may shift from high PPVs to broader statistical insights.

In conclusion, there is currently no evidence to support fully automated labeling and identification of patients with infections based solely on structured EHR data. This is due to the diverse definitions of postoperative infection and the need for unstructured data types, such as free text and clinical notes, which were required as data sources in nearly half of the instances to assess an infection. Furthermore, manual labeling was still the predominant method in prediction modeling studies. Fully automatic surveillance methods may result in overreporting due to a relatively low PPV and heavy reliance on free-text data. Future research must focus on defining uniform or globally accepted definitions of postoperative infection that use criteria that can be extracted from the EHR, as well as prioritizing the development of more scalable automated methods for infection detection using EHR data.

## Appendix

Multimedia Appendix 1 Supplementary materials.

Multimedia Appendix 2 PRISMA-ScR checklist.

## Abbreviations

ACS NSQIP: American College of Surgeons National Surgical Quality Improvement Program
EHR: electronic health record
HAI: hospital-acquired Infections
NNIS: National Nosocomial Infections Surveillance System
NLP: natural language processing
NPV: negative predictive value
PPV: positive predictive value
PRISMA-ScR: Preferred Reporting Items for Systematic Reviews and Meta-Analyses Extension for Scoping Reviews
SSI: surgical site infection
SURPAS: Surgical Risk Preoperative Assessment System
UTI: urinary tract infection
