# Supplementary material for: Automated Identification of Postoperative Infections to Allow Prediction and Surveillance Based on Electronic Health Record Data: Scoping Review
Source: JMIR Med Inform. 2024 Sep 10;12:e57195. doi: 10.2196/57195 (PMC11422734; doi:10.2196/57195)
Supplement: Multimedia Appendix 1 [file medinform_v12i1e57195_app1.docx]

Automated identification of postoperative infections to allow prediction and surveillance based on EHR data: a Scoping Review
*Supplementary Material*

S.L. van der Meijden^1,2,*^, A.M. van Boekel^1^, H. van Goor^3^, R.G.H.H. Nelissen^4^, J.W. Schoones^5^, E.W. Steyerberg^6^, B.F. Geerts^2^, M.G.J. de Boer^7^, M.S. Arbous^1^

^1^Intensive Care Unit, Leiden University Medical Center, Leiden, The Netherlands

^2^Healthplus.ai B.V., Amsterdam, The Netherlands

^3^General surgery department, Radboud University Medical Center, Nijmegen, The Netherlands

^4^Department of Orthopedics, Leiden University Medical Center, Leiden, The Netherlands

^5^Directorate of Research Policy, Leiden University Medical Center, Leiden, The Netherlands

^6^Department of Biomedical Data Sciences, Leiden University Medical Center, Leiden, The Netherlands

^7^Department of infectious diseases, Leiden University medical Center, Leiden, The Netherlands

*Corresponding author details: [S.L.van_der_Meijden@lumc.nl](mailto:S.L.van_der_meijden@lumc.nl)

Contents

[Appendix A: Inclusion and exclusion criteria, and search queries 3](#_Toc171588270)

[A1: Inclusion and exclusion criteria 3](#_Toc171588271)

[A2: Search query prediction modelling studies 4](#_Toc171588272)

[A3: Search query automated surveillance studies 5](#_Toc171588273)

[Appendix B: Data extraction tables prediction models 7](#_Toc171588274)

[Appendix C: Data extraction from clinical guidelines and prediction models definitions 32](#_Toc171588275)

[Appendix D: Data extraction tables automated surveillance studies 51](#_Toc171588276)

[Appendix E: Definitions with further specified criteria 73](#_Toc171588277)

[Appendix F: Overview of included articles, guidelines and definitions 85](#_Toc171588278)

[Appendix G: Performance of surveillance algorithms 87](#_Toc171588279)

[References 89](#_Toc171588280)

#

# Appendix A: Inclusion and exclusion criteria, and search queries

## A1: Inclusion and exclusion criteria

**Table S1:** Inclusion and exclusion criteria per literature data source

| **Data source** | **Inclusion criteria** | **Exclusion criteria** |
| --- | --- | --- |
| Prediction models and biomarker studies | - Development and validation studies for prediction modelling and biomarker validation studies for all types of postoperative infections - Published in a peer-reviewed journal - Validated on retrospective or prospective patient data | - Studies on non-bacterial (e.g., viral) infections - Non-English studies (no English text available) - Wrong outcome studied, i.e., no postoperative infection - Infection time point not specified - No full-text available - Studies with a pediatric population (<18y/o) |
| Automated surveillance methods | - Studies that investigate the use of fully automated surveillance of hospital-acquired and/or postoperative infections based on electronic health records - Published in a peer-reviewed journal - Validated on retrospective or prospective patient data | - Studies not on surveillance of bacterial (e.g., viral) infections or hospital-acquired infections, excluding sexual transmitted diseases (STDs) and tuberculosis (TBC) as these are not deemed to be hospital-acquired - Non-English studies (no English text available) - No full-text available - Studies with a pediatric population (<18y/o) - Surveillance studies that require manual checking of the EHR as part of their algorithm - Studies that do not utilize hospital EHR data - Review studies and editorials - Study protocols - Non-human studies |

## A2: Search query prediction modelling studies

**PubMed query, queries were adjusted for other databases**

((("Prediction"[tw] OR "predict"[tw] OR "predictive"[tw] OR "predicting"[tw] OR "Predictive Value of Tests"[Mesh] OR "Forecasting"[mesh] OR "Forecast*"[tw] OR "Clinical Decision Rules"[Mesh] OR "Clinical Decision Rule"[tw] OR "Clinical Decision Rules"[tw] OR "Bayes Theorem"[Mesh] OR "bayes*"[tw]) AND ("Decision Support Techniques"[Mesh] OR "Decision Support Systems, Clinical"[Mesh] OR "Decision support"[tw] OR "Clinical Decision Rules"[Mesh] OR "Decision Rule"[tw] OR "Decision rules"[tw] OR "Risk stratification"[tw] OR "Risk Assessment"[Mesh] OR "Risk assessment"[tw] OR "Risk Management"[Mesh] OR "Risk evaluation"[tw] OR "Stratification"[tw] OR "Artificial Intelligence"[Mesh] OR "Artificial Intelligence"[tw] OR "Machine Learning"[mesh] OR "Machine learning"[tw] OR "Computational Intelligence"[tw] OR "Computer Reasoning"[tw] OR "Machine Intelligence"[tw] OR "Computer Simulation"[Mesh] OR "Computer model"[tw] OR "Computer models"[tw] OR "Computer model*"[tw] OR "Models, Statistical"[mesh] OR "Statistical Model"[tw] OR "Statistical Models"[tw] OR "Statistical Model*"[tw] OR "Binomial Models"[tw] OR "Binomial Model"[tw] OR "Binomial Model*"[tw] OR "Polynomial Models"[tw] OR "Polynomial Model"[tw] OR "Polynomial Model*"[tw] OR "Computer Simulation"[tw] OR "Computerized Models"[tw] OR "Computerised Models"[tw] OR "Computerized Model"[tw] OR "Computerised Model"[tw] OR "Computerized Model*"[tw] OR "Computerised Model*"[tw] OR "Neural Networks, Computer"[mesh] OR "Computational Neural Networks"[tw] OR "Computational Neural Network"[tw] OR "Computer Neural Networks"[tw] OR "Computer Neural Network"[tw] OR "Connectionist Models"[tw] OR "Connectionist Model"[tw] OR "Connectionist Model*"[tw] OR "Neural Network Models"[tw] OR "Neural Network Model"[tw] OR "Neural Network Model*"[tw] OR "Patient-Specific Modeling"[tw] OR "Patient-Specific Model"[tw] OR "Patient-Specific Models"[tw] OR "Patient-Specific Computational Modeling"[tw] OR "Patient-Specific Computational Models"[tw] OR "Patient-Specific Computational Model"[tw] OR "Models, Theoretical"[Mesh] OR "Mathematical model"[tw] OR "Mathematical models"[tw] OR "Mathematical model*"[tw] OR "Theoretical model"[tw] OR "Theoretical models"[tw] OR "Theoretical model*"[tw] OR "Mathematical Computing"[Mesh] OR "Mathematic Computing"[tw] OR "Mathematical Computing"[tw] OR "Statistical Computing"[tw] OR "Computer Based Statistical Programs"[tw] OR "Computer Based Statistical Program"[tw] OR "Computer Based Statistical Program*"[tw] OR "Computer Heuristics"[mesh] OR "Computer Heuristics"[tw] OR "Software"[mesh] OR "Computer Programs"[tw] OR "Computer Program"[tw] OR "Computer Software"[tw] OR "Decision Making, Computer-Assisted"[mesh] OR "Computer-Assisted Decision Making"[tw] OR "Computer-Assisted Medical Decision Making"[tw] OR "Computer-Assisted Clinical Decision Making"[tw] OR "Decision Model"[tw] OR "Decision Models"[tw] OR "Decision Modeling"[tw] OR "Decision Support Model"[tw] OR "Decision Support Models"[tw] OR "Decision Support Modeling"[tw] OR "Classification model"[tw] OR "Classification models"[tw] OR "Classification model*"[tw] OR "model"[ti] OR "models"[ti] OR "model*"[ti] OR "Algorithms"[Mesh] OR "Algorithms"[tw] OR "Algorithm"[tw] OR "Algorithms"[tw]) AND ("postoperative infection"[tw] OR "postoperative infections"[tw] OR "postoperative infection*"[tw] OR "post operative infection"[tw] OR "post operative infections"[tw] OR "post operative infect*"[tw] OR "postoperative wound infection"[tw] OR "postoperative wound infections"[tw] OR "postoperative wound infection*"[tw] OR "post operative wound infection"[tw] OR "post operative wound infections"[tw] OR "post operative wound infect*"[tw] OR "Surgical Wound Infection"[Mesh] OR "Surgical Wound Infection"[tw] OR "Surgical Wound Infections"[tw] OR "Surgical Site Infection"[tw] OR "Surgical Site Infections"[tw] OR (("Postoperative Complications"[Mesh] OR "Postoperative Period"[Mesh] OR "Postoperative Care"[Mesh] OR "postoperative"[tw] OR "post operative"[tw] OR "postoperativ*"[tw] OR "post operativ*"[tw] OR "postsurgery"[tw] OR "post surgery"[tw] OR "postsurgical"[tw] OR "post surgical"[tw] OR "postsurgical*"[tw] OR "post surgical*"[tw] OR "after surgery"[tw] OR "after operation"[tw] OR "after procedure"[tw] OR "post procedures"[tw] OR "post procedure"[tw] OR "post procedural"[tw] OR "postprocedure"[tw] OR "postprocedures"[tw] OR "postprocedural"[tw]) AND ("Infections"[Mesh] OR "Infections"[tw] OR "Infection"[tw] OR "Infect*"[tw] OR "pneumonia"[tw] OR "pneumon*"[tw] OR "Anastomotic Leak"[Mesh] OR "Anastomotic Leak"[tw] OR "Anastomotic Leaks"[tw] OR "Anastomotic Leaking"[tw] OR "sepsis"[tw] OR "Bacteremia"[tw] OR "Candidemia"[tw] OR "Endotoxemia"[tw] OR "Fungemia"[tw] OR "Septicemia"[tw] OR "Parasitemia"[tw] OR "Septic Shock"[tw] OR "Viremia"[tw] OR "Bacteraemia"[tw] OR "Candidaemia"[tw] OR "Endotoxaemia"[tw] OR "Fungaemia"[tw] OR "Septicaemia"[tw] OR "Parasitaemia"[tw] OR "Viraemia"[tw] OR "Bacteremi*"[tw] OR "Candidemi*"[tw] OR "Endotoxemi*"[tw] OR "Fungemi*"[tw] OR "Septicemi*"[tw] OR "Parasitemi*"[tw] OR "Viremi*"[tw] OR "Bacteraemi*"[tw] OR "Candidaemi*"[tw] OR "Endotoxaemi*"[tw] OR "Fungaemi*"[tw] OR "Septicaemi*"[tw] OR "Parasitaemi*"[tw] OR "Viraemi*"[tw] OR "Inflammation"[Mesh] OR "Inflammation"[tw] OR "Acute-Phase Reaction"[tw] OR "Foreign-Body Reaction"[tw] OR "Implant Capsular Contracture"[tw] OR "Seroma"[tw] OR "Serositis"[tw] OR "Suppuration"[tw] OR "Abscess"[tw] OR "Cellulitis"[tw] OR "Empyema"[tw] OR "Systemic Inflammatory Response Syndrome"[tw] OR "Cytokine Release Syndrome"[tw])))) OR (("prediction model"[tw] OR "prediction models"[tw] OR "prediction model*"[tw] OR "predictive model"[tw] OR "predictive models"[tw] OR "predictive model*"[tw]) AND ("postoperative infection"[tw] OR "postoperative infections"[tw] OR "postoperative infection*"[tw] OR "post operative infection"[tw] OR "post operative infections"[tw] OR "post operative infect*"[tw] OR "postoperative wound infection"[tw] OR "postoperative wound infections"[tw] OR "postoperative wound infection*"[tw] OR "post operative wound infection"[tw] OR "post operative wound infections"[tw] OR "post operative wound infect*"[tw] OR "Surgical Wound Infection"[Mesh] OR "Surgical Wound Infection"[tw] OR "Surgical Wound Infections"[tw] OR "Surgical Site Infection"[tw] OR "Surgical Site Infections"[tw] OR (("Postoperative Complications"[Mesh] OR "Postoperative Period"[Mesh] OR "Postoperative Care"[Mesh] OR "postoperative"[tw] OR "post operative"[tw] OR "postoperativ*"[tw] OR "post operativ*"[tw] OR "postsurgery"[tw] OR "post surgery"[tw] OR "postsurgical"[tw] OR "post surgical"[tw] OR "postsurgical*"[tw] OR "post surgical*"[tw] OR "after surgery"[tw] OR "after operation"[tw] OR "after procedure"[tw] OR "post procedures"[tw] OR "post procedure"[tw] OR "post procedural"[tw] OR "postprocedure"[tw] OR "postprocedures"[tw] OR "postprocedural"[tw]) AND ("Infections"[Mesh] OR "Infections"[tw] OR "Infection"[tw] OR "Infect*"[tw] OR "pneumonia"[tw] OR "pneumon*"[tw] OR "Anastomotic Leak"[Mesh] OR "Anastomotic Leak"[tw] OR "Anastomotic Leaks"[tw] OR "Anastomotic Leaking"[tw] OR "sepsis"[tw] OR "Bacteremia"[tw] OR "Candidemia"[tw] OR "Endotoxemia"[tw] OR "Fungemia"[tw] OR "Septicemia"[tw] OR "Parasitemia"[tw] OR "Septic Shock"[tw] OR "Viremia"[tw] OR "Bacteraemia"[tw] OR "Candidaemia"[tw] OR "Endotoxaemia"[tw] OR "Fungaemia"[tw] OR "Septicaemia"[tw] OR "Parasitaemia"[tw] OR "Viraemia"[tw] OR "Bacteremi*"[tw] OR "Candidemi*"[tw] OR "Endotoxemi*"[tw] OR "Fungemi*"[tw] OR "Septicemi*"[tw] OR "Parasitemi*"[tw] OR "Viremi*"[tw] OR "Bacteraemi*"[tw] OR "Candidaemi*"[tw] OR "Endotoxaemi*"[tw] OR "Fungaemi*"[tw] OR "Septicaemi*"[tw] OR "Parasitaemi*"[tw] OR "Viraemi*"[tw] OR "Inflammation"[Mesh] OR "Inflammation"[tw] OR "Acute-Phase Reaction"[tw] OR "Foreign-Body Reaction"[tw] OR "Implant Capsular Contracture"[tw] OR "Seroma"[tw] OR "Serositis"[tw] OR "Suppuration"[tw] OR "Abscess"[tw] OR "Cellulitis"[tw] OR "Empyema"[tw] OR "Systemic Inflammatory Response Syndrome"[tw] OR "Cytokine Release Syndrome"[tw])))))

## A3: Search query automated surveillance studies

**PubMed query, queries were adjusted for other databases**

(("fully automated surveillance"[tw] OR "real time surveillance"[tw] OR (("fully automated"[tw] OR "real time"[tw] OR "realtime"[tw]) AND ("surveillence"[tw] OR "surveill*"[tw] OR "detection"[tw] OR "detect*"[tw] OR "monitoring"[tw] OR "monitor*"[tw])) OR "electronic monitoring"[tw] OR "electronic monitor*"[tw] OR "automated monitoring"[tw] OR "automated monitor*"[tw] OR "real time monitoring"[tw] OR "real time monitor*"[tw] OR "realtime monitoring"[tw] OR "realtime monitor*"[tw] OR "electronic surveillance"[tw] OR "electronic surveill*"[tw] OR "automated surveillance"[tw] OR "automated surveill*"[tw] OR "real time surveillance"[tw] OR "real time surveill*"[tw] OR "realtime surveillance"[tw] OR "realtime surveill*"[tw] OR "electronic detection"[tw] OR "electronic detect*"[tw] OR "automated detection"[tw] OR "automated detect*"[tw] OR "real time detection"[tw] OR "real time detect*"[tw] OR "realtime detection"[tw] OR "realtime detect*"[tw] OR (("Data Mining"[mesh] OR "Algorithms"[mesh]) AND "Diagnosis, Computer-Assisted"[mesh])) AND ("Cross Infection"[mesh] OR "Surgical Wound Infection"[mesh] OR "Catheter-Related Infections"[mesh] OR "hospital infection"[tw] OR "nosocomial infection"[tw] OR "ward infection"[tw] OR "healthcare infection"[tw] OR "health-care infection"[tw] OR "blood-stream infection"[tw] OR "bloodstream infection"[tw] OR "surgical infection"[tw] OR "operative infection"[tw] OR "postoperative infection"[tw] OR "postsurgical infection"[tw] OR "post operative infection"[tw] OR "post surgical infection"[tw] OR "catheter infection"[tw] OR "ICU infection"[tw] OR "NICU infection"[tw] OR "intensive-care infection"[tw] OR "cross infection"[tw] OR "hospital infections"[tw] OR "nosocomial infections"[tw] OR "ward infections"[tw] OR "healthcare infections"[tw] OR "health-care infections"[tw] OR "blood-stream infections"[tw] OR "bloodstream infections"[tw] OR "surgical infections"[tw] OR "operative infections"[tw] OR "postoperative infections"[tw] OR "postsurgical infections"[tw] OR "post operative infections"[tw] OR "post surgical infections"[tw] OR "catheter infections"[tw] OR "ICU infections"[tw] OR "NICU infections"[tw] OR "PICU infections"[tw] OR "intensive-care infections"[tw] OR "cross infections"[tw] OR "hospital infect*"[tw] OR "nosocomial infect*"[tw] OR "ward infect*"[tw] OR "healthcare infect*"[tw] OR "health-care infect*"[tw] OR "blood-stream infect*"[tw] OR "bloodstream infect*"[tw] OR "surgical infect*"[tw] OR "operative infect*"[tw] OR "postoperative infect*"[tw] OR "postsurgical infect*"[tw] OR "post operative infect*"[tw] OR "post surgical infect*"[tw] OR "catheter infect*"[tw] OR "ICU infect*"[tw] OR "NICU infect*"[tw] OR "PICU infect*"[tw] OR "intensive-care infect*"[tw] OR "cross infect*"[tw] OR "hospital related infect*"[tw] OR "healthcare related infect*"[tw] OR "health care related infect*"[tw] OR "bloodstream related infect*"[tw] OR "surgical related infect*"[tw] OR "catheter related infect*"[tw] OR "icu related infect*"[tw] OR "hospital associated infect*"[tw] OR "nosocomial associated infect*"[tw] OR "healthcare associated infect*"[tw] OR "health care associated infect*"[tw] OR "bloodstream associated infect*"[tw] OR "catheter associated infect*"[tw] OR "ventilator associated infect*"[tw] OR "icu associated infect*"[tw] OR "hospital associated infect*"[tw] OR "nosocomial associated infect*"[tw] OR "healthcare associated infect*"[tw] OR "health care associated infect*"[tw] OR "bloodstream associated infect*"[tw] OR "catheter associated infect*"[tw] OR "ventilator associated infect*"[tw] OR "icu associated infect*"[tw] OR "Healthcare-associated infection"[tw] OR "Healthcare-associated infections"[tw] OR "Health care-associated infection"[tw] OR "Health care-associated infections"[tw] OR "Urinary Tract Infections"[Mesh] OR "Urinary Tract Infection"[tw] OR "Urinary Tract Infections"[tw] OR "Bacterial Infections"[mesh] OR "Infections"[majr:noexp] OR "infections"[ti] OR "infection"[ti] OR "infect*"[ti] OR "Systemic Inflammatory Response Syndrome"[mesh] OR "sepsis"[tw] OR "septic shock"[tw] OR "pneumonia"[tw] OR "nosocomial"[tw] OR "SIRS"[tw] OR "Systemic Inflammatory Response Syndrome"[tw] OR "hai"[tiab] OR "hais"[tiab] OR "hcai"[tiab] OR "hcais"[tiab] OR "ssi"[tiab] OR "vap"[tiab] OR "hap"[tiab] OR "vaps"[tiab] OR "haps"[tiab] OR "clabsi"[tiab]) AND ("Electronic Health Records"[mesh] OR "Medical Records Systems, Computerized"[mesh] OR "Computerised Medical Record"[tw] OR "Computerised Medical Records"[tw] OR "Computerized Medical Record"[tw] OR "Computerized Medical Records"[tw] OR "Electronic Health Record"[tw] OR "Electronic Health Records"[tw] OR "Electronic Medical Record"[tw] OR "Electronic Medical Records"[tw] OR "Electrical Medical Record"[tw] OR "Electrical Medical Records"[tw] OR "patient data management system"[tw] OR "patient data management systems"[tw] OR "hospital information system"[tw] OR "hospital information systems"[tw] OR (("Medical Records"[Mesh] OR "medical records"[tw] OR "clinical records"[tw] OR "patient records"[tw] OR "patients records"[tw] OR "patient's records"[tw] OR "patients' records"[tw]) AND ("Automation"[mesh] OR "electronics"[mesh] OR "Software"[mesh] OR "Computing Methodologies"[mesh] OR "Computer Systems"[mesh] OR "Electronic Data Processing"[mesh] OR "Computational Biology"[mesh] OR "Information Systems"[mesh] OR "Hospital Information Systems"[mesh] OR "Medical Informatics"[mesh] OR "Informatics"[mesh] OR "automat*"[tw] OR "computer*"[tw] OR "electronic*"[tw] OR "software*"[tw] OR "data-mining"[tw]))) NOT (("Infant"[mesh] OR "child"[mesh] OR "Adolescent"[mesh] OR "Infant"[ti] OR "child"[ti] OR "Adolescent"[ti] OR "Infants"[ti] OR "children"[ti] OR "Adolescents"[ti] OR "pediatr*"[ti] OR "paediatr*"[ti]) NOT ("Adult"[mesh] OR "adult"[ti] OR "middle aged"[ti] OR "elderly"[ti])))

# Appendix B: Data extraction tables prediction models

**Table S2:** Data extraction table - Definitions identified in a Systematic Review on post-surgical infection prediction models and biomarkers.

| **Study ID** | **Title** | **Author** | **2nd author if same first authors** | **Name tool** | **Type of tool** | **Type of infection** | **Infection definition used** | **Population** |
| --- | --- | --- | --- | --- | --- | --- | --- | --- |
| 1 | Applying the National Surgical Quality Improvement Program risk calculator to patients undergoing colorectal surgery: theory vs reality | Adegboyega 2017[1] |  | ACS NSQIP | Statistical | Pneumonia,Sepsis,SSI, all,UTI | ACS NSQIP | Gastro-intestinal |
| 2 | Validation of the ACS NSQIP surgical risk calculator for patients with early gastric cancer treated with laparoscopic gastrectomy | Alzahrani 2020[2] |  | ACS NSQIP | Statistical | Pneumonia,Sepsis,SSI, all,UTI | ACS NSQIP | Gastro-intestinal/oncology |
| 3 | Evaluating the ACS-NSQIP Risk Calculator in Primary GI Neuroendocrine Tumor: Results from the United States Neuroendocrine Tumor Study Group. | Armstrong 2019[3] |  | ACS NSQIP | Statistical | Pneumonia,Sepsis,SSI, all,UTI | ACS NSQIP | Gastro-intestinal |
| 4 | Development and validation of a multifactorial risk index for predicting postoperative pneumonia after major noncardiac surgery | Arozullah 2001[4] |  | Postoperative pneumonia risk index | Statistical model | Pneumonia | CDC | General (non-cardiac) |
| 5 | Assessing the predictive accuracy of the American College of Surgeons National Surgical Quality Improvement Project Surgical Risk Calculator in open ventral hernia repair. | Basta 2016[5] |  | ACS NSQIP | Statistical | Pneumonia,Sepsis,SSI, all,UTI | ACS NSQIP | Neurological/orthopedical |
| 6 | A predictive model of complications after spine surgery: the National Surgical Quality Improvement Program (NSQIP) 2005-2010. | Bekelis 2014[6] |  | ACS NSQIP | Statistical | Pneumonia,Sepsis,SSI, all,UTI | ACS NSQIP | Neurological |
| 7 | Evaluation of complications after laparoscopic and open appendectomy by the American College of Surgeons National Surgical Quality Improvement Program surgical risk calculator. | Benk 2022[7] |  | ACS NSQIP | Statistical model | Pneumonia,Sepsis,SSI, all,UTI | ACS NSQIP | Gastro-intestinal |
| 8 | Development and validation of a risk-stratification score for surgical site occurrence and surgical site infection after open ventral hernia repair | Berger 2013[8] |  | The Ventral Hernia Risk Score (VHRS) | Statistical model | All SSIs | CDC | Neurological |
| 9 | Failure of Colorectal Surgical Site Infection Predictive Models Applied to an Independent Dataset: Do They Add Value or Just Confusion? | Bergquist 2016[9] |  | NNIS, COLA, PREZIES, NSQIP | Statistical model | All SSIs | ACS NSQIP | Gastro-intestinal |
| 10 | Surgical Risk Is Not Linear: Derivation and Validation of a Novel, User-friendly, and Machine-learning-based Predictive OpTimal Trees in Emergency Surgery Risk (POTTER) Calculator. | Bertsimas 2018[10] |  | Predictive OpTimal Trees in Emergency Surgery Risk (POTTER) Calculator | AI model | Septic shock, all SSIs, sepsis, pneumonia, UTI | ASC NSQIP | Emergency surgery |
| 11 | Assessing the utility of deep neural networks in predicting postoperative surgical complications: a retrospective study | Bonde 2021[11] |  | N/A | Statistical model; AI model | All SSI, pneumonia, UTI, systemic sepsis, thrombophlebitis | ASC NSQIP | All |
| 12 | Revised Cardiac Risk Index versus ASA Status as a Predictor for Noncardiac Events After Posterior Lumbar Decompression. | Bronheim 2018[12] |  | Revised Cardiac Risk Index | Statistical model | All SSIs, pneumonia, UTI, sepsis, septic shock | ACS NSQIP | Orthopedic |
| 13 | Use of random forest machine learning algorithm to predict short term outcomes following posterior cervical decompression with instrumented fusion. | Cabrera 2023[13] |  | N/A | AI model | superficial incisional surgical site infection (SSI), deep incisional SSI, organ/space SSI, pneumonia, urinary tract infection, sepsis or septic shock | ACS NSQIP | Neurological |
| 14 | Validating a 3-point prediction rule for surgical site infection after coronary artery bypass surgery. | Chen 2010[14] |  | Australian Clinical Risk Index (ACRI) | Statistical model | All SSIs | CDC | Cardiothoracic |
| 15 | Development and Validation of a Nomogram Based on Geriatric Nutritional Risk Index to Predict Surgical Site Infection Among Gynecologic Oncology Patients. | Chen 2022[15] |  | N/A | Statistical model | All SSIs | CDC | Gynaecology |
| 16 | Preoperative Risk Factor Analysis and Dynamic Online Nomogram Development for Early Infections Following Primary Hip Arthroplasty in Geriatric Patients with Hip Fracture. | Cheng 2022[16] | Liu | N/A | Statistical model | SSI, UTI, Pneumonia | CDC, for UTI: UTI was diagnosed when patients were found to have positive urine bacterial culture results with at least one of the following symptoms: urinary frequency, urgency, hematuria, urinary retention, dysuria, suprapubic pain, or febrile reactions. | Orthopedic |
| 17 | Development of a Nomogram for Predicting Surgical Site Infection in Patients with Resected Lung Neoplasm Undergoing Minimally Invasive Surgery. | Cheng 2022[17] | Chen | N/A | Statistical model | All SSIs | ECDC | Oncology |
| 18 | Predicting deep surgical site infection in patients receiving open posterior instrumented thoracolumbar surgery--- A-DOUBLE-SSI risk score: a large retrospective multicenter cohort study in China. | Cheng 2023[18] | Liu | DOUBLE-SSI risk score | Statistical model; AI model | Deep or organ space SSI | CDC | Neurological |
| 19 | Development and Validation of a Nomograph Model for Post-Operative Central Nervous System Infection after Craniocerebral Surgery. | Cheng 2023[19] | Bai | N/A | Statistical model | Postoperative central nervous system infection | Clinical Diagnostic Criteria Patients with fever, intracranial hypertension, turbid or purulent cerebrospinal fluid (CSF), leukocytosis, glucose < 2.2 mmol/L, and CSF glucose content/serum glucose content ≤ 0.4. Etiological Diagnostic Criteria Patients with positive microbiological cultures of specimen smears, drainage tube tips, implants, and CSF on the basis of clinical diagnosis, excluding those with bacterial contamination and colonization. | Neurological |
| 20 | External validation of surgical risk preoperative assessment system (SURPAS) in pulmonary resection. | Chudgar 2020[20] |  | The Surgical Risk Preoperative Assessment System (SURPAS) | Statistical model | Infectious complication (all types, sepsis, UTI, wound disruption, superficial/deep/organ SSI) | ACS NSQIP | Cardiothoracic |
| 21 | Performance Comparison Between SURPAS and ACS NSQIP Surgical Risk Calculator in Pulmonary Resection. | Chudgar 2020[21] |  | The Surgical Risk Preoperative Assessment System (SURPAS) | Statistical model | Infectious complication (all types, sepsis, UTI, wound disruption, superficial/deep/organ SSI) | ACS NSQIP | Cardiothoracic |
| 22 | The DGAV risk calculator: development and validation of statistical models for a web-based instrument predicting complications of colorectal cancer surgery. | Crispin 2017[22] |  | DGAV risk calculator | Statistical model | SSI | SSI: surgical site infection requiring revision of the wound, anastomic leakage | Gastro-intestinal |
| 23 | Validation of a modified version of the national nosocomial infections surveillance system risk index for health services research. | Daneman 2009[23] |  | Modified version of the National Nosocomial Infections Surveillance System Risk Index | Statistical model | All SSIs (combined) | An SSI was defined by a hospital discharge diagnosis of SSI (during the index stay or readmission) or a physician claim for surgical wound infection (either inpatient or outpatient) within 30 days after the index procedure. | Elective surgery: abdominal, thoracic, vascular, cardiac, retroperitoneal, breast and skin, musculoskeletal, neurosurgical, head and neck, urogenital, ophtalmologic. |
| 24 | Evaluating the ACS NSQIP Risk Calculator in Primary Pancreatic Neuroendocrine Tumor: Results from the US Neuroendocrine Tumor Study Group. | Dave 2019[24] |  | ACS NSQIP | Statistical model | Pneumonia,Sepsis,SSI, all,UTI | ACS NSQIP | Gastro-intestinal |
| 25 | Predicted versus actual complications in Australian women undergoing post-mastectomy breast reconstruction: a retrospective cohort study using the BRA Score tool | Deek 2021[25] |  | BRA score | Statistical model | All SSIs | ACS NSQIP | Plastic surgery |
| 26 | Increased Surgical Site Subcutaneous Fat Thickness Is Associated with Infection after Posterior Cervical Fusion. | Donnally 2022[26] |  | N/A | Statistical model | SSI, all | CDC | Neurosurgery |
| 27 | Can the American College of Surgeons Risk Calculator Predict 30-Day Complications After Knee and Hip Arthroplasty? | Edelstein 2015[27] |  | ACS NSQIP | Statistical model | Pneumonia,SSI, all,UTI | ACS NSQIP | Orthopedic surgery |
| 28 | The efficacy of the American College of Surgeons Surgical Risk Calculator in the prediction of postoperative complications in oncogeriatric patients after curative surgery for abdominal tumors. | El Asmar 2022[28] |  | ACS NSQIP | Statistical model | Pneumonia,Sepsis,SSI, all,UTI | ACS NSQIP | Gastro-intestinal |
| 29 | Validation of the Artificial Intelligence-Based Predictive Optimal Trees in Emergency Surgery Risk (POTTER) Calculator in Emergency General Surgery and Emergency Laparotomy Patients. | El Hechi 2021[29] |  | Predictive OpTimal Trees in Emergency Surgery Risk (POTTER) Calculator | AI model | Septic shock, all SSIs, sepsis, pneumonia, UTI | ASC NSQIP | Emergency surgery |
| 30 | Artificial Intelligence versus Surgeon Gestalt in Predicting Risk of Emergency General Surgery. | El Moheb 2023[30] |  | Predictive OpTimal Trees in Emergency Surgery Risk (POTTER) Calculator | AI model | Septic shock, all SSIs, sepsis, pneumonia, UTI | ASC NSQIP | Emergency surgery |
| 31 | Ability to predict the development of surgical site infection in cardiac surgery using the Australian Clinical Risk Index versus the National Nosocomial Infections Surveillance-derived Risk Index | Figuerola-Tejerina 2017[31] |  | The Australian Clinical Risk Index (ACRI) | Statistical model | All SSIs | CDC | Cardiothoracic |
| 32 | Scoring system to predict the risk of surgical-site infection after colorectal resection | Gervaz 2012[32] |  | COLA score | Statistical model | All SSIs | CDC | Gastro-intestinal |
| 33 | Early Inflammatory Biomarkers as Predictive Factors for Freedom from Infection after Colorectal Cancer Surgery: A Prospective Cohort Study. | Goulart 2018[33] |  | N/A | Biomarker | SSI, all | CDC | Gastro-intestinal |
| 34 | Construct validation of machine learning in the prediction of short-term postoperative complications following total shoulder arthroplasty. | Gowd 2019[34] |  | Machine learning compared to other models KNN, 2) Logistic regression, 3) Random Forest, 4) Naive bayes, 5) Decision tree, 6) Gradient boosting trees, 7) ASA Classification, 8) Frailty index | AI model | All SSIs | ACS NSQIP | Orthopedical |
| 35 | Feasibility of Machine Learning in the Prediction of Short-Term Outcomes Following Anterior Cervical Discectomy and Fusion. | Gowd 2022[35] |  | N/A | AI model | Pneumonia,Sepsis,SSI, all,UTI | ACS NSQIP | Neurosurgery |
| 36 | Performance of surgical site infection risk prediction models in colorectal surgery: external validity assessment from three European national surveillance networks. | Grant 2019[36] |  | COLA (contamination class, obesity, laparoscopy, American Society of Anesthesiologists[ASA]) multivariable risk model, National Healthcare Safety Network (NHSN) multivariable risk model, newly constructed model | Statistical model | All SSIs | CDC and PHE SSISS | Gastro-intestinal |
| 37 | Development and validation of a risk calculator for predicting postoperative pneumonia. | Gupta 2013[37] |  | A risk calculator for predicting postoperative pneumonia | Statistical model | Pneumonia | ASC NSQIP | All specialties |
| 38 | Evaluation of the American College of Surgeons National Surgical Quality Improvement Program Risk Calculator to predict outcomes after hysterectomies. | Hamade 2022[38] |  | ACS NSQIP | Statistical model | All infections, all SSIs, pneumonia, sepsis, septic shock, UTI | ASC NSQIP | Gynaecology |
| 39 | Emergency Surgery Score Accurately Predicts the Risk of Post-Operative Infection in Emergency General Surgery | Han 2019[39] |  | The emergency surgery score (ESS) | Statistical model | All infections, all SSIs, pneumonia, sepsis, septic shock, UTI | ACS NSQIP | Gastro-intestinal |
| 40 | A Tool to Estimate Risk of 30-day Mortality and Complications After Hip Fracture Surgery: Accurate Enough for Some but Not All Purposes? A Study From the ACS-NSQIP Database. | Harris 2022[40] |  | N/A | Statistical model | All infections, all SSIs, pneumonia, sepsis, septic shock, UTI | ACS NSQIP | Trauma surgery |
| 41 | A method for estimating the risk of surgical site infection in patients with abdominal colorectal procedures. | Hedrick 2013[41] |  | Normogram | Statistical model | All SSIs | ASC NSQIP | Abdominal surgery |
| 42 | Refining the predictive variables in the "Surgical Risk Preoperative Assessment System" (SURPAS): a descriptive analysis. | Henderson 2019[42] |  | The Surgical Risk Preoperative Assessment System (SURPAS) | Statistical model | Infectious complication (all types, sepsis, UTI, wound disruption, superficial/deep/organ SSI) | ACS NSQIP | All specialties |
| 43 | Development of a nomogram to predict surgical site infection after closed comminuted calcaneal fracture. | Hu 2022[43] |  | N/A | Statistical model | All SSIs | CDC | Orthopedic |
| 44 | Surgical Predictive Model for Breast Cancer Patients Assessing Acute Postoperative Complications: The Breast Cancer Surgery Risk Calculator. | Jonczyk 2021[44] |  | The Breast Cancer Surgery Risk Calculator | Statistical model | All SSIs, UTI, pneumonia | ASC NSQIP | Oncology |
| 45 | Updating the predictive models for mortality and morbidity after low anterior resection based on the National Clinical Database. | Kawai 2023[45] |  | NCD-generated risk models | Statistical model | Anastomotic leakage, SSI all. | SSI: CDC, Anastomotic leakage was defined as the escape of bowel content from the drainage tube, requirement of drainage of extra-bowel bowel content, or apparent AL identified by imaging modalities. | Gastro-intestinal |
| 46 | Development and external validation of preoperative risk models for operative morbidities after total gastrectomy using a Japanese web-based nationwide registry. | Kikuchi 2017 [46] |  | Preoperative risk model for operative morbidities after total gastrectomy | Statistical model | All SSIs, pneumonia | ACS NSQIP | Gastro-intestinal |
| 47 | Derivation and validation of a clinical prediction rule for nosocomial pneumonia after coronary artery bypass graft surgery | Kinlin 2010 [47] |  | Clinical prediction rule | Statistical model | Pneumonia | Case managers recorded the occurrence of complications after CABG, including the development of nosocomial pneumonia. This outcome was defined on the basis of (1) new onset of pneumonia during current hospitalization, as documented by a treating physician; (2) positive results of sputum, blood, pleural, empyema, transtracheal, or transthoracic fluid cultures, compatible with the diagnosis and clinical findings of pneumonia; or (3) chest radiograph diagnostic of pulmonary infiltrates. Aspiration pneumonia was coded separately from nosocomial pneumonia and was not included in our primary analyses, because it was considered to be a unique clinical entity, with distinct pathophysiological processes, risk factors, and medical management strategies. | Cardiothoracic |
| 48 | External validity of the Society of Thoracic Surgeons risk stratification tool for deep sternal wound infection after cardiac surgery in a UK population | Kirmani 2013 [48] |  | The Society of Thoracic Surgeons (STS) risk score | Statistical model | Deep SSI | CDC | Cardiothoracic |
| 49 | Risk factors for surgical site infection in elective routine degenerative lumbar surgeries. | Klemencsics 2016 [49] |  | N/A | Statistical model | All SSIs | CDC | Neurological |
| 50 | Deep Learning-Based Computer-Aided Detection System for Preoperative Chest Radiographs to Predict Postoperative Pneumonia. | Lee 2023 [50] |  | N/A | Statistical model | SSI requiring hospital admission | CDC (requiring hospital admission) | Vascular surgery |
| 51 | Preoperative risk prediction of surgical site infection requiring hospitalization or reoperation in patients undergoing vascular surgery. | Leekha 2016 [51] |  | N/A | Statistical model | SSI requiring hospital admission | CDC (requiring hospital admission) | Vascular surgery |
| 52 | A nomogram prediction of postoperative surgical site infections in patients with perihilar cholangiocarcinoma. | Li 2017 [52] |  | N/A | Statistical model | All SSIs | CDC | Gastro-intestinal |
| 53 | Development and validation of a nomogram to predict the risk of surgical site infection within 1 month after transforaminal lumbar interbody fusion. | Lian 2023 [53] |  | N/A | Statistical model | All SSIs | CDC | Neurological |
| 54 | External validation of the ventral hernia risk score for prediction of surgical site infections. | Liang 2015 [54] |  | The Ventral Hernia Risk Score (VHRS) | Statistical model | All SSIs | CDC | Neurological |
| 55 | Training prediction models for individual risk assessment of postoperative complications after surgery for colorectal cancer. | Lin 2022 [55] |  | N/A | AI model | Clavien-dindo 3b or higher, and anastomotic leakage | Anastomotic leakage was defned as a either type A, B or C leakage diagnosed clinically, radiologically, endoscopically or surgically | Gastro-intestinal |
| 56 | Using Preoperative and Intraoperative Factors to Predict the Risk of Surgical Site Infections After Lumbar Spinal Surgery: A Machine Learning-Based Study. | Liu 2022 [56] | Ying | N/A | AI model | All SSIs | CDC | Neurological |
| 57 | Machine Learning-based Correlation Study between Perioperative Immunonutritional Index and Postoperative Anastomotic Leakage in Patients with Gastric Cancer. | Liu 2022 [57] | Lei | N/A | Statistical model, AI model, Biomarker | Anastomotic leakage | N/A | Gastro-intestinal, cardiothoracic, neurosurgery |
| 58 | Accuracy of American College of Surgeons National Surgical Quality Improvement Program Universal Surgical Risk Calculator in Predicting Complications Following Robot-Assisted Radical Cystectomy at a National Comprehensive Cancer Center. | Lone 2019 [58] |  | ACS NSQIP | Statistical | Pneumonia,Sepsis,SSI, all,UTI | ACS NSQIP | Urological |
| 59 | Evaluation of the Performance of ACS NSQIP Surgical Risk Calculator in Emergency General Surgery Patients. | Long 2020 [59] |  | ACS NSQIP | Statistical | Pneumonia,Sepsis,SSI, all,UTI | ACS NSQIP | Emergency surgery |
| 60 | Machine-learning models for predicting surgical site infections using patient pre-operative risk and surgical procedure factors. | Mamlook 2023 [60] |  | N/A | AI model | All SSIs | ACS NSQIP | All specialties |
| 61 | Predicting complications following radical cystectomy with the ACS NSQIP universal surgical risk calculator. | Mannas 2020 [61] |  | ACS NSQIP | Statistical | Pneumonia,Sepsis,SSI, all,UTI | ACS NSQIP | Urological |
| 62 | An Evaluation of the Utility of the Breast Reconstruction Risk Assessment Score Risk Model in Prepectoral Tissue Expander Breast Reconstruction. | Martin 2020 [62] |  | The breast reconstruction risk assessment score (BRAscore) | Statistical model | All SSIs | SSI requiring IV antibiotics or hospital admission, seroma requiring drainage, incision dehiscence and explanation | Plastic surgery |
| 63 | Predictors of infection after pulmonary endarterectomy for chronic thrombo-embolic pulmonary hypertension. | Maruna 2011 [63] |  | N/A | Biomarker | Infection, all | CDC | Cardiothoracic surgery |
| 64 | Validation of the AI-based Predictive OpTimal Trees in Emergency Surgery Risk (POTTER) Calculator in Patients 65 Years and Older. | Maurer 2020 [64] |  | Predictive OpTimal Trees in Emergency Surgery Risk (POTTER) Calculator | AI model | Septic shock, all SSIs, sepsis, pneumonia, UTI | ASC NSQIP | Emergency surgery |
| 65 | Can the American College of Surgeons Risk Calculator Predict 30-Day Complications After Cervical Spine Surgery? | McCarthy 2019 [65] |  | ACS NSQIP | Statistical | Pneumonia,Sepsis,SSI, all,UTI | ACS NSQIP | Neurological |
| 66 | Development of a Risk Score to Predict Anastomotic Leak After Left-Sided Colectomy: Which Patients Warrant Diversion? | McKenna 2020 [66] |  | N/A | Statistical model | Anastomotic leakage | ACS NSQIP: Anastomotic leak within 30 days of surgery that required treatment with antibiotics, percutaneous drainage, or reoperation. | Gastro-intestinal |
| 67 | Surgical Risk Preoperative Assessment System (SURPAS): II. Parsimonious Risk Models for Postoperative Adverse Outcomes Addressing Need for Laboratory Variables and Surgeon Specialty-specific Models. | Meguid 2016 [67] |  | The Surgical Risk Preoperative Assessment System (SURPAS) | Statistical model | Infectious complication (all types, sepsis, UTI, wound disruption, superficial/deep/organ SSI) | ACS NSQIP | All specialties |
| 68 | Optimizing classical risk scores to predict complications in head and neck surgery: a new approach | Menezes 2021 [68] |  | ASA, P-POSSUM, ACS-NSQIP, ARISCAT | Statistical model | Pneumonia,SSI, all | ACS NSQIP | Head/neck |
| 69 | Do risk calculators accurately predict surgical site occurrences? | Mitchell 2016 [69] |  | ASC-NISQIP risk calculator Ventral hernia risk score (VHRS) Ventral hernia working group (VHWG) Centers for disease control and prevention wound class Hernia wound risk assessment tool (HW-RAT) | Statistical model | All SSIs | CDC | Neurosurgical/ |
| 70 | Risk scores as useful predictors of perioperative complications in patients with rectal cancer who received radical surgery. | Miyakita 2017 [70] |  | Comparison of 5 models: 1) E-PASS CRS (Estimation of Physiologic Ability and Surgical Stress Comprehensive Risk Score) 2) SAS (Surgical Apgar Score) 3) PNI (Prognostic Nutritional Index) 4) CR-POSSUM (Colorectal POSSUM) 5) NLR (neutrophil-to-lymfocyte ratio) | Statistical model, biomarker | Infectious complications, anastomotic leakage | Infectious complications (wound infection, inflammation of the pelvic dead space, and intraabdominal abscess), anastomotic leakage. The diagnosis of anastomotic leakage was based on the properties of drainage fluid or radiographic findings. anastomotic leakage of Clavien-Dindo grade 3b or higher that required reoperation were defined as complication. All complications, infectious complications, and intestinal obstruction of Clavien-Dindo grade 3a or higher that required surgical intervention and anastomotic leakage of Clavien-Dindo grade 3b or higher that required reoperation were defined as complications. | Gastro-intestinal |
| 71 | C-Reactive Protein and Procalcitonin as Early Markers of Septic Complications after Laparoscopic Sleeve Gastrectomy in Morbidly Obese Patients Within an Enhanced Recovery After Surgery Program. | Muñoz 2016 [71] |  | N/A | Biomarker | Septic complications | CDC | Bariatric surgery |
| 72 | Does the ACS NSQIP Surgical Risk Calculator Accurately Predict Complications Rates After Anterior Lumbar Interbody Fusion Procedures? | Narain 2020 [72] |  | ACS NSQIP | Statistical model | Pneumonia,SSI, all,UTI | ACS NSQIP | Orthopedic surgery |
| 73 | Multivariable predictors of postoperative surgical site infection after general and vascular surgery: results from the patient safety in surgery study. | Neumayer 2007 [73] |  | N/A | Statistical model | All SSIs | ACS NSQIP | General and vascular surgery |
| 74 | Development and validation of machine learning models to predict gastrointestinal leak and venous thromboembolism after weight loss surgery: an analysis of the MBSAQIP database. | Nudel 2021 [74] |  | ANN, XGB, LR | AI model | anastomotic leakage | Leak was defined as postoperative organ space infection, presence of a surgical drain for more than 30 days, or leak as the suspected reason for any readmission, reintervention, or reoperation | Gastro-intestinal |
| 75 | Development of a model predicting the risk of eight major postoperative complications after esophagectomy based on 10 826 cases in the Japan National Clinical Database. | Ohkura 2019 [75] |  | N/A | Statistical model | All SSIs, pneumonia, sepsis, anastomotic leakage | ACS NSQIP | Gastro-intestinal |
| 76 | Preliminary Evaluation of a Novel Artificial Intelligence-based Prediction Model for Surgical Site Infection in Colon Cancer. | Ohno 2022 [76] |  | N/A | AI model | SSI, all | CDC | Gastro-intestinal |
| 77 | Performance of a novel risk model for deep sternal wound infection after coronary artery bypass grafting. | Orlandi 2022 [77] |  | REPINF compared to STS model | Statistical model | Deep sternal wound infection | CDC | Cardiothoracic |
| 78 | A novel, validated risk score to predict surgical site infection after pancreaticoduodenectomy. | Poruk 2016 [78] |  | N/A | Statistical model | All SSIs | CDC | Gastro-intestinal |
| 79 | Creation of a novel risk score for surgical site infection and occurrence after ventral hernia repair | Poruk 2016 [79] |  | HVHR SSI Risk score | Statistical model | All SSIs | CDC | Neurological |
| 80 | ACS NSQIP Risk Calculator: An Accurate Predictor of Complications in Major Head and Neck Surgery? | Prasad 2016 [80] |  | ACS NSQIP | Statistical model | Pneumonia,Sepsis,SSI, all,UTI | ACS NSQIP | Head/neck |
| 81 | Assessment of the American College of Surgeons National Surgical Quality Improvement Program Calculator in Predicting Outcomes and Length of Stay After Ivor Lewis Esophagectomy: A Single-Center Experience | Ravindran 2020 [81] |  | ACS NSQIP | Statistical model | Pneumonia,Sepsis,SSI, all,UTI | ACS NSQIP | Gastro-intestinal |
| 82 | Performance of a Machine Learning Algorithm Using Electronic Health Record Data to Predict Postoperative Complications and Report on a Mobile Platform. | Ren 2022 [82] |  | MySurgeryRisk | AI model | Sepsis, wound complications. | N/A | All specialties |
| 83 | Predictors of Anastomotic Leak in Elderly Patients After Colectomy: Nomogram-Based Assessment From the American College of Surgeons National Surgical Quality Program Procedure-Targeted Cohort | Rencuzogullari 2017 [83] |  | N/A | Statistical model | Anastomotic leakage | ASC NSQIP | Gastro-intestinal |
| 84 | Evaluation of the performance of the ACS NSQIP surgical risk calculator in gynecologic oncology patients undergoing laparotomy. | Rivard 2016 [84] |  | ACS NSQIP | Statistical model | Pneumonia,Sepsis,SSI, all,UTI | ACS NSQIP | Gynaecology |
| 85 | Risk adjustment performance between NNIS index and NHSN model for postoperative colorectal surgical site infection: A retrospective cohort study. | Sangsuwan 2022 [85] |  | NNIS risk index and NHSN procedure-specific risk model | Statistical model | All SSI | CDC | Gastro-intestinal |
| 86 | C-reactive protein as an early predictor for anastomotic leakage in elective abdominal surgery. | Scepanovic 2013 [86] |  | N/A | Biomarker | Infection, all | CDC | Gastro-intestinal |
| 87 | Ability of the National Surgical Quality Improvement Program Risk Calculator to Predict Complications Following Total Laryngectomy | Schneider 2016 [87] |  | ACS NSQIP | Statistical model | Pneumonia,Sepsis,SSI, all,UTI | ACS NSQIP | Head/neck |
| 88 | Is the ACS-NSQIP Risk Calculator Accurate in Predicting Adverse Postoperative Outcomes in the Emergency Setting? An Italian Single-center Preliminary Study. | Scotton 2020 [88] |  | ACS NSQIP | Statistical model | Pneumonia,Sepsis,SSI, all,UTI | ACS NSQIP | Emergency surgery |
| 89 | Can the American College of Surgeons NSQIP Surgical Risk Calculator Accurately Predict Adverse Postoperative Outcomes in Emergency Abdominal Surgery? An Italian Multicenter Analysis. | Scotton 2023 [89] |  | ACS NSQIP | Statistical model | Pneumonia,Sepsis,SSI, all,UTI | ACS NSQIP | Emergency surgery |
| 90 | Early diagnosis of anastomotic leakage after colorectal cancer surgery using an inflammatory factors-based score system. | Shi 2022 [90] |  | N/A | Biomarker, Statistical model | Anastomotic leakage | AL was defined as defect anastomosis; manifested radiological changes after surgery (with or without clinical intervention); colour turbidity, faecal, or other indicative changes observed from in the drain fluid; the peri-anastomotic abscess and angiogenic intra-abdominal infection was also considered as AL. | Gastro-intestinal |
| 91 | Diagnostic and predictive value of the silkworm larvae plasma test for postoperative infection following gastrointestinal surgery. | Shimizu 2005 [91] |  | N/A | Biomarker | Infection, all | CDC | Gastro-intestinal |
| 92 | Body fat composition assessment using analytic morphomics predicts infectious complications after bowel resection in Crohn's disease. | Stidham 2015 [92] |  | Body Fat Composition Assessment Using Analytic Morphomics | Statistical model | Abdominal infection | The composite endpoint of postoperative infectious complication included the following criteria: (1) Use of intravenous antibiotics for an intra-abdominal infection source for at least 10 days, (2) Postoperative abdominal drain placement within 30 days of index surgery, and (3) reoperation within 30 days of index surgery. Medical records were reviewed to determine the indications of the interventions used in our composite endpoint. Infectious complications potentially related to perioperative interventions, but remote from the surgical site, including Clostridium difficile colitis, urinary tract infections, and pneumonia, were not included in the composite postoperative complication endpoint | Gastro-intestinal |
| 93 | Frailty Is Superior to Age for Predicting Readmission, Prolonged Length of Stay, and Wound Infection in Elective Otology Procedures. | Stidham 2022 [93] |  | N/A | Statistical model | All SSI | ACS NSQIP | Head/neck |
| 94 | Evaluation of the National Surgical Quality Improvement Program Universal Surgical Risk Calculator for a gynecologic oncology service. | Szender 2015 [94] |  | ACS NSQIP | Statistical model | Pneumonia,Sepsis,SSI, all,UTI | ACS NSQIP | Gynaecology |
| 95 | Risk-adjustment models in patients undergoing head and neck surgery with reconstruction. | Tam 2020 [95] |  | N/A | Statistical model | Pneumonia, superficial SSI | ACS NSQIP | Head/neck surgery |
| 96 | Predictive value of the ACS NSQIP calculator for head and neck reconstruction free tissue transfer. | Tierney 2020 [96] |  | ACS NSQIP | Statistical model | Pneumonia, superficial SSI | ACS NSQIP | Head/neck surgery |
| 97 | The Value of Aggregated High-Resolution Intraoperative Data for Predicting Post-Surgical Infectious Complications at Two Independent Sites. | Tourani 2019 [97] |  | N/A | Statistical model | All SSIs, UTI, pneumonia, sepsis, septic shock | ACS NSQIP | All specialties |
| 98 | How Accurate Are the Surgical Risk Preoperative Assessment System (SURPAS) Universal Calculators in Total Joint Arthroplasty? | Trickey 2020 [98] |  | The Surgical Risk Preoperative Assessment System (SURPAS) | Statistical model | Infectious complication (all types, sepsis, UTI, wound disruption, superficial/deep/organ SSI) | ACS NSQIP | Orthopedic |
| 99 | Validation of the ACS NSQIP surgical risk calculator in older patients with colorectal cancer undergoing elective surgery. | vanderHulst 2022[99] |  | ACS NSQIP | Statistical model | All SSIs, UTI, pneumonia, sepsis, septic shock | ACS NSQIP | Gastro-intestinal |
| 100 | Conventional regression analysis and machine learning in prediction of anastomotic leakage and pulmonary complications after esophagogastric cancer surgery. | vanKooten 2022 [100] |  | N/A | AI model, statistical model | Anastomotic leakage | Anastomotic leakage was defined as any clinically or radiologically proven anastomotic leakage. | Gastro-intestinal |
| 101 | Serum Procalcitonin as a Valuable Diagnostic Tool in the Early Detection of Infectious Complications after Open Abdominal Aortic Repair. | Varetto 2016 [101] |  | N/A | Biomarker | Infection, all | CDC | Vascular surgery |
| 102 | Predictive performance of the American College of Surgeons universal risk calculator in neurosurgical patients. | Vaziri 2018 [102] |  | ACS NSQIP | Statistical model | Pneumonia,Sepsis,SSI, all,UTI | ACS NSQIP | Neurological |
| 103 | The Not-So-Distant Future or Just Hype? Utilizing Machine Learning to Predict 30-Day Post-Operative Complications in Laparoscopic Colectomy Patients. | Velmahos 2023 [103] |  | N/A | AI model | Organ/Space SSI, Sepsis, Superficial SSI, septic shock | ACS NSQIP | All specialties |
| 104 | Predicting complications of major head and neck oncological surgery: an evaluation of the ACS NSQIP surgical risk calculator | Vosler 2018 [104] |  | ACS NSQIP | Statistical model | Pneumonia,SSI, all,UTI | ACS NSQIP | Head/neck |
| 105 | Development and Internal Validation of the Abdominoplasty Risk Calculator. | Vu 2018 [105] |  | Abdominoplasty Risk Calculator | Statistical model | Superficial SSI, deep or organ-spaced infection, sepsis/septic shock, UTI, pneumonia | ASC NSQIP | Plastic surgery |
| 106 | Leveraging electronic health records for predictive modeling of post-surgical complications. | Weller 2018 [106] |  | N/A | AI model | All SSIs | SSI was defined as any case of opening a wound or use antibiotics | Gastro-intestinal |
| 107 | Evaluation of the ACS NSQIP surgical risk calculator in patients undergoing pelvic organ prolapse surgery | Wherley 2020 [107] |  | ACS NSQIP | Statistical model | Pneumonia,Sepsis,SSI, all,UTI | ACS NSQIP | Gynaecology |
| 108 | The ACS NSQIP Risk Calculator Is a Fair Predictor of Acute Periprosthetic Joint Infection. | Wingert 2016 [108] |  | ACS NSQIP | Statistical model | Pneumonia,Sepsis,SSI, all,UTI | ACS NSQIP | Orthopedic |
| 109 | Predicting Complications Following Robot-Assisted Partial Nephrectomy with the ACS NSQIP(®) Universal Surgical Risk Calculator. | Winoker 2017 [109] |  | ACS NSQIP | Statistical model | Pneumonia,Sepsis,SSI, all,UTI | ACS NSQIP | Urological |
| 110 | A preoperative nomogram for sepsis in percutaneous nephrolithotomy treating solitary, unilateral and proximal ureteral stones. | Xun 2020 [110] |  | Normogram | Statistical | Sepsis or septic shock | ACCP/SCCM | Urological |
| 111 | Validation of the American College of Surgeons Risk Calculator for preoperative risk stratification. | Yap 2018 [111] |  | ACS NSQIP and Revised Cardiac Risk Index (RCRI) | Statistical model | Pneumonia, all SSI, UTI | ACS NSQIP | All specialties |
| 112 | Using multiple indicators to predict the risk of surgical site infection after ORIF of tibia fractures: a machine learning based study. | Ying 2023 [112] |  | N/A | AI model | SSI | CDC | Emergency surgery |
| 113 | Risk assessment of morbidities after right hemicolectomy based on the National Clinical Database in Japan. | Yoshida 2018 [113] |  | NCD-generated risk models | Statistical model | All SSI, systemic sepsis (all sepsis), pneumonia, anastomotical leakage | CDC | Gastro-intestinal |
| 114 | The Significance of Interleukin-6 in the Early Detection of Surgical Site Infections after Definitive Operation for Gastrointestinal Fistulae. | Zhang 2018 [114] |  | N/A | Biomarker | All SSI | CDC | Gastro-intestinal |
| 115 | Identification of risk factors for infection after mitral valve surgery through machine learning approaches. | Zhang 2023 [115] |  | N/A | AI model | All SSIs, and infections occurring at other sites (e.g., pneumonia; cardiac device infection; urinary tract infection; mediastinitis; empyema; endocarditis; infectious myocarditis or pericarditis; Clostridium difficile colitis, and bloodstream infections) | N/A | Cardiothoracic surgery |
| 116 | Modified Frailty Index Combined with a Prognostic Nutritional Index for Predicting Postoperative Complications of Hip Fracture Surgery in Elderly. | Zhou 2022 [116] |  | Modified frailty index combined with prognostic nutritonal index | Statistical model | Surgical site infection, UTI, pneumonia | N/A | Trauma surgery |

# Appendix C: Data extraction from clinical guidelines and prediction models definitions

**Table S3:** Infection definitions for surgical site infections (SSI) and abdominal infections. Per definition and per criterion in the definition, it is indicated whether it is available from structured EHR data (tabular variables such as laboratory results, vital signs), available from free text (e.g., redness), or available from other sources (e.g., imaging).

| **Study or guideline or surveillance method** | **Type of SSI** | **Timeframe** | **Definition used for (diagnosis, prediction, surveillance)** | **Definition in words** | **Criteria available from structured data** | **Clinical criterion (could be available from unstructured (text) data)** | **Criteria available from other sources (e.g., imaging)** |
| --- | --- | --- | --- | --- | --- | --- | --- |
| CDC / ACS NSQIP | Superficial** | < 30 days | Diagnosis | Involves only skin and subcutaneous tissue of the incision  **AND**  patient has at least **one** of the following (a-d):  a. purulent drainage from the superficial incision.   b. organism(s) identified from an aseptically-obtained specimen from the superficial incision or subcutaneous tissue by a culture or nonculture based microbiologic testing method which is performed for purposes of clinical diagnosis or treatment (for example, not Active Surveillance Culture/Testing (ASC/AST)).   c. superficial incision that is deliberately opened by a surgeon, physician* or physician designee and culture or non-culture based testing of the superficial incision or subcutaneous tissue is not performed  **AND**  patient has at least one of the following signs or symptoms:  1) localized pain or tenderness;  2) localized swelling; erythema; or  3) heat.   d. diagnosis of a superficial incisional SSI by a physician* or physician designee. | X | X    X  X  X X X  X |  |
| WHO | Superficial |  | Diagnosis | Purulent drainage (pus) from superficial incision   **OR** organism identified (if culture done)****  **OR** superficial incision deliberately re-opened   **AND** infection symptoms:  - Drainage of fluid from wound: pus versus clear(serous)/bloody/other  - Pain/tenderness beyond normal for operation  - Localized swelling or wound breakdown  - Redness/heat of skin  - Generally unwell, especially fever >38 C   **OR** Surgeon/attending physician diagnosis | X  X | X    X   X  X X X X  X | X |
| CDC / ACS NSQIP | Deep | < 30 or  < 90 days | Diagnosis | Involves deep soft tissues of the incision (for example, fascial and muscle layers)   **AND** patient has at least one of the following:  a. purulent drainage from the deep incision.  b. a deep incision that spontaneously dehisces, or is deliberately opened or aspirated by a surgeon, physician* or physician designee  **AND** organism(s) identified from the deep soft tissues of the incision by a culture or non-culture based microbiologic testing method which is performed for purposes of clinical diagnosis or treatment (for example, not Active Surveillance Culture/Testing (ASC/AST)) or culture or nonculture based microbiologic testing method is not performed. A culture or non-culture based test from the deep soft tissues of the incision that has a negative finding does not meet this criterion.   **AND** patient has at least one of the following signs or symptoms: 1) fever (>38°C);  2) localized pain or tenderness.  2) an abscess or other evidence of infection involving the deep incision that is detected on gross anatomical or histopathologic exam, or imaging test. | X (if registered as procedure)            X | X    X X             X X X | X |
| WHO | Deep |  | Diagnosis | Purulent drainage (pus) from deep incision   **OR** Deep incision dehiscence or deliberately opened by surgeon   **AND** organism identified (if culture done)****   **AND** infection symptoms: (See WHO superficial)   **OR** Deep infection/abcess found on imaging/examination | X (if registered as procedure) | X  X    X  X | X    X |
| CDC / ACS NSQIP | Organ space | < 30 days or < 90 days | Diagnosis | Involves any part of the body deeper than the fascial/muscle layers that is opened or manipulated during the operative procedure   **AND** patient has at least one of the following:  a. purulent drainage from a drain that is placed into the organ/space (for example, closed suction drainage system, open drain, T-tube drain, CTguided drainage).   b. organism(s) identified from fluid or tissue in the organ/space by a culture or non-culture based microbiologic testing method which is performed for purposes of clinical diagnosis or treatment (for example, not Active Surveillance Culture/Testing (ASC/AST)).   c. an abscess or other evidence of infection involving the organ/space that is detected on gross anatomical or histopathologic exam, or imaging test evidence suggestive of infection.   **AND** meets at least one criterion for a specific organ/space infection site***. |  | X    X  X | X  X |
| WHO | Organ space |  | Diagnosis | Purulent drainage (pus) from sterile organ or space (from an inserted drain)   **OR** Organ or space infection/abcess found on imaging/examination   **OR** Organism identified from fluid/tissue from organ/space**** |  | X   X | X |
| Study Group of Rectal Cancer AL definition | Anastomotic leakage |  | Diagnosis | A defect of the intestinal wall at the anastomotic site, which leads to a communication between the intra- and extraluminal compartments, or as an abscess adjacent to the anastomosis.  A computed tomographic (CT) scan with rectal contrast was performed in patients with suspected AL in the absence of unquestionable clinical signs of peritonitis, which would indicate urgent surgery.  On postoperative days 3, 5, and 7, the C-reactive protein level was determined in all cases. For patients with a Creactive protein level >200 mg/l, a CT scan was performed.  The following CT findings were considered suggestive of anastomotic failure: Contrast leakage from inside the bowel to the pelvis or abdominal cavity, abscess, or perianastomotic collection associated with or without localized pneumoperitoneum. | X | x | X  x |
| Daneman et al. | All SSIs combined | 30 days | Prediction | An SSI was defined by a hospital discharge diagnosis of SSI (during the index stay or readmission)   **OR** a physician claim for surgical wound infection (either inpatient or outpatient) within 30 days after the index procedure. | X  X |  |  |
| Stidham et al. | Abdominal infection |  | Prediction | The composite endpoint of postoperative infectious complication included the following criteria*****:   1) Use of intravenous antibiotics for an intra-abdominal infection source for at least 10 days,   2) Postoperative abdominal drain placement within 30 days of index surgery, and   3) reoperation within 30 days of index surgery. | X   X   X | X |  |
| Miyakita et al. | Infectious complications, anastomotic leakage |  | Prediction | The diagnosis of anastomotic leakage was based on the properties of drainage fluid or radiographic findings. anastomotic leakage of Clavien-Dindo grade 3b or higher that required reoperation were defined as complication.   All complications, infectious complications, and intestinal obstruction of Clavien-Dindo grade 3a or higher that required surgical intervention and anastomotic leakage of Clavien-Dindo grade 3b or higher that required reoperation were defined as complications. | X  X | X     X | X  X |
| Weller et al. | SSIs (all types) |  | Prediction | SSI was defined as any case of opening a wound or use antibiotics | X | X |  |
| Crispin et al. | SSI | 30 days | Prediction | Requiring revision of the wound | X |  |  |
| McKenna et al. | Anastomotic leakage | 30 days | Prediction | Required treatment with antibiotics  **OR** percutaneous drainage  **OR** reoperation | X  X  x | x |  |
| Nudel et al. | Anastomotic leakage | 30 days | Prediction | Postoperative organ space infection  **OR** presence of a surgical drain for more than 30 days  **OR** leak as the suspected reason for any readmission, intervention or reoperation | x | X  X  X |  |
| Kawai et al. | Anatomotic leakage | 30 days | Prediction | Anastomotic leakage was defined as the escape of bowel content from the drainage tube, requirement of drainage of extra-bowel bowel content, or apparent AL identified by imaging modalities. Only Clavien-Dindo II or higher. |  | x | x |
| Lin et al. | Anatomotic leakage | 30 days | Prediction | Anastomotic leakage was defined  as a either type A, B or C leakage diagnosed clinically,  radiologically, endoscopically or surgically |  | x | x |
| Shi et al. | Anatomotic leakage | 30 days | Prediction | AL was defined as defect anastomosis; manifested radiological changes after surgery (with or without clinical intervention); color turbidity, faecal, or  other indicative changes observed from in the drain fluid; the peri-anastomotic abscess and angiogenic intra-abdominal infection was also considered as AL. |  |  | x |
| Van Kooten et al. | Anatomotic leakage | 30 days | Prediction | Anastomotic leakage was defined as any clinically or radiologically proven anastomotic leakage. |  | x | x |
| Cheng, Bai et al. 2023 | Postoperative central nervous system infection | 30 days | Prediction | Clinical Diagnostic Criteria:  Patients with fever, intracranial hypertension, turbid or purulent cerebrospinal fluid (CSF), leukocytosis, glucose < 2.2 mmol/L, and CSF glucose content/serum glucose content ≤ 0.4.  Etiological Diagnostic Criteria  Patients with positive microbiological cultures of specimen smears, drainage tube tips, implants, and CSF on the basis of clinical diagnosis, excluding those with bacterial contamination and colonization. | x | x | x |
| Martin et al. | All SSIs | 30 days | Prediction | SSI requiring IV antibiotics or hospital admission, seroma requiring drainage, incision dehiscence and explanation | x | x |  |

* The term physician for the purpose of application of the NHSN SSI criteria may be interpreted to mean a surgeon, infectious disease physician, emergency physician, other physician on the case, or physician’s designee (nurse practitioner or physician’s assistant).
** The following do not qualify as criteria for meeting the NHSN definition of superficial SSI: cellulitis, stitch abscess, localized stab wound or pin site infection.
*** Specific sites of an organ/space SSI: osteomyelitis, breast abscess or mastitis, myocarditis or pericarditis, disc space infection, ear/mastoid infection, endometritis, endocarditis, gastro-intestinal tract infection, intraabdominal infection, intracranial infection, joint or bursa infection, other infection of the lower respiratory tract, mediastinitis, meningitis or ventriculitis, oral cavity infection, deep pelvic tissue infection or other infection of the male or female reproductive tract, periprosthetic joint infection, spinal abscess, sinusitis, upper respiratory tract, pharyngitis, laryngitis, epiglottitis, urinary system infection, arterial or venous infection, vaginal cuff infection.
****Note: most surgical wounds that have broken down rapidly become colonized with bacteria. Bacterial growth from a wound is only significant when a sample to identify organisms by microbiological culture is collected aseptically under sterile conditions with symptoms of infection also present.
*****Medical records were reviewed to determine the indications of the interventions used in our composite endpoint. Infectious complications potentially related to perioperative interventions, but remote from the surgical site, including Clostridium difficile colitis, urinary tract infections, and pneumonia, were not included in the composite postoperative complication endpoint

**Table S4:** Infection definitions for pneumonia. Per definition and per criterion in the definition, it is indicated whether it is available from structured EHR data (tabular variables such as laboratory results, vital signs), available from free text (e.g., redness), or available from other sources (e.g., imaging).

| **Study or guideline or surveillance method** | **Timeframe** | **Definition used for (diagnosis, prediction, surveillance)** | **Definition in words** | **Criteria available from structured data** | **Clinical criterion (could be available from unstructured (text) data)** | **Criteria available from other sources (e.g., imaging)** |
| --- | --- | --- | --- | --- | --- | --- |
| ACS NSQIP/CDC | < 30 days | Diagnosis | Radiology*: One definitive chest radiological exam (x-ray or CT) with at least one of the following:  •New or progressive and persistent infiltrate  •Consolidation or opacity  •Cavitation  **AND**Signs/Symptoms/Laboratory: at least one of the following:  •Fever (>38 C or >100.4 F) with no other recognized cause  •Leukopenia (<4000 WBC/mm3) or leukocytosis(≥12,000 WBC/mm3)  •For adults ≥ 70 years old, altered mental status with no other recognized cause  **AND** At least one of the following:  •5% Bronchoalveolar lavage (BAL) -obtained cells contain intracellular bacteria on direct microscopic exam (e.g., Gram stain)  •Positive growth in blood culture not related to another source of infection  •Positive growth in culture of pleural fluid  •Positive quantitative culture from minimally contaminated lower respiratory tract (LRT) specimen (e.g. BAL or protected specimen brushing)  **OR** At least two of the following:  •New onset of purulent sputum, or change in character of sputum, or increased respiratory secretions, or increased suctioning requirements  •New onset or worsening cough, or dyspnea, or tachypnea  •Rales or rhonchi  •Worsening gas exchange (e.g. O2 desaturations (e.g., PaO2/FiO2 ≤ 240) increased oxygen requirements, or increased ventilator demand) | X  X  X   X   X  X            X | X     X   X    X     X    X  X  X | X |
| ECDC |  | Diagnosis | Two or more serial chest X-rays or CT-scans with a suggestive image of pneumonia for patients with underlying cardiac or pulmonary disease. In patients without underlying cardiac or pulmonary disease one definitive chest X-ray or CT-scan is sufficient   **AND** at least one of the following symptoms: • Fever > 38 °C with no other cause  • Leukopenia (< 4 000 WBC/mm3) or leucocytosis (≥ 12 000 WBC/mm3)   **AND** at least one of the following (or at least two if clinical pneumonia only = PN 4 and PN 5)  • New onset of purulent sputum, or change in character of sputum (colour, odour, quantity, consistency)  • Cough or dyspnea or tachypnea  • Suggestive auscultation (rales or bronchial breath sounds), ronchi, wheezing  • Worsening gas exchange (for example, O2 desaturation or increased oxygen requirements or increased ventilation demand)  **AND** according to the used diagnostic method  (a) Bacteriologic diagnostic performed by: Positive quantitative culture from minimally contaminated LRT (1) specimen (PN 1)  — Broncho-alveolar lavage (BAL) with a threshold of ≥ 104 CFU (2)/ml or ≥ 5 % of BAL obtained cells contains intracellular bacteria on direct microscopic exam (classified on the diagnostic category BAL)  — Protected brush (PB Wimberley) with a threshold of ≥ 103 CFU/ml  — Distal protected aspirate (DPA) with a threshold of ≥ 103 CFU/ml Positive quantitative culture from possibly contaminated LRT specimen (PN 2)  (b) Alternative microbiology methods (PN 3)  — Positive blood culture not related to another source of infection  — Positive growth in culture of pleural fluid  — Pleural or pulmonary abscess with positive needle aspiration  — Histologic pulmonary exam shows evidence of pneumonia  — Positive exams for pneumonia with virus or particular germs (for example, Legionella, Aspergillus, mycobacteria, mycoplasma, Pneumocystis jirovecii):  — Positive detection of viral antigen or antibody from respiratory secretions (for example, EIA, FAMA, shell vial assay, PCR)  — Positive direct exam or positive culture from bronchial secretions or tissue  — Seroconversion (for example, influenza viruses, Legionella, Chlamydia)  — Detection of antigens in urine (Legionella)  (c) Others  — Positive sputum culture or non-quantitative LRT specimen culture (PN 4)  — No positive microbiology (PN 5) | X  X  X | X   X  X | X |
| Kinlin et al. |  | Prediction | Case managers recorded the occurrence of complications after CABG, including the development of nosocomial pneumonia. This outcome was defined on the basis of   (1) new onset of pneumonia during current hospitalization, as documented by a treating physician;   (2) positive results of sputum, blood, pleural, empyema, transtracheal, or transthoracic fluid cultures, compatible with the diagnosis and clinical findings of pneumonia; or   (3) chest radiograph diagnostic of pulmonary infiltrates.   Aspiration pneumonia was coded separately from nosocomial pneumonia and was not included in our primary analyses, because it was considered to be a unique clinical entity, with distinct pathophysiological processes, risk factors, and medical management strategies. | X  X | X  X | X |

***** Note: In patients with underlying pulmonary or cardiac disease (e.g. respiratory distress syndrome, bronchopulmonary dysplasia, pulmonary oedema, or chronic obstructive pulmonary disease), two or more serial chest radiological exams (x-ray or CT) are required. (Serial radiological exams should be taken no less than 12 hours apart, but not more than 7 days apart. The occurrence should be assigned on the date the patient first met all of the criteria of the definition (i.e, if the patient meets all PNA criteria on the day of the first x-ray, assign this date to the occurrence. Do not assign the date of the occurrence to when the second serial x-ray was performed).

**Table S5:** Infection definitions for urinary tract infections (UTI). Per definition and per criterion in the definition, it is indicated whether it is available from structured EHR data (tabular variables such as laboratory results, vital signs), available from free text (e.g., redness), or available from other sources (e.g., imaging).

| **Study or guideline or surveillance method** | **Timeframe** | **Definition used for (diagnosis, prediction, surveillance)** | **Definition in words** | **Criteria available from structured data** | **Clinical criterion (could be available from unstructured (text) data)** | **Criteria available from other sources (e.g., imaging)** |
| --- | --- | --- | --- | --- | --- | --- |
| ACS NSQIP/ (E)CDC | 30 days | Diagnosis | An infection in the urinary tract (kidneys, ureters, bladder, and urethra). Must be noted within 30 days after the principal operative procedure **AND** patient must meet **ONE** of the following:  A: **ONE** of the following six criteria:  •fever (>38oC or 100.4o F)  •urgency, frequency, dysuria, suprabubic tenderness frequency or costovertebral angle pain or tenderness  **AND** A urine culture of > 100,000 colonies/ml urine with no more than two species of organisms. Signs and symptoms should be reported within 72 hours prior to a urine culture being sent or 24 hours after the culture was sent.  **OR**  B: **TWO** of the following six criteria:  •fever (>38o C or 100.4o F)  •urgency, frequency, dysuria, suprabubic tenderness frequency or costovertebral angle pain or tenderness  **AND** At least one of the following:  •Dipstick test positive for leukocyte esterase and/or nitrate  •Pyuria (>10 WBCs/mm3 or > 3 WBC/high power field of unspun urine)  •Organisms seen on Gram stain of unspun urine  •Two urine cultures with repeated isolation of the same uropathogen with >100,000 colonies/ml urine in non-voided specimen. Signs and symptoms should be reported within 72 hours prior to a urine culture being sent or 24 hours after the culture was sent. Urine culture with < 100,000 colonies/ml urine of single uropathogen in patient being treated with appropriate antimicrobial therapy. Signs and symptoms should be reported within 72 hours prior to a urine culture being sent or 24 hours after the culture was sent.  •Physician's diagnosis  •Physician institutes appropriate antimicrobial therapy | X  X  X  X  X  X  X  X  X | X  X  X  X  X  X  X |  |
| Cheng, Liu et al. 2022 | 30 day | Prediction | UTI was diagnosed when patients were found to have positive urine bacterial culture results with at least one of the following symptoms: urinary frequency, urgency, hematuria, urinary retention, dysuria, suprapubic pain, or febrile reactions. |  | **x** | **x** |

**Table S6:** Infection definitions for sepsis/septic shock. Per definition and per criterion in the definition, it is indicated whether it is available from structured EHR data (tabular variables such as laboratory results, vital signs), available from free text (e.g., redness), or available from other sources (e.g., imaging).

| **Study or guideline or surveillance method** | **Timeframe** | **Definition used for (diagnosis, prediction, surveillance)** | **Definition in words** | **Criteria available from structured data** | **Clinical criterion (could be available from unstructured (text) data)** | **Criteria available from other sources (e.g., imaging)** |
| --- | --- | --- | --- | --- | --- | --- |
| ACS NSQIP | N/A | Diagnosis | Sepsis is a vast clinical entity that takes a variety of forms. The spectrum of disorders spans from relatively mild physiologic abnormalities to septic shock. The most significant level is reported using the following criteria:  SIRS (Systemic Inflammatory Response Syndrome):  SIRS is a widespread inflammatory response to a variety of severe clinical insults. This syndrome is clinically recognized by the presence of **two or mor**e of the following within the same time frame:   - Temp >38 degrees C or <36 degrees C - HR >90 bpm - RR >20 breaths/min or PaCO2 <32 mmHg(<4.3 kPa) - WBC >12,000 cell/mm3, <4000 cells/mm3, or >10% immature (band) forms - Anion gap acidosis: this is defined by either:[Na + K] –[CL + HCO3 (or serum CO2]. If this number is greater than 16, then an anion gap acidosis is present. Na –[CL + HCO3 (or serum CO2]. If this number is greater than 12, then An anion gap acidosis is present. *If anion gap lab values are performed at your facilities lab, ascertain which formula is utilized and follow guideline criteria.   Definition revised or clarified from 2011 Sepsis: Sepsis is the systemic response to infection. Report this variable if the patient has clinical signs and symptoms of SIRS listed above **and** meets either A or B:  A. One of the following:  Positive blood culture.  Clinical documentation of purulence or positive culture from any site for which there is documentation noting the site as the acute case of sepsis.  B. Suspected pre-operative clinical condition of infection, or bowel infarction, which leads to the surgical procedure. The findings during the Principal Operative Procedure must confirm this suspected diagnosis with one or more of the following: Confirmed infarcted bowel requiring resection, purulence in the operative site, enteric contents in the operative site, or positive intra-operative cultures. | X X X  X  X  X | X  X |  |
| ACCP/SCCM (sepsis-3 criteria) |  | Diagnosis | qSOFA:  1) Respiratory rate  2) Mental status  3) Systolic blood pressure  SOFA:  1) PaO2/FiO2 ratio  2) Glasgow coma scale  3) Mean aterial pressure  4) Administration of vasopressors  5) Serum creatinine/urine output  6) Bilirubin  7) Platelet count  **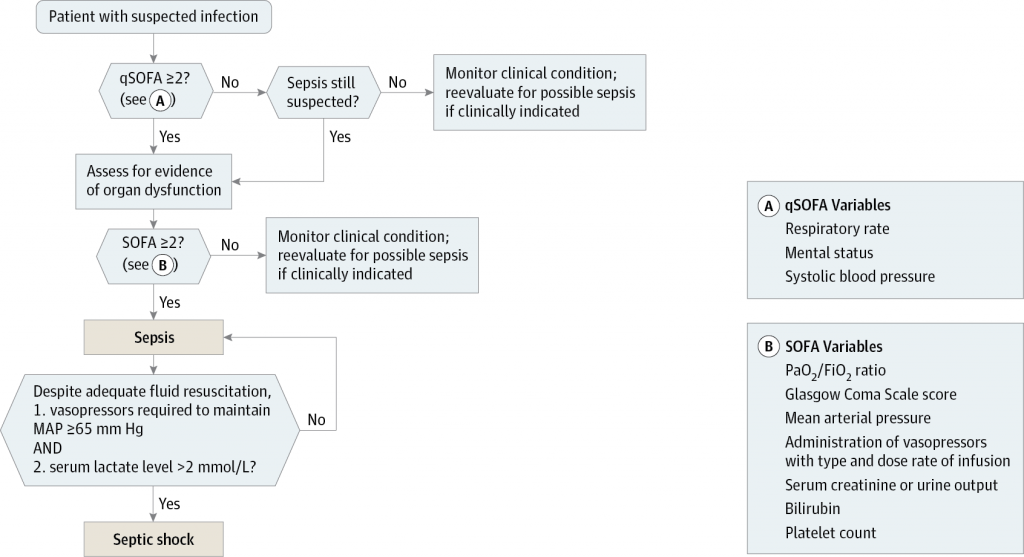** | X  X  X (if ventilated)  X (if recorded)  X  X  X  X  X | X |  |

**Table S7:** Infection definitions for nosocomial/healthcare associated/general infections. Per definition and per criterion in the definition, it is indicated whether it is available from structured EHR data (tabular variables such as laboratory results, vital signs), available from free text (e.g., redness), or available from other sources (e.g., imaging).

| **Study or guideline or surveillance method** | **Timeframe** | **Type of infections** | **Definition used for (diagnosis, prediction, surveillance)** | **Definition in words** | **Criteria available from structured data** | **Clinical criterion (could be available from unstructured (text) data)** | **Criteria available from other sources (e.g., imaging)** |
| --- | --- | --- | --- | --- | --- | --- | --- |
| WHO |  | **Nosocomial/Hospital acquired** | Surveillance | An infection acquired in hospital by a patient who was admitted for a reason other than that infection. An infection occurring in a patient in a hospital or other health care facility in whom the infection was not presented or incubating at the time of admission. This includes infections acquired in the hospital but appearing after discharge, and also occupational infections among staff of the facility. Simplified criteria for surveillance of nosocomial infections:  **SSI:**  Any purulent discharge, abscess or spreading cellulitis at the surgical site during the month after the operation.  **UTI:** Positive urine culture (1 or 2 species) with at least 10^5 bacteria/mL with or without clinical symptoms.  **Respiratory infection:** Respiratory symptoms with at least two of the following signs appearing during hospitalization:  -Cough  -Purulent sputum -New infiltrate on chest radiograph consistent with infection  **Vascular catheter infection:**  Inflammation, lymphangitis or purulent discharge at the insertion site of the catheter  **Septicaemia** Fever or rigours and at least one positive blood culture | X  X | X  X  X  X  X |  |
| (E)CDC |  | **Nosocomial/healthcare associated/general** |  | A nosocomial infection associated to the current hospital stay is defined as infection that matches one of the case definitions  **AND** — the onset of symptoms was on day 3 or later (day of admission = day 1) of the current hospital admission  **OR**  — the patient underwent surgery on day 1 or day 2 and develops symptoms of a Surgical Site Infection before day 3  **OR**  — an invasive device was placed on day 1 or day 2 resulting in an HAI before day 3  A nosocomial infection associated to a previous hospital stay is defined as an infection that matches one of the case definitions   **AND**  — the patient presents with an infection but has been readmitted less than 48 hours after a previous admission to an acute care hospital **OR**  — the patient has been admitted with an infection that meets the case definition of a Surgical Site Infection i.e. the SSI occurred within 30 days of the operation (or in the case of surgery involving an implant was a deep or organ/space SSI that developed within 90 days of the operation) and the patient either has symptoms that meet the case definition and/or is on antimicrobial treatment for that infection **OR**  — the patient has been admitted (or develops symptoms within 2 days) with Clostridium difficile infection less than 28 days from a previous discharge from an acute care hospital.   Note: For the purpose of point prevalence surveys, an active nosocomial infection present on the day of the survey is defined as an infection for which signs and symptoms of the infection are present on the survey date or signs and symptoms were present in the past and the patient is (still) receiving treatment for that infection on the survey date. The presence of symptoms and signs should be verified until the start of the treatment in order to determine whether the treated infection matches one of the case definitions of nosocomial infection |  |  |  |

#

#

# Appendix D: Data extraction tables automated surveillance studies

**Table S8:** Data extraction table for automated surveillance study

| Study ID | Title | Study design | Years of data collection | Patient population | Further specification | Sample size | Type of infections researched | Type of surveillance algorithms | Type of criteria included in the definition | Type of data used for algorithm | Type of clinical information used for algorithm | Definition(s) of infection used by automated surveillance algorithm |
| --- | --- | --- | --- | --- | --- | --- | --- | --- | --- | --- | --- | --- |
| Blacky 2011 [117] | Fully Automated Surveillance of Healthcare-Associated Infections with MONI-ICU: A Breakthrough in Clinical Infection Surveillance | Cohort study - Diagnostic test accuracy study | 2006-2007 | ICU | Adult patients with admissions > 48 hours | 99 (1007 patient days) | Bloodstream infections; Pneumonia; UTI; Other: Central venous catheter-related infection | Set of rules/criteria; Other classification algorithm | Structured, tabular, EHR data | A, C | N/A | MONI-ICU algorithm that uses a fuzzy set and fuzzy logic. Not further specified. |
| Bouam 2003 [118] | An intranet-based automated system for the surveillance of nosocomial infections: prospective validation compared with physicians' self-reports | Cohort study - Diagnostic test accuracy study | 2000 | Surgical ; ICU | Admitted for more than 24 hours | 548 | Bloodstream infections; UTI; Other: Catheter-related infections | Set of rules/criteria | Structured, tabular, EHR data; Text data (using NLP) | C | 3 | Automated surveillance based on culture data (not further specified) |
| Bouzbid 2011 [119] | Automated detection of nosocomial infections: evaluation of different strategies in an intensive care unit 2000-2006 | Cohort study - Diagnostic test accuracy study | 2000-2006 | ICU | Adult patients admitted for medical or surgical problems in an ICU of the University Hospital of Lyon | 1499 | Bloodstream infections; Pneumonia; UTI; Other: Central venous catheter-associated infections (CVC-AIs) | Set of rules/criteria | Structured, tabular, EHR data; Text data (using NLP) | A, C | 3, 5, 6a | Best strategy: ATB (Anatomical Therapeutic Classification) or MAC (Microbiological algorithm).  ATB: (Pneumonia, CVC-AI, UTI, BSI)  -antibiotic prescription (ATC: J01) and  -antibiotic prescription >48 h after ICU admission or time antibiotic prescription changed>48 h after ICU admission  MAC: (CVC-AI, UTI)  -Sampling collected from the anatomical site >48 h after ICU admission and  -Pathogen isolated  MAC: (BSI)  -Blood culture collected >= 2 days from ICU admission and  -Pathogen other than common skin contaminants (CSCs) corresponding to coagulase-negative staphylococci, micrococci, Bacillusspp., Corynebacteriumspp., Propionibacteriumspp., cultured from blood, or >=2 CSC isolates recovered from blood within 48 h of initial positive blood culture |
| Branch-Elliman 2015 [120] | Natural Language Processing for Real-Time Catheter-Associated Urinary Tract Infection Surveillance: Results of a Pilot Implementation Trial | Other: Observational study | 2013 | ICU; Other: Acute care units (ACU) |  | 43609 patient days | Other: Catheter-associated urinary tract infections (CAUTIs) | NLP algorithm | Structured, tabular, EHR data; Text data (using NLP) | A, B | 2, 3, 4 | NLP-augmented algorithm, combined with structured data (vital signs, microbiology, urinalysis):  CAUTI:  - Progress notes (NLP): Foley catheter day 3 or greater, AND  - "fever" detected by NLP algorithm AND/OR recorded temperature >= 100.4 F AND  - any positive urine culture |
| Campillo-Gimenez 2013 [121] | Full-text automated detection of surgical site infections secondary to neurosurgery in Rennes, France | Cohort study - Diagnostic test accuracy study | 2008-2010 | Surgical | Neurosurgery | 5010 | All SSI | NLP algorithm | Structured, tabular, EHR data; Text data (using NLP) | A, B | 1, 2 | 1. ICD-10 codes (DRG database)  2. NLP of full-text medical reports |
| Cato 2017 [122] | Electronic Surveillance of Surgical Site Infections | Cohort study | 2009-2012 | All |  | 443284 | Bloodstream infections; Pneumonia; All SSI; UTI; Other: Intermediate infection for all the above | Set of rules/criteria | Structured, tabular, EHR data; Other: Microbiology data | A, C | 1, 3 | 1. Bloodstream  infection  (+) blood culture AND  no other (+) culture with same organism at another body site in previous 14d  2. Pneumonia  ICD-9-CM code for pneumonia AND  (+) respiratory culture  3. SSI  ICD-9-CM code for NHSN procedure AND  (+) wound culture within 30 d  4. UTI  [(+) urine culture of >= 105 CFU/mL with <2 otherspecies] OR  [(+) urine culture of 103–105 CFU/mL with <2 other species AND  pyuria (>=WBC per high power field in urine microscopy) within +- 48h of culture]  See for intermediate definitions Table 1. |
| Choudhuri 2011 [123] | An electronic catheter-associated urinary tract infection surveillance tool | Other: Retrospective validation study | 2010 | Other: Hospitalized atients with indwelling urinary catheters |  | 136 | Other: CAUTI | Set of rules/criteria | Structured, tabular, EHR data; Other: Microbiology data | A, C | 3, 4, 6c | Patient with urinary catheter  AND  No UTI in previous 14 days  AND  >= 10^3 CFU/ml  AND  T > 38C within 24h before or after culture taken  AND  ((Positive LE (leukocyte esterase)/Nitrite OR > 5 WBC/HPF)  OR  (>= 10^5 CFU/ml AND no UTI present on admission/catheter insertion)) |
| Dubberke 2012 [124] | Implementing automated surveillance for tracking Clostridium difficile infection at multiple healthcare facilities | Other: Retrospective validation study | 2005-2006 | All | That tested positive for Clostridium Difficile toxins | 1767 | Other: Clostridium difficile (c. difficile) | Set of rules/criteria | Structured, tabular, EHR data; Other: Microbiology data | C | 3, 5 | Positive C. diff stool toxin assay  AND  previous admission data (see Figure 1 for further information) to investigate whether it was hospital-acquired or not |
| Ehrentraut 2018 [125] | Detecting hospital-acquired infections: A document classification approach using support vector machines and gradient tree boosting | Other: Retrospective validation study | 2012 |  | Not specified | 120 patients, 213 admissions | HAI | NLP algorithm | Structured, tabular, EHR data; Text data (using NLP) | B | 2 | NLP algorithm: support vector machines and GTB |
| FitzHenry 2013 [126] | Exploring the Frontier of Electronic Health Record Surveillance The Case of Postoperative Complications | Cohort study | 1999-2006 | Surgical | At six veterans affairs centers | 8186 | Sepsis; Pneumonia; All SSI; UTI | Set of rules/criteria | Text data (using NLP) | B | 2 | Based on VASQIP definitions, the complications were identified. The narrative clinical notes were parsed electronically for medical terminology and mapped to SNOMED CT concepts using the Multithreaded Clinical Vocabulary Server Natural Language Processing program. |
| Fu 2021 [127] | Automated Detection of Periprosthetic Joint Infections and Data Elements Using Natural Language Processing | Other: Retrospective case-control study | 2000-2017 | Surgical | Patients who underwent primary total hip and knee arthroplasty procedures | 48962 | Other: Periprosthetic joint infection (PJI) | NLP algorithm | Text data (using NLP) | B | 2 | NLP algorithm on surgical report, infectious disease consultation notes, pathology report, and microbiology report |
| Leal 2010 [128] | Development of a novel electronic surveillance system for monitoring of bloodstream infections | Cohort study - Diagnostic test accuracy study | 2005 | Other: N/A |  | 2281, 306 included for validation | Bloodstream infections | Set of rules/criteria | Other: Culture data | C | 3 | Pathogen found in >1 set of blood cultures.  OR  Organisms linked to contamination found in >2 sets of blood cultures within 5 days (Diptheroids, Bacillus species, Propionibacterium species, coagulase-negative staphylococci, and/or micrococci). |
| Leal 2016 [129] | The Validation of a Novel Surveillance System for Monitoring Bloodstream Infections in the Calgary Zone | Cohort study - Diagnostic test accuracy study | 2007 | Other: All patients with positive cultures |  | 308 | Bloodstream infections | Set of rules/criteria | Other: Microbiology data | C | 3 | Pathogen found in >1 set of blood cultures.  OR  Organisms linked to contamination found in >2 sets of blood cultures within 5 days (Diptheroids, Bacillus species, Propionibacterium species, coagulase-negative staphylococci, and/or micrococci). |
| Leclère 2014 [130] | Matching bacteriological and medico-administrative databases is efficient for a computer-enhanced surveillance of surgical site infections: retrospective analysis of 4,400 surgical procedures in a French university hospital | Cohort study | 2014 | Surgical | Nine surgical procedures:  Coronary artery bypass grafting (CABG)  Valve replacement  Elective (nonurgent) colectomy with immediate restoration of intestinal continuity  Primary total hip arthroplasty (PTHA)  Primary total knee arthroplasty (PTKA)  Kidney transplantation  Urinary sphincter implantation  Neurostimulator implantation  Cesarean section (C-section) | 4400 procedures | All SSI | Set of rules/criteria | Structured, tabular, EHR data; Other: Microbiology data | A, C | 1, 3 | 1. ICD-10 codes (T81.4, O86.0, T85.7, T83.5, T82.7, T82.6, K65.0, K65.8, K65.9, T81.38, T81.5)  2. Microbiology results: Positive cultures, depending on the sample type (blood, stool, urine, biopsy, deep drainage, etc), samples were classified as "deep" or "superficial". Samples had to be taken < 40 days of procedure, or < 400 day for prosthetic surgery.  3. Combination of 1. and 2. |
| Leth 2010 [131] | Surveillance of selected post-caesarean infections based on electronic registries: validation study including post-discharge infections | Cohort study | 2007-2008 | Surgical | Caesarean sections | 1513 | All SSI; UTI | Set of rules/criteria | Structured, tabular, EHR data; Other: Microbiology data | A, C | 1, 3, 6a, 6b | 1. UTI  Culture of urine of >= 105cfu/mL of a dominating pathogen or alternatively < 105cfu/mL of a single culture of pathogen AND/OR  UTI-specific antibiotic treatment (ATC J01EB02, J01EA01, J01CA08, J01XE01)  2. Postoperative wound infection  Culture-positive swab from wound/drainage  AND/OR  Discharge code for postoperative infection (ICD-10: T81.4 or O86.0)  AND/OR  Code for reoperation due to wound infection (WB00, KMWB00, KLWC00, KLWC01, KMWC00, KMWC01,according to the Nordic Classification of Surgical Procedures, Uppsala, Sweden, 2nd revision.)  AND/OR  relevant antibiotic treatment (ATC J01CF01) |
| Lin 2014 [132] | Multicenter evaluation of computer automated versus traditional surveillance of hospital-acquired bloodstream infections | Cohort study | 2004-2007 | ICU | 4 academic medical centers | 1251 blood culture episodes, 664 randomnly selected for analyses | Bloodstream infections | Set of rules/criteria | Other: Microbiology data | C | 3, 5 | If common skin commensal (CSC): infection if >= 2 CSCs (same species)  If non-CSC: All are considered infections.  Hospital acquired: only infections occurring >3rd hospital day |
| Peterson 2012 [133] | Electronic surveillance for infectious disease trend analysis following a quality improvement intervention | Cohort study | 2003-2010 | All | 3 hospitals | 342492 admissions | Other: MRSA | Other classification algorithm; Other: Nosocomial Infection Marker (NIM) from CareFusion's MedMined system, laboratory information system (LIS) method, and LIS + admission-discharge-transfer (ADT) data method | Structured, tabular, EHR data; Text data (using NLP); Other: Microbiology data | C | 3 | NIM uses a proprietary algorithm and microbiology and ADT data to establish putative healthcare-associated infections. LIS method identifies positive cultures and excludes certain specimen sources. LIS + ADT method applies several rules to identify putative MRSA healthcare-associated infections. |
| Redder 2015 [134] | Incidence rates of hospital-acquired urinary tract and bloodstream infections generated by automated compilation of electronically available healthcare data | Cohort study | 2010-2014 | Other: Internal medicine department |  | 285215 | Bloodstream infections; UTI | Set of rules/criteria | Structured, tabular, EHR data; Other: Microbiology data | A, C | 3, 6a | 1. Hospital-acquired UTI:  (i) positive urine culture containing ≥105 cfu/mL of a pathogen and UTI-relevant antibiotic treatment;  (ii) positive urine culture containing ≥104 cfu/mL of pure culture of a recognised uropathogen, e.g. Escherichia coli, and UTI-relevant antibiotic treatment;  (iii) UTI-specific antibiotic treatment (mecillinam/pivmecillinam, nitrofurantoin, sulphamethizole or trimethoprim) prescribed without the presence of a positive urine culture  2. Hospital-acquired BSI:  A positive blood culture with a significant pathogen AND  concomitant antibiotic treatment of the patient |
| Sakji 2010 [135] | Evaluation of a French medical multi-terminology indexer for the manual annotation of natural language medical reports of healthcare-associated infections | Cohort study | 2009-2011 | Surgical ; ICU | Patients with hospital-acquired infections managed in intensive care units, orthopedic surgery, and digestive surgery departments of the Lyon University Hospital | 2000 overall, 400 with HAI reports and 400 without. | HAI | NLP algorithm | Text data (using NLP) | B | 2 | NLP model on symptoms, diagnosis, medical interventions, medication, microorganisms, medical imaging etc. |
| Suzuki 2021 [136] | Development of a fully automated surgical site infection detection algorithm for use in cardiac and orthopedic surgery research | Cohort study | 2007-2018 | Surgical | Patients who underwent coronary artery bypass grafting, valve replacement, or total joint arthroplasty (TJA) at 11 VA (veterans' affairs) hospitals. | 9,557 cardiac surgeries and 12,992 TJAs evaluated by VASQIP | Deep SSI; Organ space SSI | Set of rules/criteria; Other: Logistic regression to select model variables | Structured, tabular, EHR data; Text data (using NLP) | A, B, C | 1, 2, 3, 5, 6a, 6b | Cardiac surgery:  - ICD codes for SSI = 2 points  - ICD code for mediastinitis = 1 point  - Positive culture for S. Aureus = 1 point  - Sternal debridement = 2 points  TJA:  - ICD codes for definite SSIs = 2 points  - ICD codes for other diagnosis for possible SSIs = 2 points  - any consultation note mentioning hip or knee surgical infection = 1 point  - reoperation = 1 point  - readmission=1 point  - positive culture for Staphylococcus aureus = 1 point  - Antibiotics: use of vancomycin or piperacillin/tazobactam ≥ 6 days = 1 point  - Antibiotics: use of rifampin = 1 point. |
| Thirukumaran [137] 2019 | Natural Language Processing for the Identification of Surgical Site Infections in Orthopaedics | Cohort study | 2011-2017 | Surgical | Orthopedic patients | 1579 | Superficial SSI; Deep SSI; Other: Within 90 days of the primary surgical procedure | NLP algorithm | Structured, tabular, EHR data; Text data (using NLP) | B | 2 | Free-text notes such as progress notes, discharge summaries, history and physical examination notes, and telephone encounter notes.  1. Variables from administrative data only  2. NLP keywords only  3. Stemmed and lemmatized versions of NLP keywords only  4. 5-grams only  5. Model 1 & model 2 combined  6. Model 1 & model 3 combiend  7. Model 1 & model 4 combined |
| Tvardik 2018 [138] | Accuracy of using natural language processing methods for identifying healthcare-associated infections | Cohort study | 2009-2010 | Surgical ; ICU | Digestive surgery, neurosurgery, orthopedic surgery, adult ICU | 1531 medical records, 120 patients selected for the evaluation | HAI; Bloodstream infections; Pneumonia; All SSI; UTI; Other: Infections of the central venous catheter | NLP algorithm | Text data (using NLP) | B | 2 | NLP algorithm |
| Valik 2020 [139] | Validation of automated sepsis surveillance based on the Sepsis-3 clinical criteria against physician record review in a general hospital population: observational study using electronic health records data | Cohort study | 2012-2013 | Other: All adult patients admitted, ICU censored |  | 82653 admissions of 54884 patients | Other: Sepsis | Set of rules/criteria | Structured, tabular, EHR data; Other: Microbiology data | A, C | 3, 4, 6a | Suspected infection: having any culture taken AND at least two doses of antimicrobials administered, started < 72h after culture.  AND  SOFA score >= 2 points |
| vanderWerff 2021 [140] | The accuracy of fully automated algorithms for surveillance of healthcare-associated urinary tract infections in hospitalized patients | Cohort study | 2010-2013 | All |  | 2979 admissions | UTI | Set of rules/criteria; NLP algorithm | Structured, tabular, EHR data; Text data (using NLP); Other: Microbiology results | A, B, C | 1, 2, 3, 4, 6a, 7 | 1. Positive urine culture  2. Positive urine culture  AND  UTI related ICD-10 codes during admission  3. Positive urine culture combined  AND  UTI-specific antibiotics (appendix)  4. Positive urine culture with fever  AND/OR  UTI symptoms in accordance with ECDC/CDC definitions  5. Algorithm 4. with negating rule for cases with fever AND without UTI symptoms by non-correpondent positive blood cultures OR relevant ICD-10 codes  Positive urine culture was defined as urine culture with no more than two pathogens (any bacteria or fungi except mixed flora) and with at least one pathogen having> 105 colony-forming units (cfu) per millilitre of urine. |
| Venable 2013 [141] | Is automated electronic surveillance for healthcare-associated infections accurate in the burn unit? | Cohort study | 2011 | Other: Burn unit |  | 500 | Bloodstream infections; UTI | Set of rules/criteria | Structured, tabular, EHR data; Other: Microbiology data | A, B | N/A | Proprietary electronic surveillance system (Medmined® by CareFusion) |
| Wald 2014 [142] | Accuracy of electronic surveillance of catheter-associated urinary tract infection at an academic medical center | Cohort study - Diagnostic test accuracy study | 2009-2010 | Other: Inpatients with high clinical suspicion of CAUTI |  | 1695 unique inpatient encounters | Other: CAUTI | Set of rules/criteria | Structured, tabular, EHR data; Other: Microbiology data | A, C | 3, 4, 5 | Based on CDC/NHSN criteria.  (1) a UTI (a positive urine culture from a patient with either fever or symptoms referable to the urinary tract or from a patient with a positive blood culture matching the urine culture),  (2) diagnosed from culture sent more than 48 hours after admission to the hospital, and  (3) diagnosed in a patient who has had an IUC in place or removed within 48 hours before the culture  The algorithm differed from the CDC’s definition in the following ways:  (1) subjective symptoms referable to the urinary tract were not incorporated and  (2) 2 calendar days was used for attribution of a positive culture to a catheter and hospital in lieu of 48 hours. |
| Woeltje 2011 [143] | Electronic surveillance for healthcare-associated central line-associated bloodstream infections outside the intensive care unit | Cohort study | 2005-2006 | Other: Patients with positive blood culture | Non-ICU wards | 391 positive cultures from 331 patients | Other: Central line-associated bloodstream infections (CLABSIs) | Set of rules/criteria | Structured, tabular, EHR data; Other: Microbiology data | A, C | 3, 4, 6c | The best-fit model included:  - the presence of a catheter  AND  - blood culture positive for known pathogen  OR  - blood culture with a common skin contaminant confirmed by a second positive culture AND the presence of fever, AND no positive cultures with the same organism from another sterile site |
| vanMourik 2011 [144] | Automated detection of external ventricular and lumbar drain-related meningitis using laboratory and microbiology results and medication data | Cohort study | 2004-2009 | Other: All patients receiving a external ventricular drain (EVD) or lumbar drain (ELD) |  | 742 | Other: External ventricular and lumbar drain-related meningitis | Other classification algorithm | Structured, tabular, EHR data; Other: Microbiology data | A, C | 6c, 8 | Logistic regression model, including:  - number of drains placed  - drain type  - blood leukocyte count  - CRP  - cerebrospinal fluid leucoyte count  - culture result |
| Colborn 2023 [145] | Development and validation of models for detection of postoperative infections using structured electronic health records data and machine learning | Cohort study | 2013-2019 | Surgical | Linked to ACS NSQIP | 30,639 procedures | SSI, UTI, sepsis, pneumonia | Other classification algorithm | Structured | A | 1, 6a, 7 | Logistic regression models using ICD and other complication registration codes, antibiotics prescription, taking cultures. See article for regression coëfficients |
| Stern 2023 [146] | Electronic surveillance criteria for non-ventilator-associated hospital-acquired pneumonia: Assessment of reliability and validity | Cohort study | 2015-2020 | All | Admited to veterans’ affairs hospitals | 3,1 million | Non-ventilator associated pneumonia | Set of rules/criteria | Structured, tabular, EHR data, | A, D | 4, 6a, 8 | Worsening oxygenation for >= 2 calendar days, fever or abnormal WBC, performance of chest imaging, initiation of new antibiotics. |
| Van der Werff 2022 [147] | The accuracy of fully automated algorithms for surveillance of healthcare-onset Clostridioides difficile infections in hospitalized patients | Cohort study | 2011-2013 | All |  | 179,131 | C.Difficile | Set of rules/criteria | Structured, tabular EHR data, Microbiology results | C | 3 | Positive stool sample with C. difficile toxin or toxin-producing C. difficile. |

#

# Appendix E: Definitions with further specified criteria

**Table S9.** Definitions to identify patients with bacterial hospital acquired infections (HAI, subtype not further specified). Type A = structured EHR data, including tabular information stored such as complication registries, medication information, and vital signs, Type B = Free-text clinical notes, including all clinical information stored in free-text such as discharge letters and daily reports, Type C = Microbiology results, Type D = Imaging results.

|  |  |  | Information used to identify patients with infections based on EHR data | | | | | | | | | |  |
| --- | --- | --- | --- | --- | --- | --- | --- | --- | --- | --- | --- | --- | --- |
| Origin of definition | Reference | Data type category | ICD-  codes | Free-text | Cultures | Vitals | Admission data | ABx | Surg. intervention | Lines or  catheter | Lab results | Imaging | Clavien- Dindo (minimum) |
| Diagnostic guidelines | WHO | A,B,C,D |  | x | x | x |  |  |  |  |  | x | 1 |
| Diagnostic guidelines | ECDC | A, B |  | x |  |  | x |  |  |  |  |  | 1 |
| Automated surveillance | Ehrentraut | B |  | x |  |  |  |  |  |  |  |  | 1 |
| Automated surveillance | Sakji | B |  | x |  |  |  |  |  |  |  |  | 1 |
| Automated surveillance | Tvardik | B |  | x |  |  |  |  |  |  |  |  | 1 |

**Table S10:** Definitions to identify patients with pneumoniae. Type A = structured EHR data, including tabular information stored such as complication registries, medication information, and vital signs, Type B = Free-text clinical notes, including all clinical information stored in free-text such as discharge letters and daily reports, Type C = Microbiology results, Type D = Imaging results.

|  |  |  | Information used to identify patients based on EHR data | | | | | | | | | |  |
| --- | --- | --- | --- | --- | --- | --- | --- | --- | --- | --- | --- | --- | --- |
| Origin of definition | Reference | Data type category | ICD-  codes | Free-text | Cultures | Vitals | Admission data | ABx | Surg. intervention | Lines or  catheter | Lab results | Imaging | Clavien Dindo (minimum) |
| Diagnostic guidelines | (E)CDC/ASC NSQIP | A, B, C, D |  | x | x | x |  |  |  |  | x | x | 1 |
| Prediction modelling | Kinlin et al. | A, B, C, D | x | x | x |  |  |  |  |  |  | x | 1 |
| Automated surveillance | Blacky | A, C |  |  |  |  |  |  |  |  |  |  |  |
| Automated surveillance | Bouzbid | A, C |  |  | x |  | x | x |  |  |  |  | 2 |
| Automated surveillance | Cato | A, C | x |  | x |  |  |  |  |  |  |  | 1 |
| Automated surveillance | FitzHenry | B |  | x |  |  |  |  |  |  |  |  | 1 |
| Automated surveillance | Tvardik | B |  | x |  |  |  |  |  |  |  |  | 1 |
| Automated surveillance | Colborn | A | x |  |  |  |  | x |  |  | x |  | 1 |
| Automated surveillance | Stern | A, D |  |  |  | x |  | x |  |  |  | x | 1 |

###

**Table S11:** Definitions to identify patients with SSIs. Type A = structured EHR data, including tabular information stored such as complication registries, medication information, and vital signs, Type B = Free-text clinical notes, including all clinical information stored in free-text such as discharge letters and daily report, Type C = Microbiology results, Type D = Imaging results.

|  |  |  | Information used to identify patients based on EHR data | | | | | | | | | |  |
| --- | --- | --- | --- | --- | --- | --- | --- | --- | --- | --- | --- | --- | --- |
| Origin of definition | Reference | Data type category | ICD-  Codescodes | Free-text | Cultures | Vitals | Admission data | ABx | Surg. intervention | Lines or  catheter | Lab results | Imaging | Clavien Dindo (Minimum) |
| Diagnostic guidelines | CDC/ASC NSQIP | A, B, C, D | x | x | x | x |  |  | x |  |  | x | 1-3a |
| Diagnostic guidelines | WHO | A, B, C, D |  | x | x | x |  |  | x |  |  | x | 1-3a |
| Prediction modelling | Daneman et al. | A | x |  |  |  |  |  |  |  |  |  | 1 |
| Prediction modelling | Weller et al. | A |  |  |  |  |  | x | x |  |  |  | 2 |
| Prediction modelling | Crispin et al. | A |  |  |  |  |  |  | x |  |  |  | 3a |
| Prediction modelling | Martin et al. | A,B |  | x |  |  | x | x | x |  |  |  | 2 |
| Automated surveillance | Campillo-Gimenez | A, B | x | x |  |  |  |  |  |  |  |  | 1 |
| Automated surveillance | Cato | A, C | x |  | x |  |  |  |  |  |  |  | 1 |
| Automated surveillance | FitzHenry | B |  | x |  |  |  |  |  |  |  |  | 1 |
| Automated surveillance | Leclere | A, C | x |  | x |  |  |  |  |  |  |  | 1 |
| Automated surveillance | Leth | A, C |  |  | x |  |  | x |  |  |  |  | 2 |
| Automated surveillance | Suzuki | A, B, C | x | x | x |  | x | x | x |  |  |  | 2 |
| Automated surveillance | Tvardik | B |  | x |  |  |  |  |  |  |  |  | 1 |
| Automated surveillance | Thirukumaran | B |  | x |  |  |  |  |  |  |  |  | 1 |
| Automated surveillance | Colborn | A | x |  |  |  |  | x |  |  | x |  | 1 |

**Table S12:** Definitions to identify patients with anastomotic leakage and abdominal infections. Type A = structured EHR data, including tabular information stored such as complication registries, medication information, and vital signs, Type B = Free-text clinical notes, including all clinical information stored in free-text such as discharge letters and daily report, Type C = Microbiology results, Type D = Imaging results.

|  |  |  | Information used to identify patients based on EHR data | | | | | | | | | |  |
| --- | --- | --- | --- | --- | --- | --- | --- | --- | --- | --- | --- | --- | --- |
| Origin of definition | Reference | Data type category | ICD-  codes | Free-text | Cultures | Vitals | Admission data | ABx | Surg. intervention | Lines or  catheter | Lab results | Imaging | Clavien Dindo (Minimum) |
| Diagnostic guidelines | Study Group of Rectal Cancer AL definition | A, B, D |  | x |  |  |  |  |  |  | x | x | 1 |
| Prediction modelling | Stidham et al. | A |  |  |  |  |  | x | x | x |  |  | 3a |
| Prediction modelling | Miyakita et al. | A |  |  |  |  |  |  | x |  |  |  | 3b |
| Prediction modelling | Mckenna et al. | A |  |  |  |  |  | x | x |  |  |  | 2 |
| Prediction modelling | Nudel et al. | A, B |  | x |  |  | x |  | x | x |  |  | 3a |
| Prediction modelling | Kawai et al. | B, D |  | x |  |  |  |  |  |  |  | x | 2 |
| Prediction modelling | Lin 2022 et al. | B, D |  | x |  |  |  |  | x |  |  | x | 2 |
| Prediction modelling | Shi et al. | D |  |  |  |  |  |  |  |  |  | x | 1 |
| Prediction modelling | vanKooten et al. | B, D |  | x |  |  |  |  |  |  |  | x | 1 |

###

**Table S13:** Definitions to identify patients with urinary tract infections (UTIs). Type A = structured EHR data, including tabular information stored such as complication registries, medication information, and vital signs, Type B = Free-text clinical notes, including all clinical information stored in free-text such as discharge letters and daily report, Type C = Microbiology results, Type D = Imaging results.

|  |  |  | Information used to identify patients based on EHR data | | | | | | | | | |  |
| --- | --- | --- | --- | --- | --- | --- | --- | --- | --- | --- | --- | --- | --- |
| Origin of definition | Reference | Data type category | ICD-  Codes | Free-text | Cultures | Vitals | Admission data | ABx | Surg. intervention | Lines or  catheter | Lab results | Imaging | Clavien Dindo (Minimum) |
| Diagnostic guidelines | (E)CDC/ASC NSQIP | A, B, C | x | x | x | x |  | x |  |  | x |  | 2 |
| Prediction modelling | Cheng, Liu 2022. | B, C |  | x | x |  |  |  |  |  |  |  | 1 |
| Automated surveillance | Bouam 2003 | C |  |  | x |  |  |  |  |  |  |  | 1 |
| Automated surveillance | Bouzbid 2011 | A, C |  |  | x |  | x | x |  |  |  |  | 2 |
| Automated surveillance | Branch-Elliman | A, B |  | x | x | x |  |  |  |  |  |  | 1 |
| Automated surveillance | Cato 2017 | A, C | x |  | x |  |  |  |  |  |  |  | 1 |
| Automated surveillance | Choudhuri 2011 | A, C |  |  | x | x |  |  |  | x |  |  | 1 |
| Automated surveillance | FitzHenry 2013 | B |  | x |  |  |  |  |  |  |  |  | 1 |
| Automated surveillance | Leth 2010 | A, C | x |  | x |  |  | x | x |  |  |  | 2 |
| Automated surveillance | Redder 2015 | A, C |  |  | x |  |  | x |  |  |  |  | 2 |
| Automated surveillance | Tvardik 2018 | B |  | x |  |  |  |  |  |  |  |  | 1 |
| Automated surveillance | vanderWerff | A, B, C | x | x | x | x |  | x |  |  |  |  | 2 |
| Automated surveillance | Venable 2013 | A, B |  |  |  |  |  |  |  |  |  |  |  |
| Automated surveillance | Wald 2014 | A, C |  |  | x | x | x |  |  |  |  |  | 1 |
| Automated surveillance | Colborn | A | x |  |  |  |  | x |  |  | x |  | 1 |

###

**Table S14:** Definitions to identify patients with bloodstream infections. Type A = structured EHR data, including tabular information stored such as complication registries, medication information, and vital signs, Type B = Free-text clinical notes, including all clinical information stored in free-text such as discharge letters and daily report, Type C = Microbiology results, Type D = Imaging results.

|  |  |  | Information used to identify patients based on EHR data | | | | | | | | | |  |
| --- | --- | --- | --- | --- | --- | --- | --- | --- | --- | --- | --- | --- | --- |
| Origin of definition | Reference | Data type category | ICD-  Codes | Free-text | Cultures | Vitals | Admission data | ABx | Surg. intervention | Lines or  catheter | Lab results | Imaging | Clavien Dindo (Minimum) |
| Diagnostic guidelines | ACS NSQIP | A, C |  |  | x | x |  |  |  |  | x |  | 1 |
| Diagnostic guidelines | ACCP/SCCM (sepsis-3 criteria) | A |  |  |  | x |  | x |  |  | x |  | 2 |
| Automated surveillance | Blacky 2011 | A, C |  |  |  |  |  |  |  |  |  |  |  |
| Automated surveillance | Bouam 2003 | C |  |  | x |  |  |  |  |  |  |  | 1 |
| Automated surveillance | Bouzbid 2011 | A, C |  |  | x |  | x | x |  |  |  |  | 2 |
| Automated surveillance | Cato 2017 | A, C | x |  | x |  |  |  |  |  |  |  | 1 |
| Automated surveillance | FitzHenry 2013 | B |  | x |  |  |  |  |  |  |  |  | 1 |
| Automated surveillance | Leal 2010 | C |  |  | x |  |  |  |  |  |  |  | 1 |
| Automated surveillance | Leal 2016 | C |  |  | x |  |  |  |  |  |  |  | 1 |
| Automated surveillance | Lin 2014 | C |  |  | x |  | x |  |  |  |  |  | 1 |
| Automated surveillance | Redder 2015 | A, C |  |  | x |  |  | x |  |  |  |  | 2 |
| Automated surveillance | Tvardik 2018 | B |  | x |  |  |  |  |  |  |  |  | 1 |
| Automated surveillance | Valik 2020 | A, C |  |  | x | x |  | x |  |  |  |  | 2 |
| Automated surveillance | Venable 2013 | A, B |  |  |  |  |  |  |  |  |  |  |  |
| Automated surveillance | Woeltje 2011 | A, C |  |  | x | x |  |  |  | x |  |  | 1 |
| Automated surveillance | Colborn | A | x |  |  |  |  | x |  |  | x |  | 1 |

**Table S15:** Definitions to identify patients with other types of infection. Type A = structured EHR data, including tabular information stored such as complication registries, medication information, and vital signs, Type B = Free-text clinical notes, including all clinical information stored in free-text such as discharge letters and daily report, Type C = Microbiology results, Type D = Imaging results.

|  |  |  |  | Information used to identify patients based on EHR data | | | | | | | | | |  |
| --- | --- | --- | --- | --- | --- | --- | --- | --- | --- | --- | --- | --- | --- | --- |
| Type of infection | Origin of definition | Reference | Data type category | ICD-  codes | Free-text | Cultures | Vitals | Admission data | ABx | Surg. intervention | Lines or  catheter | Lab results | Imaging | Clavien Dindo (Minimum) |
| C. Difficile | Automated surveillance | Dubberke 2012 | C |  |  | x |  | x |  |  |  |  |  | 1 |
| C. Difficile | Automated surveillance | vanderWerff 2022 | C |  |  | x |  |  |  |  |  |  |  | 1 |
| External ventricular and lumbar drain-related meningitis | Automated surveillance | vanMourik 2011 | A, C |  |  |  |  |  |  |  | x | x |  | 1 |
| MRSA | Automated surveillance | Peterson 2012 | C |  |  | x |  |  |  |  |  |  |  | 1 |
| PJI | Automated surveillance | Fu 2021 | B |  | x |  |  |  |  |  |  |  |  | 1 |
| Neurological | Prediction modelling | Cheng, Bai 2023 | A, B, C |  | x | x | x |  |  |  |  | x |  | 1 |

#

# Appendix F: Overview of included articles, guidelines and definitions

**Table S16:** Type of infection and guidelines used as definition from the literature review. ACS NSQIP = American College of Surgeons National Surgical Quality Improvement program, CDC = Centers for Disease Control and Prevention, HAI = hospital-acquired Infections, PREZIES = PREventie van ZIEkenhuisinfecties door surveillance (prevention of hospital infections through surveillance, a Dutch initiative), SSISS = Surgical Site Infection Surveillance Service, NHSN = National Healthcare Safety Network (CDC), IDSA = Infectious Diseases Society of America, ACCP/SCCM = American College of Chest Physicians/Society of Critical Care Medicine, STS = Society of Thoracic Surgeons, VASQIP = Veterans Affairs Surgical Quality Improvement Program, WHO = world health organization, EAU = European Association of Urology, ECDC = European Centre for Disease Prevention and Control, PHE = Public Health England, NICE = The National Institute for Health and Care Excellence.

| **Type of infection** | **Guidelines identified in systematic search on prediction models**** | **Studies[prediction models] using alternative definition (first authors)** | **Automated surveillance methods (first authors)** |
| --- | --- | --- | --- |
| HAI/General | WHO, ECDC |  | Ehrentraut et al., Tvardik et al., Sakji et al. |
| Pneumonia | (E)CDC/ACS NSQIP | Kinlin et al. | Blacky et al., Bouzbid et al., Cato et al., Tvardik et al, FitzHenry et al., Colborn et al., Stern et al. |
| (Superficial/deep/  organ space) Surgical site infection (SSIs) | ACS NSQIP/NHSN/  (E)CDC/PREZIES/  SSISS/NNIS* | Daneman et al^.,^ Weller et al.,Crispin et al., Martin et al. | Campillo-Gimenez et al, Cato et al., FitzHenry et al.^,^ Leclère et al., Leth et al., Suzuki et al., Thirukumaran et al., Tvardik et al.. Colborn et al. |
| Anastomotic leakage and abdominal infections | Study group of rectal cancer AL | Stidham et al, Miyakita et al, McKenna et al., Nudel et al,  Kawai et al., Lin 2022 et al., Shi et al., van Kooten et al. |  |
| Bloodstream infections (including central venous catheter-related infection, and sepsis) | ACS NSQIP, ACCP/SCCM (sepsis-3 criteria) |  | Blacky et al., Bouzbid et al., Cato et al., Bouam et al., Leal (2010) et al., Leal (2016) et al., FitzHenry et al., Lin et al., Redder et al., Tvardik et al., Valik et al., Venable et al., Woeltje et al., Colborn et al. |
| UTI (including catheter related UTIs) | (E)CDC/ASC NSQIP | Cheng, Liu et al. 2022 | Blacky et al., Bouzbid et al., Cato et al., Bouam et al., Branch-Eliman et al^.^, Choudhuri et al., FitzHenry et al., Leth et al., Redder et al., Tvardik et al., van der Werff et al., Venable et al., Wald et al., Colborn et al. |
| Other: Periprosthetic joint infection, C. Difficile, MRSA, External ventricular and lumbar drain-related meningitis, Neurological |  | Cheng, Bai et al. 2023. | Fu et al., Dubberke et al., Peterson et al., Van Mourik et al., Clostridium Difficile et al. |

* ACS NSQIP/NHSN/(E)CDC/PREZIES/SSISS/NNIS use a similar definition, based on CDC guidelines.
** See Supplementary material for data extraction tables

# Appendix G: Performance of surveillance algorithms

**Table S17:** Performance reported per surveillance algorithm.

| **Type of infection** | **Reference** | **Type of algorithm** | **Sensitivity** | **Specificity** | **PPV** | **NPV** | **Accuracy** | **Other** |
| --- | --- | --- | --- | --- | --- | --- | --- | --- |
| HAI/General | Ehrentraut | B | 0.937 |  | 0.797 |  |  | F1 score: 0.857 |
| HAI/General | Sakji | B | 0.58 |  | 0.62 |  |  | F: 0.59 |
| HAI/General | Tvardik | B | 0.839 (0.717 - 0.924) | 0.842 (0.721 - 0.925) |  |  | 0.84 |  |
| Pneumoniae | Blacky | A, C | 0.903 | 1 | 1 | 0.957 | 0.97 |  |
| Pneumoniae | Bouzbid | A, C | 0.993 (98.2-100) | 0.56 (54-59.6) | 0.347 (31.5-38.1) | 0.997 (99.3-100) |  |  |
| Pneumoniae | Cato | A, C |  |  |  |  |  |  |
| Pneumoniae | FitzHenry | B | 0.80 (0.76-0.83) | 0.90 (0.89-0.91) |  |  |  |  |
| Pneumoniae | Tvardik | B | 0.839 (0.717 - 0.924) | 0.842 (0.721 - 0.925) |  |  |  |  |
| Pneumoniae | Colborn | A | 0.87 | 0.95 | 0.10 | 0.99 | 0.95 | AUC: 0.96 |
| Pneumoniae | Stern | A, D | 0.71 |  | 0.48 | 0.90 |  |  |
| SSI | Campillo-Gimenez | A, B | 0.846 |  | 0.048 |  |  | F-score: 0.091 |
| SSI | Cato | A, C |  |  |  |  |  |  |
| SSI | FitzHenry | B | 0.77 (0.74-0.80) | 0.63 (0.61-0.64) |  |  |  |  |
| SSI | Leclere | A, C | 0.90 (0.85-0.96) | 0.91 (0.90-0.92) | 0.21 (0.17-0.25) | 0.997 (0.996-0.999) |  |  |
| SSI | Leth | A, C | 0.77 (0.63-0.88) | 0.995 (0.99-0.998) | 0.84 (0.70-0.93) | 0.99 (0.99-0.996) |  |  |
| SSI | Suzuki | A, B, C |  |  |  |  |  | AUC: 0.96, 0.97 |
| SSI | Tvardik | B | 0.839 (0.717 - 0.924) | 0.842 (0.721 - 0.925) |  |  | 0.84 |  |
| SSI | Thirukumaran | B | 0.97 |  | 0.97 |  |  | AUC: 0.96, F1: 0.97 |
| SSI | Colborn | A | 0.89 | 0.82 | 0.13 | 0.99 | 0.82 | AUC: 0.91 |
| UTI | Bouam 2003 | C | 0.91 (0.89-0.93) | 0.91 (0.89-0.93) | 0.88 (0.85-0.91) | 0.93 (0.91-0.95) |  | Kappa: 0.81 |
| UTI | Bouzbid 2011 | A, C | 0.99 (0.98-1.00) | 0.57 (0.54-0.60) | 0.35 (0.32-0.38) | 0.997 (0.99-1.00) |  |  |
| UTI | Branch-Elliman | A, B | 0.65 | 0.996 | 0.542 | 0.997 |  |  |
| UTI | Cato 2017 | A, C |  |  |  |  |  |  |
| UTI | Choudhuri 2011 | A, C | 0.864 | 0.938 | 0.85 | 0.944 |  |  |
| UTI | FitzHenry 2013 | B | 0.95 (0.93-0.96) | 0.80 (0.79-0.81) |  |  |  |  |
| UTI | Leth 2010 | A, C | 0.90 (0.77-0.97) | 0.98 (0.97-0.98) | 0.54 (0.43-0.66) | 0.997 (0.99-0.999) |  |  |
| UTI | Redder 2015 | A, C | 0.94 (0.88-1.0) | 0.99 (0.99-1.0) | 0.95 (0.90-1.0) | 0.99 (0.98-1.0) |  |  |
| UTI | Tvardik 2018 | B | 0.839 (0.717 - 0.924) | 0.842 (0.721 - 0.925) |  |  | 0.84 |  |
| UTI | vanderWerff 2021 | A, B, C | 0.675 (0.600-0.742) | 0.935 (0.913-0.951) | 0.719 (0.643-0.784) | 0.921 (0.898-0.939) |  |  |
| UTI | Venable 2013 | A, B | 0.5 | 0.979 |  |  |  |  |
| UTI | Wald 2014 | A, C | 0.8 | 0.99 | 0.69 | 0.99 |  | kappa = 0.71 agreement = 0.98 |
| UTI | Colborn | A | 0.90 | 0.90 | 0.12 | 0.99 | 0.90 | AUC: 0.93 |
| Bloodstream | Blacky 2011 | A, C | 0.903 | 1 | 1 | 0.957 | 0.97 |  |
| Bloodstream | Bouam 2003 | C | 0.91 (0.89-0.93) | 0.91 (0.89-0.93) | 0.88 (0.85-0.91) | 0.93 (0.91-0.95) |  | Kappa: 0.81 |
| Bloodstream | Bouzbid 2011 | A, C | 0.99 (0.98-1.00) | 0.57 (0.54-0.60) | 0.35 (0.32-0.38) | 0.997 (0.99-1.00) |  |  |
| Bloodstream | Cato 2017 | A, C |  |  |  |  |  |  |
| Bloodstream | FitzHenry 2013 | B | 0.88 (0.84-0.91) | 0.92 (0.91-0.93) |  |  |  |  |
| Bloodstream | Leal 2010 | C |  |  |  |  |  | Agreement 85% K = 0.78 (SE 0.04) |
| Bloodstream | Leal 2016 | C |  |  |  |  |  | 85.5% agreement k = 0.78 (0.75-0.8) |
| Bloodstream | Lin 2014 | C |  |  |  |  |  | Kappa = 0.58 (0.52-0.64) |
| Bloodstream | Redder 2015 | A, C | 1.0 (1.0-1.0) | 1.0 (0.99-1.0 | 0.88 (0.73-1.0) | 1.0 (1.0-1.0) |  |  |
| Bloodstream | Tvardik 2018 | B | 0.839 (0.717 - 0.924) | 0.842 (0.721 - 0.925) |  |  | 0.84 |  |
| Bloodstream | Valik 2020 | A, C | 0.887 (CI: 0.799 to 0.964) | 0.985 (0.978 to 0.991) | 0.881 (0.833 to 0.926) | 0.986 (0.973 to 0.996) |  |  |
| Bloodstream | Venable 2013 | A, B | 0.6 | 0.988 |  |  |  |  |
| Bloodstream | Woeltje 2011 | A, C | 0.952 (0.885–0.982) | 0.975 (0.954–0.987) | 0.900 (0.821–0.947) | 0.992 (0.972–0.996) |  | Kappa = 0.908 Pearson's correlation coefficient = 0.908 |
| Bloodstream | Colborn | A | 0.93 | 0.89 | 0.15 | 0.99 | 0.89 | AUC: 0.95 |
| Other | Dubberke 2012 | C | 0.92 | 0.99 |  |  |  | k-value 0.90 |
| Other | vanderWerff 2022 | C | 1.00 | 1.00 | 0.96 | 1.00 |  | AUC: 0.999 |
| Other | vanMourik 2011 | A, C | 0.98 (0.88-0.999) | 0.879 (0.846-0.908) | 0.569 (0.508-0.679) | 0.999 (0.986-0.999) |  |  |
| Other | Peterson 2012 | C |  |  |  |  |  | Correlation coefficient (0.79-0.98) |
| Other | Fu 2021 | B | 0.937 | 0.984 |  |  |  | F1 score: 0.911 |

# References

1. Adegboyega TO, Borgert AJ, Lambert PJ, Jarman BT. Applying the National Surgical Quality Improvement Program risk calculator to patients undergoing colorectal surgery: theory vs reality. Am J Surg. 2017;213(1):30-5. PMID: 27424043. doi: 10.1016/j.amjsurg.2016.04.011.

2. Alzahrani SM, Ko CS, Yoo MW. Validation of the ACS NSQIP surgical risk calculator for patients with early gastric cancer treated with laparoscopic gastrectomy. Journal of Gastric Cancer. 2020;20(3):267-76. PMID: 2005146773. doi: <http://dx.doi.org/10.5230/jgc.2020.20.e27>.

3. Armstrong EA, Beal EW, Lopez-Aguiar AG, Poultsides G, Cannon JG, Rocha F, et al. Evaluating the ACS-NSQIP Risk Calculator in Primary GI Neuroendocrine Tumor: Results from the United States Neuroendocrine Tumor Study Group. Am Surg. 2019;85(12):1334-40.

4. Arozullah AM, Khuri SF, Henderson WG, Daley J. Development and validation of a multifactorial risk index for predicting postoperative pneumonia after major noncardiac surgery. Ann Intern Med. 2001;135(10):847-57. PMID: 11712875. doi: 10.7326/0003-4819-135-10-200111200-00005.

5. Basta MN, Bauder AR, Kovach SJ, Fischer JP. Assessing the predictive accuracy of the American College of Surgeons National Surgical Quality Improvement Project Surgical Risk Calculator in open ventral hernia repair. Am J Surg. 2016;212(2):272-81. doi: 10.1016/j.amjsurg.2016.01.034.

6. Bekelis K, Desai A, Bakhoum SF, Missios S. A predictive model of complications after spine surgery: the National Surgical Quality Improvement Program (NSQIP) 2005-2010. Spine J. 2014;14(7):1247-55. doi: 10.1016/j.spinee.2013.08.009.

7. Benk MS, Olcucuoğlu E, Kaya İ O. Evaluation of complications after laparoscopic and open appendectomy by the American College of Surgeons National Surgical Quality Improvement Program surgical risk calculator. Ulus Travma Acil Cerrahi Derg. 2022;28(4):418-27. doi: 10.14744/tjtes.2020.45808.

8. Berger RL, Hicks SC, Davila JA, Li LT, Clapp ML, Berger DH, et al. Development and validation of a risk stratification score for identifying patients at increased risk of surgical site infection following open ventral hernia repair. Journal of Surgical Research Conference: 8th Annual Academic Surgical Congress of the Association for Academic Surgery, AAS and the Society of University Surgeons, SUS New Orleans, LA United States Conference Publication:. 2013;179(2). PMID: 70973062.

9. Bergquist JR, Thiels CA, Etzioni DA, Habermann EB, Cima RR. Failure of Colorectal Surgical Site Infection Predictive Models Applied to an Independent Dataset: Do They Add Value or Just Confusion? J Am Coll Surg. 2016;222(4):431-8. doi: 10.1016/j.jamcollsurg.2015.12.034.

10. Bertsimas D, Dunn J, Velmahos GC, Kaafarani HMA. Surgical Risk Is Not Linear: Derivation and Validation of a Novel, User-friendly, and Machine-learning-based Predictive OpTimal Trees in Emergency Surgery Risk (POTTER) Calculator. Ann Surg. 2018;268(4):574-83. doi: 10.1097/SLA.0000000000002956.

11. Bonde A, Varadarajan KM, Bonde N, Troelsen A, Muratoglu OK, Malchau H, et al. Assessing the utility of deep neural networks in predicting postoperative surgical complications: a retrospective study. Lancet Digit Health. 2021;3(8):e471-e85. PMID: 34215564. doi: 10.1016/s2589-7500(21)00084-4.

12. Bronheim RS, Oermann EK, Bronheim DS, Caridi JM. Revised Cardiac Risk Index versus ASA Status as a Predictor for Noncardiac Events After Posterior Lumbar Decompression. World Neurosurg. 2018;120:e1175-e84. doi: 10.1016/j.wneu.2018.09.028.

13. Cabrera A, Bouterse A, Nelson M, Razzouk J, Ramos O, Chung D, et al. Use of random forest machine learning algorithm to predict short term outcomes following posterior cervical decompression with instrumented fusion. J Clin Neurosci. 2023;107:167-71. doi: 10.1016/j.jocn.2022.10.029.

14. Chen LF, Anderson DJ, Kaye KS, Sexton DJ. Validating a 3-point prediction rule for surgical site infection after coronary artery bypass surgery. Infect Control Hosp Epidemiol. 2010;31(1):64-8. doi: 10.1086/649019.

15. Chen Z, Zhong M, Xu Z, Ye Q, Xie W, Gao S, et al. Development and Validation of a Nomogram Based on Geriatric Nutritional Risk Index to Predict Surgical Site Infection Among Gynecologic Oncology Patients. Front Nutr. 2022;9:864761. doi: 10.3389/fnut.2022.864761.

16. Cheng X, Liu Y, Wang W, Yan J, Lei X, Wu H, et al. Preoperative Risk Factor Analysis and Dynamic Online Nomogram Development for Early Infections Following Primary Hip Arthroplasty in Geriatric Patients with Hip Fracture. Clin Interv Aging. 2022;17:1873-83. doi: 10.2147/CIA.S392393.

17. Cheng Y, Chen Y, Hou X, Yu J, Wen H, Dai J, et al. Development of a Nomogram for Predicting Surgical Site Infection in Patients with Resected Lung Neoplasm Undergoing Minimally Invasive Surgery. Surg Infect (Larchmt). 2022;23(8):754-62. doi: 10.1089/sur.2022.166.

18. Cheng L, Liu J, Lian L, Duan W, Guan J, Wang K, et al. Predicting deep surgical site infection in patients receiving open posterior instrumented thoracolumbar surgery--- A-DOUBLE-SSI risk score: a large retrospective multicenter cohort study in China. Int J Surg. 2023. doi: 10.1097/JS9.0000000000000461.

19. Cheng L, Bai W, Song P, Zhou L, Li Z, Gao L, et al. Development and Validation of a Nomograph Model for Post-Operative Central Nervous System Infection after Craniocerebral Surgery. Diagnostics (Basel). 2023;13(13). doi: 10.3390/diagnostics13132207.

20. Chudgar N, Yan S, Hsu M, Tan KS, Gray KD, Nobel T, et al. External validation of surgical risk preoperative assessment system (SURPAS) in pulmonary resection. Ann Thorac Surg. 2020. doi: 10.1016/j.athoracsur.2020.08.023.

21. Chudgar NP, Yan S, Hsu M, Tan KS, Gray KD, Molena D, et al. Performance Comparison Between SURPAS and ACS NSQIP Surgical Risk Calculator in Pulmonary Resection. Ann Thorac Surg. 2020. doi: 10.1016/j.athoracsur.2020.08.021.

22. Crispin A, Klinger C, Rieger A, Strahwald B, Lehmann K, Buhr HJ, et al. The DGAV risk calculator: development and validation of statistical models for a web-based instrument predicting complications of colorectal cancer surgery. Int J Colorectal Dis. 2017;32(10):1385-97. doi: 10.1007/s00384-017-2869-6.

23. Daneman N, Simor AE, Redelmeier DA. Validation of a modified version of the national nosocomial infections surveillance system risk index for health services research. Infect Control Hosp Epidemiol. 2009;30(6):563-9. doi: 10.1086/597523.

24. Dave A, Beal EW, Lopez-Aguiar AG, Poultsides G, Makris E, Rocha FG, et al. Evaluating the ACS NSQIP Risk Calculator in Primary Pancreatic Neuroendocrine Tumor: Results from the US Neuroendocrine Tumor Study Group. J Gastrointest Surg. 2019;23(11):2225-31. doi: 10.1007/s11605-019-04120-4.

25. Deek RP, Lee IOK, van Essen P, Crittenden T, Dean NR. Predicted versus actual complications in Australian women undergoing post-mastectomy breast reconstruction: a retrospective cohort study using the BRA Score tool. J Plast Reconstr Aesthet Surg. 2021;74(12):3324-34. PMID: 34253489. doi: 10.1016/j.bjps.2021.05.039.

26. Donnally CJ, Henstenburg JM, Pezzulo JD, Farronato D, Patel PD, Sherman M, et al. Increased Surgical Site Subcutaneous Fat Thickness Is Associated with Infection after Posterior Cervical Fusion. Surg Infect (Larchmt). 2022;23(4):364-71. doi: 10.1089/sur.2021.271.

27. Edelstein AI, Kwasny MJ, Suleiman LI, Khakhkhar RH, Moore MA, Beal MD, et al. Can the American College of Surgeons Risk Calculator Predict 30-Day Complications After Knee and Hip Arthroplasty? J Arthroplasty. 2015;30(9 Suppl):5-10. doi: 10.1016/j.arth.2015.01.057.

28. El Asmar A, Hafez K, Fauconnier P, Moreau M, Dal Lago L, Pepersack T, et al. The efficacy of the American College of Surgeons Surgical Risk Calculator in the prediction of postoperative complications in oncogeriatric patients after curative surgery for abdominal tumors. J Surg Oncol. 2022;126(7):1359-66. doi: 10.1002/jso.27046.

29. El Hechi MW, Maurer LR, Levine J, Zhuo D, El Moheb M, Velmahos GC, et al. Validation of the Artificial Intelligence-Based Predictive Optimal Trees in Emergency Surgery Risk (POTTER) Calculator in Emergency General Surgery and Emergency Laparotomy Patients. J Am Coll Surg. 2021. doi: 10.1016/j.jamcollsurg.2021.02.009.

30. El Moheb M, Gebran A, Maurer LR, Naar L, El Hechi M, Breen K, et al. Artificial Intelligence versus Surgeon Gestalt in Predicting Risk of Emergency General Surgery. J Trauma Acute Care Surg. 2023. doi: 10.1097/TA.0000000000004030.

31. Figuerola-Tejerina A, Bustamante E, Tamayo E, Mestres CA, Bustamante-Munguira J. Ability to predict the development of surgical site infection in cardiac surgery using the Australian Clinical Risk Index versus the National Nosocomial Infections Surveillance-derived Risk Index. Eur J Clin Microbiol Infect Dis. 2017;36(6):1041-6. PMID: 28105547. doi: 10.1007/s10096-016-2889-0.

32. Gervaz P, Bandiera-Clerc C, Buchs NC, Eisenring MC, Troillet N, Perneger T, et al. Scoring system to predict the risk of surgical-site infection after colorectal resection. Br J Surg. 2012;99(4):589-95. PMID: 22231649. doi: 10.1002/bjs.8656.

33. Goulart A, Ferreira C, Estrada A, Nogueira F, Martins S, Mesquita-Rodrigues A, et al. Early Inflammatory Biomarkers as Predictive Factors for Freedom from Infection after Colorectal Cancer Surgery: A Prospective Cohort Study. Surg Infect (Larchmt). 2018;19(4):446-50. doi: 10.1089/sur.2017.294.

34. Gowd AK, Agarwalla A, Amin NH, Romeo AA, Nicholson GP, Verma NN, et al. Construct validation of machine learning in the prediction of short-term postoperative complications following total shoulder arthroplasty. J Shoulder Elbow Surg. 2019;28(12):e410-e21. doi: 10.1016/j.jse.2019.05.017.

35. Gowd AK, O'Neill CN, Barghi A, O'Gara TJ, Carmouche JJ. Feasibility of Machine Learning in the Prediction of Short-Term Outcomes Following Anterior Cervical Discectomy and Fusion. World Neurosurg. 2022;168:e223-e32. doi: 10.1016/j.wneu.2022.09.090.

36. Grant R, Aupee M, Buchs NC, Cooper K, Eisenring MC, Lamagni T, et al. Performance of surgical site infection risk prediction models in colorectal surgery: external validity assessment from three European national surveillance networks. Infect Control Hosp Epidemiol. 2019;40(9):983-90. doi: 10.1017/ice.2019.163.

37. Gupta H, Gupta PK, Schuller D, Fang X, Miller WJ, Modrykamien A, et al. Development and validation of a risk calculator for predicting postoperative pneumonia. Mayo Clin Proc. 2013;88(11):1241-9. doi: 10.1016/j.mayocp.2013.06.027.

38. Hamade S, Alshiek J, Javadian P, Ahmed S, McLeod FN, Shobeiri SA. Evaluation of the American College of Surgeons National Surgical Quality Improvement Program Risk Calculator to predict outcomes after hysterectomies. Int J Gynaecol Obstet. 2022;158(3):714-21. doi: 10.1002/ijgo.14075.

39. Han K, Lee JM, Achanta A, Kongkaewpaisan N, Kongwibulwut M, Eid AI, et al. Emergency Surgery Score Accurately Predicts the Risk of Post-Operative Infection in Emergency General Surgery. Surg Infect (Larchmt). 2019;20(1):4-9. PMID: 30272533. doi: 10.1089/sur.2018.101.

40. Harris AHS, Trickey AW, Eddington HS, Seib CD, Kamal RN, Kuo AC, et al. A Tool to Estimate Risk of 30-day Mortality and Complications After Hip Fracture Surgery: Accurate Enough for Some but Not All Purposes? A Study From the ACS-NSQIP Database. Clin Orthop Relat Res. 2022;480(12):2335-46. doi: 10.1097/CORR.0000000000002294.

41. Hedrick TL, Sawyer RG, Friel CM, Stukenborg GJ. A method for estimating the risk of surgical site infection in patients with abdominal colorectal procedures. Dis Colon Rectum. 2013;56(5):627-37. doi: 10.1097/DCR.0b013e318279a93e.

42. Henderson WG, Bronsert MR, Hammermeister KE, Lambert-Kerzner A, Meguid RA. Refining the predictive variables in the "Surgical Risk Preoperative Assessment System" (SURPAS): a descriptive analysis. Patient Saf Surg. 2019;13:28. doi: 10.1186/s13037-019-0208-2.

43. Hu JS, Huang CB, Mao SM, Fang KH, Wu ZY, Zhao YM. Development of a nomogram to predict surgical site infection after closed comminuted calcaneal fracture. BMC Surg. 2022;22(1):313. doi: 10.1186/s12893-022-01735-4.

44. Jonczyk MM, Fisher CS, Babbitt R, Paulus JK, Freund KM, Czerniecki B, et al. Surgical Predictive Model for Breast Cancer Patients Assessing Acute Postoperative Complications: The Breast Cancer Surgery Risk Calculator. Ann Surg Oncol. 2021. doi: 10.1245/s10434-021-09710-8.

45. Kawai K, Hirakawa S, Tachimori H, Oshikiri T, Miyata H, Kakeji Y, et al. Updating the predictive models for mortality and morbidity after low anterior resection based on the National Clinical Database. Dig Surg. 2023. doi: 10.1159/000531370.

46. Kikuchi H, Miyata H, Konno H, Kamiya K, Tomotaki A, Gotoh M, et al. Development and external validation of preoperative risk models for operative morbidities after total gastrectomy using a Japanese web-based nationwide registry. Gastric Cancer. 2017;20(6):987-97. doi: 10.1007/s10120-017-0706-9.

47. Kinlin LM, Kirchner C, Zhang H, Daley J, Fisman DN. Derivation and validation of a clinical prediction rule for nosocomial pneumonia after coronary artery bypass graft surgery. Clinical Infectious Diseases. 2010;50(4):493-501. PMID: 358220108. doi: <http://dx.doi.org/10.1086/649925>.

48. Kirmani BH, Mazhar K, Saleh HZ, Ward AN, Shaw M, Fabri BM, et al. External validity of the Society of Thoracic Surgeons risk stratification tool for deep sternal wound infection after cardiac surgery in a UK population. Interact Cardiovasc Thorac Surg. 2013;17(3):479-84. PMID: 23760358. doi: 10.1093/icvts/ivt222.

49. Klemencsics I, Lazary A, Szoverfi Z, Bozsodi A, Eltes P, Varga PP. Risk factors for surgical site infection in elective routine degenerative lumbar surgeries. Spine J. 2016;16(11):1377-83. doi: 10.1016/j.spinee.2016.08.018.

50. Lee T, Hwang EJ, Park CM, Goo JM. Deep Learning-Based Computer-Aided Detection System for Preoperative Chest Radiographs to Predict Postoperative Pneumonia. Acad Radiol. 2023. doi: 10.1016/j.acra.2023.02.016.

51. Leekha S, Lahr BD, Thompson RL, Sampathkumar P, Duncan AA, Orenstein R. Preoperative risk prediction of surgical site infection requiring hospitalization or reoperation in patients undergoing vascular surgery. J Vasc Surg. 2016;64(1):177-84. doi: 10.1016/j.jvs.2016.01.029.

52. Li L, Ding J, Han J, Wu H. A nomogram prediction of postoperative surgical site infections in patients with perihilar cholangiocarcinoma. Medicine (Baltimore). 2017;96(25):e7198. doi: 10.1097/MD.0000000000007198.

53. Lian J, Wang Y, Yan X, Xu G, Jia M, Yang J, et al. Development and validation of a nomogram to predict the risk of surgical site infection within 1 month after transforaminal lumbar interbody fusion. J Orthop Surg Res. 2023;18(1):105. doi: 10.1186/s13018-023-03550-w.

54. Liang MK, Goodenough CJ, Martindale RG, Roth JS, Kao LS. External validation of the ventral hernia risk score for prediction of surgical site infections. Surg Infect (Larchmt). 2015;16(1):36-40. doi: 10.1089/sur.2014.115.

55. Lin V, Tsouchnika A, Allakhverdiiev E, Rosen AW, Gögenur M, Clausen JSR, et al. Training prediction models for individual risk assessment of postoperative complications after surgery for colorectal cancer. Tech Coloproctol. 2022;26(8):665-75. doi: 10.1007/s10151-022-02624-x.

56. Liu X, Lei S, Wei Q, Wang Y, Liang H, Chen L. Machine Learning-based Correlation Study between Perioperative Immunonutritional Index and Postoperative Anastomotic Leakage in Patients with Gastric Cancer. Int J Med Sci. 2022;19(7):1173-83. doi: 10.7150/ijms.72195.

57. Liu WC, Ying H, Liao WJ, Li MP, Zhang Y, Luo K, et al. Using Preoperative and Intraoperative Factors to Predict the Risk of Surgical Site Infections After Lumbar Spinal Surgery: A Machine Learning-Based Study. World Neurosurg. 2022;162:e553-e60. doi: 10.1016/j.wneu.2022.03.060.

58. Lone Z, Hall S, Terakawa T, Ahmed YE, Elsayed AS, Aldhaam N, et al. Accuracy of American College of Surgeons National Surgical Quality Improvement Program Universal Surgical Risk Calculator in Predicting Complications Following Robot-Assisted Radical Cystectomy at a National Comprehensive Cancer Center. J Endourol. 2019;33(5):383-8. doi: 10.1089/end.2019.0093.

59. Long AM, Hildreth AN, Davis PT, Ur R, Badger AT, Miller PR. Evaluation of the Performance of ACS NSQIP Surgical Risk Calculator in Emergency General Surgery Patients. Am Surg. 2020;86(2):83-9.

60. Mamlook REA, Wells LJ, Sawyer R. Machine-learning models for predicting surgical site infections using patient pre-operative risk and surgical procedure factors. Am J Infect Control. 2023;51(5):544-50. doi: 10.1016/j.ajic.2022.08.013.

61. Mannas MP, Lee T, Forbes CM, Hong T, Bisaillon A, Gleave ME, et al. Predicting complications following radical cystectomy with the ACS NSQIP universal surgical risk calculator. World J Urol. 2020;38(5):1215-20. doi: 10.1007/s00345-019-02915-3.

62. Martin S, Turner E, Nguyen A, Thornton B, Nazerali RS. An Evaluation of the Utility of the Breast Reconstruction Risk Assessment Score Risk Model in Prepectoral Tissue Expander Breast Reconstruction. Ann Plast Surg. 2020;84(5S Suppl 4):S318-S22. doi: 10.1097/SAP.0000000000002320.

63. Maruna P, Kunstyr J, Plocova KM, Mlejnsky F, Hubacek J, Klein AA, et al. Predictors of infection after pulmonary endarterectomy for chronic thrombo-embolic pulmonary hypertension. Eur J Cardiothorac Surg. 2011;39(2):195-200. doi: 10.1016/j.ejcts.2010.05.018.

64. Maurer LR, Chetlur P, Zhuo D, El Hechi M, Velmahos GC, Dunn J, et al. Validation of the AI-based Predictive OpTimal Trees in Emergency Surgery Risk (POTTER) Calculator in Patients 65 Years and Older. Ann Surg. 2020;Publish Ahead of Print. doi: 10.1097/SLA.0000000000004714.

65. McCarthy MH, Singh P, Maslak J, Nayak R, Jenkins TJ, Hsu WK, et al. Can the American College of Surgeons Risk Calculator Predict 30-Day Complications After Cervical Spine Surgery? Clin Spine Surg. 2019;32(9):357-62. doi: 10.1097/BSD.0000000000000890.

66. McKenna NP, Bews KA, Cima RR, Crowson CS, Habermann EB. Development of a Risk Score to Predict Anastomotic Leak After Left-Sided Colectomy: Which Patients Warrant Diversion? J Gastrointest Surg. 2020;24(1):132-43. doi: 10.1007/s11605-019-04293-y.

67. Meguid RA, Bronsert MR, Juarez-Colunga E, Hammermeister KE, Henderson WG. Surgical Risk Preoperative Assessment System (SURPAS): II. Parsimonious Risk Models for Postoperative Adverse Outcomes Addressing Need for Laboratory Variables and Surgeon Specialty-specific Models. Ann Surg. 2016;264(1):10-22. doi: 10.1097/SLA.0000000000001677.

68. Menezes AS, Fernandes A, Rodrigues JR, Salome C, Machado F, Antunes L, et al. Optimizing classical risk scores to predict complications in head and neck surgery: a new approach. European Archives of Oto-Rhino-Laryngology. 2021;278(1):191-202. PMID: WOS:000541223800001. doi: 10.1007/s00405-020-06133-1.

69. Mitchell TO, Holihan JL, Askenasy EP, Greenberg JA, Keith JN, Martindale RG, et al. Do risk calculators accurately predict surgical site occurrences? J Surg Res. 2016;203(1):56-63. doi: 10.1016/j.jss.2016.03.040.

70. Miyakita H, Sadahiro S, Saito G, Okada K, Tanaka A, Suzuki T. Risk scores as useful predictors of perioperative complications in patients with rectal cancer who received radical surgery. Int J Clin Oncol. 2017;22(2):324-31. doi: 10.1007/s10147-016-1054-1.

71. Muñoz JL, Ruiz-Tovar J, Miranda E, Berrio DL, Moya P, Gutiérrez M, et al. C-Reactive Protein and Procalcitonin as Early Markers of Septic Complications after Laparoscopic Sleeve Gastrectomy in Morbidly Obese Patients Within an Enhanced Recovery After Surgery Program. J Am Coll Surg. 2016;222(5):831-7. doi: 10.1016/j.jamcollsurg.2016.01.059.

72. Narain AS, Kitto AZ, Braun B, Poorman MJ, Curtin P, Slavin J, et al. Does the ACS NSQIP Surgical Risk Calculator Accurately Predict Complications Rates After Anterior Lumbar Interbody Fusion Procedures? Spine (Phila Pa 1976). 2020;Publish Ahead of Print. doi: 10.1097/BRS.0000000000003893.

73. Neumayer L, Hosokawa P, Itani K, El-Tamer M, Henderson WG, Khuri SF. Multivariable predictors of postoperative surgical site infection after general and vascular surgery: results from the patient safety in surgery study. J Am Coll Surg. 2007;204(6):1178-87. doi: 10.1016/j.jamcollsurg.2007.03.022.

74. Nudel J, Bishara AM, de Geus SWL, Patil P, Srinivasan J, Hess DT, et al. Development and validation of machine learning models to predict gastrointestinal leak and venous thromboembolism after weight loss surgery: an analysis of the MBSAQIP database. Surg Endosc. 2021;35(1):182-91. doi: 10.1007/s00464-020-07378-x.

75. Ohkura Y, Miyata H, Konno H, Udagawa H, Ueno M, Shindoh J, et al. Development of a model predicting the risk of eight major postoperative complications after esophagectomy based on 10 826 cases in the Japan National Clinical Database. J Surg Oncol. 2019. doi: 10.1002/jso.25800.

76. Ohno Y, Mazaki J, Udo R, Tago T, Kasahara K, Enomoto M, et al. Preliminary Evaluation of a Novel Artificial Intelligence-based Prediction Model for Surgical Site Infection in Colon Cancer. Cancer Diagn Progn. 2022;2(6):691-6. doi: 10.21873/cdp.10161.

77. Orlandi BMM, Mejia OAV, Sorio JL, de Barros ESP, Oliveira MAP, Nakazone MA, et al. Performance of a novel risk model for deep sternal wound infection after coronary artery bypass grafting. Sci Rep. 2022;12(1):15177. doi: 10.1038/s41598-022-19473-1.

78. Poruk KE, Lin JA, Cooper MA, He J, Makary MA, Hirose K, et al. A novel, validated risk score to predict surgical site infection after pancreaticoduodenectomy. HPB (Oxford). 2016;18(11):893-9. doi: 10.1016/j.hpb.2016.07.011.

79. Poruk KE, Hicks CW, Trent Magruder J, Rodriguez-Unda N, Burce KK, Azoury SC, et al. Creation of a novel risk score for surgical site infection and occurrence after ventral hernia repair. Hernia. 2016:1‐9. PMID: CN-01287557. doi: 10.1007/s10029-016-1547-x.

80. Prasad KG, Nelson BG, Deig CR, Schneider AL, Moore MG. ACS NSQIP Risk Calculator: An Accurate Predictor of Complications in Major Head and Neck Surgery? Otolaryngol Head Neck Surg. 2016;155(5):740-2. doi: 10.1177/0194599816655976.

81. Ravindran K, Escobar D, Gautam S, Puri R, Awad Z. Assessment of the American College of Surgeons National Surgical Quality Improvement Program Calculator in Predicting Outcomes and Length of Stay After Ivor Lewis Esophagectomy: A Single-Center Experience. Journal of Surgical Research. 2020;255:355-60. PMID: 2006857936. doi: <http://dx.doi.org/10.1016/j.jss.2020.05.080>.

82. Ren Y, Loftus TJ, Datta S, Ruppert MM, Guan Z, Miao S, et al. Performance of a Machine Learning Algorithm Using Electronic Health Record Data to Predict Postoperative Complications and Report on a Mobile Platform. JAMA Netw Open. 2022;5(5):e2211973. doi: 10.1001/jamanetworkopen.2022.11973.

83. Rencuzogullari A, Benlice C, Valente M, Abbas MA, Remzi FH, Gorgun E. Predictors of Anastomotic Leak in Elderly Patients After Colectomy: Nomogram-Based Assessment From the American College of Surgeons National Surgical Quality Program Procedure-Targeted Cohort. Dis Colon Rectum. 2017;60(5):527-36. PMID: 28383453. doi: 10.1097/dcr.0000000000000789.

84. Rivard C, Nahum R, Slagle E, Duininck M, Isaksson Vogel R, Teoh D. Evaluation of the performance of the ACS NSQIP surgical risk calculator in gynecologic oncology patients undergoing laparotomy. Gynecol Oncol. 2016;141(2):281-6. doi: 10.1016/j.ygyno.2016.02.015.

85. Sangsuwan T, Jamulitrat S, Watcharasin P. Risk adjustment performance between NNIS index and NHSN model for postoperative colorectal surgical site infection: A retrospective cohort study. Ann Med Surg (Lond). 2022;77:103715. doi: 10.1016/j.amsu.2022.103715.

86. Scepanovic MS, Kovacevic B, Cijan V, Antic A, Petrovic Z, Asceric R, et al. C-reactive protein as an early predictor for anastomotic leakage in elective abdominal surgery. Tech Coloproctol. 2013;17(5):541-7. doi: 10.1007/s10151-013-1013-z.

87. Schneider AL, Deig CR, Prasad KG, Nelson BG, Mantravadi AV, Brigance JS, et al. Ability of the National Surgical Quality Improvement Program Risk Calculator to Predict Complications Following Total Laryngectomy. JAMA Otolaryngol Head Neck Surg. 2016;142(10):972-9. PMID: 27467967. doi: 10.1001/jamaoto.2016.1809.

88. Scotton G, Del Zotto G, Bernardi L, Zucca A, Terranova S, Fracon S, et al. Is the ACS-NSQIP Risk Calculator Accurate in Predicting Adverse Postoperative Outcomes in the Emergency Setting? An Italian Single-center Preliminary Study. World J Surg. 2020;44(11):3710-9. doi: 10.1007/s00268-020-05705-w.

89. Scotton G, La Greca A, Lirusso C, Mariani D, Zago M, Chiarugi M, et al. Can the American College of Surgeons NSQIP Surgical Risk Calculator Accurately Predict Adverse Postoperative Outcomes in Emergency Abdominal Surgery? An Italian Multicenter Analysis. J Am Coll Surg. 2023;236(2):387-98. doi: 10.1097/XCS.0000000000000445.

90. Shi J, Wu Z, Wu X, Shan F, Zhang Y, Ying X, et al. Early diagnosis of anastomotic leakage after colorectal cancer surgery using an inflammatory factors-based score system. BJS Open. 2022;6(3). doi: 10.1093/bjsopen/zrac069.

91. Shimizu T, Endo Y, Tabata T, Mori T, Hanasawa K, Tsuchiya M, et al. Diagnostic and predictive value of the silkworm larvae plasma test for postoperative infection following gastrointestinal surgery. Crit Care Med. 2005;33(6):1288-95. doi: 10.1097/01.ccm.0000165810.97971.dd.

92. Stidham RW, Waljee AK, Day NM, Bergmans CL, Zahn KM, Higgins PD, et al. Body fat composition assessment using analytic morphomics predicts infectious complications after bowel resection in Crohn's disease. Inflamm Bowel Dis. 2015;21(6):1306-13. doi: 10.1097/MIB.0000000000000360.

93. Stidham K, Naftchi AF, Spirollari E, Vaserman G, Vazquez S, Das A, et al. Frailty Is Superior to Age for Predicting Readmission, Prolonged Length of Stay, and Wound Infection in Elective Otology Procedures. Otol Neurotol. 2022;43(8):937-43. doi: 10.1097/MAO.0000000000003636.

94. Szender JB, Frederick PJ, Eng KH, Akers SN, Lele SB, Odunsi K. Evaluation of the National Surgical Quality Improvement Program Universal Surgical Risk Calculator for a gynecologic oncology service. Int J Gynecol Cancer. 2015;25(3):512-20. doi: 10.1097/IGC.0000000000000378.

95. Tam S, Dong W, Adelman DM, Weber RS, Lewis CM. Risk-adjustment models in patients undergoing head and neck surgery with reconstruction. Oral Oncol. 2020;111:104917. doi: 10.1016/j.oraloncology.2020.104917.

96. Tierney W, Shah J, Clancy K, Lee MY, Ciolek PJ, Fritz MA, et al. Predictive value of the ACS NSQIP calculator for head and neck reconstruction free tissue transfer. Laryngoscope. 2020;130(3):679-84. doi: 10.1002/lary.28195.

97. Tourani R, Murphree DH, Melton-Meaux G, Wick E, Kor DJ, Simon GJ. The Value of Aggregated High-Resolution Intraoperative Data for Predicting Post-Surgical Infectious Complications at Two Independent Sites. Stud Health Technol Inform. 2019;264:398-402. doi: 10.3233/SHTI190251.

98. Trickey AW, Ding Q, Harris AHS. How Accurate Are the Surgical Risk Preoperative Assessment System (SURPAS) Universal Calculators in Total Joint Arthroplasty? Clin Orthop Relat Res. 2020;478(2):241-51. doi: 10.1097/CORR.0000000000001078.

99. van der Hulst HC, Dekker JWT, Bastiaannet E, van der Bol JM, van den Bos F, Hamaker ME, et al. Validation of the ACS NSQIP surgical risk calculator in older patients with colorectal cancer undergoing elective surgery. J Geriatr Oncol. 2022;13(6):788-95. doi: 10.1016/j.jgo.2022.04.004.

100. van Kooten RT, Bahadoer RR, Ter Buurkes de Vries B, Wouters M, Tollenaar R, Hartgrink HH, et al. Conventional regression analysis and machine learning in prediction of anastomotic leakage and pulmonary complications after esophagogastric cancer surgery. J Surg Oncol. 2022;126(3):490-501. doi: 10.1002/jso.26910.

101. Varetto G, Castagno C, Trucco A, Frola E, Bert F, Scozzari G, et al. Serum Procalcitonin as a Valuable Diagnostic Tool in the Early Detection of Infectious Complications after Open Abdominal Aortic Repair. Ann Vasc Surg. 2016;34:111-8. doi: 10.1016/j.avsg.2016.01.012.

102. Vaziri S, Wilson J, Abbatematteo J, Kubilis P, Chakraborty S, Kshitij K, et al. Predictive performance of the American College of Surgeons universal risk calculator in neurosurgical patients. J Neurosurg. 2018;128(3):942-7. doi: 10.3171/2016.11.JNS161377.

103. Velmahos CS, Paschalidis A, Paranjape CN. The Not-So-Distant Future or Just Hype? Utilizing Machine Learning to Predict 30-Day Post-Operative Complications in Laparoscopic Colectomy Patients. Am Surg. 2023:31348231167397. doi: 10.1177/00031348231167397.

104. Vosler PS, Orsini M, Enepekides DJ, Higgins KM. Predicting complications of major head and neck oncological surgery: an evaluation of the ACS NSQIP surgical risk calculator. J Otolaryngol Head Neck Surg. 2018;47(1):21. PMID: 29566750. doi: 10.1186/s40463-018-0269-8.

105. Vu MM, Ellis MF, Blough JT, Gutowski KA, Kim JYS. Development and Internal Validation of the Abdominoplasty Risk Calculator. Plast Reconstr Surg. 2018;141(1):34e-45e. doi: 10.1097/PRS.0000000000003922.

106. Weller GB, Lovely J, Larson DW, Earnshaw BA, Huebner M. Leveraging electronic health records for predictive modeling of post-surgical complications. Stat Methods Med Res. 2018;27(11):3271-85. doi: 10.1177/0962280217696115.

107. Wherley SD, Chapman GC, Mahajan ST, Hijaz AK, Slopnick EA, Roberts K, et al. Evaluation of the ACS NSQIP surgical risk calculator in patients undergoing pelvic organ prolapse surgery. International Urogynecology Journal. 2020. PMID: 2005252906. doi: <http://dx.doi.org/10.1007/s00192-020-04364-8>.

108. Wingert NC, Gotoff J, Parrilla E, Gotoff R, Hou L, Ghanem E. The ACS NSQIP Risk Calculator Is a Fair Predictor of Acute Periprosthetic Joint Infection. Clin Orthop Relat Res. 2016;474(7):1643-8. doi: 10.1007/s11999-016-4717-3.

109. Winoker JS, Paulucci DJ, Anastos H, Waingankar N, Abaza R, Eun DD, et al. Predicting Complications Following Robot-Assisted Partial Nephrectomy with the ACS NSQIP(®) Universal Surgical Risk Calculator. J Urol. 2017;198(4):803-9. doi: 10.1016/j.juro.2017.04.021.

110. Xun Y, Yang Y, Yu X, Li C, Lu J, Wang S. A preoperative nomogram for sepsis in percutaneous nephrolithotomy treating solitary, unilateral and proximal ureteral stones. PeerJ. 2020;8:e9435. doi: 10.7717/peerj.9435.

111. Yap MKC, Ang KF, Gonzales-Porciuncula LA, Esposo E. Validation of the American College of Surgeons Risk Calculator for preoperative risk stratification. Heart Asia. 2018;10(2):e010993. doi: 10.1136/heartasia-2017-010993.

112. Ying H, Guo BW, Wu HJ, Zhu RP, Liu WC, Zhong HF. Using multiple indicators to predict the risk of surgical site infection after ORIF of tibia fractures: a machine learning based study. Front Cell Infect Microbiol. 2023;13:1206393. doi: 10.3389/fcimb.2023.1206393.

113. Yoshida T, Miyata H, Konno H, Kumamaru H, Tangoku A, Furukita Y, et al. Risk assessment of morbidities after right hemicolectomy based on the National Clinical Database in Japan. Ann Gastroenterol Surg. 2018;2(3):220-30. doi: 10.1002/ags3.12067.

114. Zhang D, Ren J, Arafeh MO, Sawyer RG, Hu Q, Wu X, et al. The Significance of Interleukin-6 in the Early Detection of Surgical Site Infections after Definitive Operation for Gastrointestinal Fistulae. Surg Infect (Larchmt). 2018;19(5):523-8. doi: 10.1089/sur.2017.271.

115. Zhang N, Fan K, Ji H, Ma X, Wu J, Huang Y, et al. Identification of risk factors for infection after mitral valve surgery through machine learning approaches. Front Cardiovasc Med. 2023;10:1050698. doi: 10.3389/fcvm.2023.1050698.

116. Zhou Y, Wang L, Cao A, Luo W, Xu Z, Sheng Z, et al. Modified Frailty Index Combined with a Prognostic Nutritional Index for Predicting Postoperative Complications of Hip Fracture Surgery in Elderly. J Invest Surg. 2022;35(10):1739-46. doi: 10.1080/08941939.2022.2101166.

117. Blacky A, Mandl H, Adlassnig KP, Koller W. Fully Automated Surveillance of Healthcare-Associated Infections with MONI-ICU: A Breakthrough in Clinical Infection Surveillance. Appl Clin Inform. 2011;2(3):365-72. PMID: 23616883. doi: 10.4338/aci-2011-03-ra-0022.

118. Bouam S, Girou E, Brun-Buisson C, Karadimas H, Lepage E. An intranet-based automated system for the surveillance of nosocomial infections: prospective validation compared with physicians' self-reports. Infect Control Hosp Epidemiol. 2003;24(1):51-5. PMID: 12558236. doi: 10.1086/502115.

119. Bouzbid S, Gicquel Q, Gerbier S, Chomarat M, Pradat E, Fabry J, et al. Automated detection of nosocomial infections: evaluation of different strategies in an intensive care unit 2000-2006. J Hosp Infect. 2011;79(1):38-43. PMID: 21742413. doi: 10.1016/j.jhin.2011.05.006.

120. Branch-Elliman W, Strymish J, Kudesia V, Rosen AK, Gupta K. Natural Language Processing for Real-Time Catheter-Associated Urinary Tract Infection Surveillance: Results of a Pilot Implementation Trial. Infect Control Hosp Epidemiol. 2015;36(9):1004-10. PMID: 26022228. doi: 10.1017/ice.2015.122.

121. Campillo-Gimenez B, Garcelon N, Jarno P, Chapplain JM, Cuggia M. Full-text automated detection of surgical site infections secondary to neurosurgery in Rennes, France. Stud Health Technol Inform. 2013;192:572-5. PMID: 23920620.

122. Cato KD, Liu J, Cohen B, Larson E. Electronic Surveillance of Surgical Site Infections. Surg Infect (Larchmt). 2017;18(4):498-502. PMID: 28402721. doi: 10.1089/sur.2016.262.

123. Choudhuri JA, Pergamit RF, Chan JD, Schreuder AB, McNamara E, Lynch JB, et al. An electronic catheter-associated urinary tract infection surveillance tool. Infect Control Hosp Epidemiol. 2011;32(8):757-62. PMID: 21768758. doi: 10.1086/661103.

124. Dubberke ER, Nyazee HA, Yokoe DS, Mayer J, Stevenson KB, Mangino JE, et al. Implementing automated surveillance for tracking Clostridium difficile infection at multiple healthcare facilities. Infect Control Hosp Epidemiol. 2012;33(3):305-8. PMID: 22314071. doi: 10.1086/664052.

125. Ehrentraut C, Ekholm M, Tanushi H, Tiedemann J, Dalianis H. Detecting hospital-acquired infections: A document classification approach using support vector machines and gradient tree boosting. Health Informatics Journal. 2018;24(1):24-42. PMID: WOS:000424053900003. doi: 10.1177/1460458216656471.

126. FitzHenry F, Murff HJ, Matheny ME, Gentry N, Fielstein EM, Brown SH, et al. Exploring the Frontier of Electronic Health Record Surveillance The Case of Postoperative Complications. Medical Care. 2013;51(6):509-16. PMID: WOS:000319045800007. doi: 10.1097/MLR.0b013e31828d1210.

127. Fu S, Wyles CC, Osmon DR, Carvour ML, Sagheb E, Ramazanian T, et al. Automated Detection of Periprosthetic Joint Infections and Data Elements Using Natural Language Processing. J Arthroplasty. 2021;36(2):688-92. PMID: 32854996. doi: 10.1016/j.arth.2020.07.076.

128. Leal J, Gregson DB, Ross T, Flemons WW, Church DL, Laupland KB. Development of a novel electronic surveillance system for monitoring of bloodstream infections. Infect Control Hosp Epidemiol. 2010;31(7):740-7. PMID: 20470039. doi: 10.1086/653207.

129. Leal JR, Gregson DB, Church DL, Henderson EA, Ross T, Laupland KB. The Validation of a Novel Surveillance System for Monitoring Bloodstream Infections in the Calgary Zone. Can J Infect Dis Med Microbiol. 2016;2016:2935870. PMID: 27375749. doi: 10.1155/2016/2935870.

130. Leclère B, Lasserre C, Bourigault C, Juvin ME, Chaillet MP, Mauduit N, et al. Matching bacteriological and medico-administrative databases is efficient for a computer-enhanced surveillance of surgical site infections: retrospective analysis of 4,400 surgical procedures in a French university hospital. Infect Control Hosp Epidemiol. 2014;35(11):1330-5. PMID: 25333426. doi: 10.1086/678422.

131. Leth RA, Norgaard M, Uldbjerg N, Thomsen RW, Moller JK. Surveillance of selected post-caesarean infections based on electronic registries: validation study including post-discharge infections. Journal of Hospital Infection. 2010;75(3):200-4. PMID: WOS:000279892700010. doi: 10.1016/j.jhin.2009.11.018.

132. Lin MY, Woeltje KF, Khan YM, Hota B, Doherty JA, Borlawsky TB, et al. Multicenter evaluation of computer automated versus traditional surveillance of hospital-acquired bloodstream infections. Infect Control Hosp Epidemiol. 2014;35(12):1483-90. PMID: 25419770. doi: 10.1086/678602.

133. Peterson KE, Hacek DM, Robicsek A, Thomson RB, Jr., Peterson LR. Electronic surveillance for infectious disease trend analysis following a quality improvement intervention. Infect Control Hosp Epidemiol. 2012;33(8):790-5. PMID: 22759546. doi: 10.1086/666625.

134. Redder JD, Leth RA, Møller JK. Incidence rates of hospital-acquired urinary tract and bloodstream infections generated by automated compilation of electronically available healthcare data. J Hosp Infect. 2015 Nov;91(3):231-6. PMID: 26162918. doi: 10.1016/j.jhin.2015.05.011.

135. Sakji S, Gicquel Q, Pereira S, Kergourlay I, Proux D, Darmoni S, et al. Evaluation of a French medical multi-terminology indexer for the manual annotation of natural language medical reports of healthcare-associated infections. Stud Health Technol Inform. 2010;160(Pt 1):252-6. PMID: 20841688.

136. Suzuki H, Clore GS, Perencevich EN, Hockett-Sherlock SM, Goto M, Nair R, et al. Development of a fully automated surgical site infection detection algorithm for use in cardiac and orthopedic surgery research. Infect Control Hosp Epidemiol. 2021;42(10):1215-20. PMID: 33618788. doi: 10.1017/ice.2020.1387.

137. Thirukumaran CP, Zaman A, Rubery PT, Calabria C, Li Y, Ricciardi BF, et al. Natural Language Processing for the Identification of Surgical Site Infections in Orthopaedics. Journal of Bone and Joint Surgery-American Volume. 2019;101(24):2167-74. PMID: WOS:000509673200009. doi: 10.2106/jbjs.19.00661.

138. Tvardik N, Kergourlay I, Bittar A, Segond F, Darmoni S, Metzger MH. Accuracy of using natural language processing methods for identifying healthcare-associated infections. International Journal of Medical Informatics. 2018;117:96-102. PMID: WOS:000439320600012. doi: 10.1016/j.ijmedinf.2018.06.002.

139. Valik JK, Ward L, Tanushi H, Müllersdorf K, Ternhag A, Aufwerber E, et al. Validation of automated sepsis surveillance based on the Sepsis-3 clinical criteria against physician record review in a general hospital population: observational study using electronic health records data. BMJ Qual Saf. 2020;29(9):735-45. PMID: 32029574. doi: 10.1136/bmjqs-2019-010123.

140. van der Werff SD, Thiman E, Tanushi H, Valik JK, Henriksson A, Ul Alam M, et al. The accuracy of fully automated algorithms for surveillance of healthcare-associated urinary tract infections in hospitalized patients. J Hosp Infect. 2021;110:139-47. PMID: 33548370. doi: 10.1016/j.jhin.2021.01.023.

141. Venable A, Dissanaike S. Is automated electronic surveillance for healthcare-associated infections accurate in the burn unit? J Burn Care Res. 2013;34(6):591-7. PMID: 24121803. doi: 10.1097/BCR.0b013e3182a2aa0f.

142. Wald HL, Bandle B, Richard A, Min S. Accuracy of electronic surveillance of catheter-associated urinary tract infection at an academic medical center. Infect Control Hosp Epidemiol. 2014;35(6):685-91. PMID: 24799645. doi: 10.1086/676429.

143. Woeltje KF, McMullen KM, Butler AM, Goris AJ, Doherty JA. Electronic surveillance for healthcare-associated central line-associated bloodstream infections outside the intensive care unit. Infect Control Hosp Epidemiol. 2011;32(11):1086-90. PMID: 22011535. doi: 10.1086/662181.

144. van Mourik MS, Groenwold RH, Berkelbach van der Sprenkel JW, van Solinge WW, Troelstra A, Bonten MJ. Automated detection of external ventricular and lumbar drain-related meningitis using laboratory and microbiology results and medication data. PLoS One. 2011;6(8):e22846. PMID: 21829659. doi: 10.1371/journal.pone.0022846.

145. Colborn KL, Zhuang Y, Dyas AR, Henderson WG, Madsen HJ, Bronsert MR, et al. Development and validation of models for detection of postoperative infections using structured electronic health records data and machine learning. Surgery. 2023 Feb;173(2):464-71. PMID: 36470694. doi: 10.1016/j.surg.2022.10.026.

146. Stern SE, Christensen MA, Nevers MR, Ying J, McKenna C, Munro S, et al. Electronic surveillance criteria for non-ventilator-associated hospital-acquired pneumonia: Assessment of reliability and validity. Infect Control Hosp Epidemiol. 2023 Mar 15:1-7. PMID: 36920040. doi: 10.1017/ice.2022.302.

147. van der Werff SD, Fritzing M, Tanushi H, Henriksson A, Dalianis H, Ternhag A, et al. The accuracy of fully automated algorithms for surveillance of healthcare-onset Clostridioides difficile infections in hospitalized patients. Antimicrob Steward Healthc Epidemiol. 2022;2(1):e43. PMID: 36310782. doi: 10.1017/ash.2022.32.
